# Complications and resource utilization in trauma patients with diabetes

**DOI:** 10.1371/journal.pone.0221414

**Published:** 2019-08-28

**Authors:** Katherine He, Mark R. Hemmila, Anne H. Cain-Nielsen, David A. Machado-Aranda, Lynn M. Frydrych, Matthew J. Delano

**Affiliations:** 1 Department of Surgery, Brigham and Women’s Hospital, Boston, MA, United States of America; 2 Department of Surgery, Division of Acute Care Surgery, University of Michigan, Ann Arbor, MI, United States of America; 3 Michigan Trauma Quality Improvement Program, Ann Arbor, MI, United States of America; University of Florida, UNITED STATES

## Abstract

Diabetes is associated with poor outcomes in critically ill populations. The goal of this study was to determine if diabetic patients suffer poorer outcomes following trauma. Collaborative trauma patient data from 2012–2018 was analyzed. Patients with no signs-of-life, Injury Severity Score (ISS) <5, age <16 years, and hospitalization <1 day were excluded. Multivariable logistic and linear regression were used to compare patients with and without diabetes for selected outcomes. Risk-adjustment variables included demographics, physiology, comorbidities, and injury scoring. Of 106,141 trauma patients, 14,150 (13%) had diabetes. On admission, diabetes was associated with significantly increased risk of any, serious, infectious, urinary tract, sepsis, pneumonia, and cardiac complications. Diabetes was also associated with increased ventilator days (7.5 vs. 6.6 days, p = 0.003), intensive care unit days (5.8 vs. 5.3 days, p<0.001), and hospital length of stay (5.7 vs. 5.3 days, p<0.001). Subgroup analysis revealed the least injured diabetic category (ISS 5–15) suffered higher odds of hospital mortality and any, serious, infectious and cardiac complications. The association between diabetes, hospital mortality and complication rates in mild traumatic injury is independent of age. Trauma patients with diabetes experience higher rates of complications and resource utilization. The largest cohort of diabetics experience the least severe injuries and suffer the greatest in hospital mortality and complication rates. A better understanding of the physiologic derangements associated with diabetes is necessary to develop novel approaches to reduce excess trauma morbidity, mortality and resource consumption.

## Background

With the growing ubiquity of the western diet and lifestyle, diabetes mellitus (DM) has become a common disease. Diabetes prevalence in the United States has almost tripled from 11.9 million people in 2000 to 30.3 million people in 2017, and the incidence has more than tripled from 1980–2017.[1]

Patients with DM are living longer and accumulating comorbidities that result in greater physiologic frailty. It has been well described that patients with complex comorbidities fare worse following traumatic injury.[2–4]. DM has been identified to be a leading global cause of years lived with disability (YLD) and in the United States is attributable to over 11% of deaths.[4,5]. This is surely an underestimate of the impact of DM, given one in four patients with DM is undiagnosed.[6] Dwindling healthcare dollars combined with increased DM morbidity will saddle an exhausted healthcare system and make attaining quality outcomes more challenging.

Trauma is a leading cause of death and nonfatal injury with almost 40 million emergency department visits annually in the United States[7]. The most recent data from the National Vital Statistics System at the Centers for Disease Control and Prevention National Center for Health Statistics report trauma as the #1 leading cause of death for the 1–44 year-old population and unintentional falls as the #1 leading cause of nonfatal injuries for all emergency department patients. The number of patients dying from traumatic injury and treated for unintentional falls has risen abruptly over the past decade, with over half of the increase attributable to patients over age 65.[8]

While several investigations have addressed the impact of hyperglycemia and glucose control on trauma outcomes,[9–12] there is a paucity of studies that address DM and its association with complications in traumatically injured patients. The studies that do examine the association between comorbid DM and trauma outcomes are limited in their ability to account for all confounders or outcomes.[13,14]

Given the increasing incidence, prevalence, and YLD of DM, the outcomes of trauma patients presenting with comorbid DM must be better characterized to more definitively demonstrate the contribution of DM to adverse trauma outcomes and resource utilization. The objective of this study is to use data from the Michigan Trauma Quality Improvement Program to examine the impact of DM on outcomes in trauma patients.

## Methods

This project was approved by the University of Michigan Institutional Review Board. The data was initially collected for the purposes of Quality Improvement–this project is secondary use of these data. The data were fully anonymized and a waiver of informed consent was obtained.

### Michigan Trauma Quality Improvement Program (MTQIP)

The Michigan Trauma Quality Improvement Program (MTQIP) is a statewide collaborative quality initiative that is sponsored by Blue Cross Blue Shield of Michigan/Blue Care Network (BCBSM/BCM) and includes 29 American College of Surgeons Committee on Trauma verified level 1 and 2 trauma centers. It receives over 19,000 case submissions per year and utilizes a data definitions manual that is updated annually and references existing national sources (National Surgical Quality Improvement Program, National Trauma Data Standard, Centers for Disease Control) when possible to achieve data consistency. The University of Michigan serves as the coordinating center for MTQIP.

### Data

Data were abstracted from the MTQIP database for the years 2012–2018. Study inclusion criteria included age ≥16, primary mechanism of injury classified as blunt or penetrating, Injury Severity Scoring (ISS) ≥5, hospital admission ≥24 hours, and discharge disposition known. Patients with no signs of life when initially evaluated (systolic blood pressure = 0 mmHg, pulse = 0 bpm, Glasgow Coma Scale Score (GCS) = 3) and burn patients were excluded.[15,16] Due to the complexities of diagnosing type 1 versus type 2 DM, MTQIP data are unable to differentiate the comorbid condition of DM by type. Patients were coded as diabetic if they self-reported DM, were on exogenous parenteral insulin or an oral hypoglycemic agent, or if they were previously diagnosed with DM per chart documentation

### Analysis

The primary outcome of interest was complication rates in patients with and without DM. Complication groupings analyzed included infection (incisional surgical site infection (SSI), organ space SSI, urinary tract infection (UTI), pneumonia, clostridium difficile infection, sepsis), cardiac (cardiac arrest requiring cardiopulmonary resuscitation—CPR, myocardial infarction—MI), renal (acute renal failure requiring dialysis—ARF), venous thromboembolism (VTE) (pulmonary embolism, deep vein thrombosis), and other (wound disruption, abdominal fascia left open, acute respiratory distress syndrome—ARDS, unplanned intubation, stroke/cerebral vascular accident, abdominal compartment syndrome—ACS, extremity compartment syndrome, decubitus ulcer, enterocutaneous fistula).

Complication rates for any complication, infectious complication, cardiac complication, acute renal failure, and VTE were compared between patients with and without DM using univariate methods (Pearson chi squared analysis and T-tests) with significance p ≤ 0.05. Multivariable logistic regression analysis was then used to compare complication rates for patients with and without DM for the same above complications with the addition of severe complications (ARDS, pneumonia, unplanned intubation, VTE, ARF, stroke, cardiac arrest requiring CPR, MI, sepsis, ACS, extremity compartment syndrome, decubitus ulcer, enterocutaneous fistula). Infectious complications were further separated into SSI, UTI, clostridium difficile infection, sepsis, and pneumonia. We then performed multivariable logistic regression analysis for the subset of patients age ≥65 to evaluate age as a predictor of outcome. Multivariable linear regression was then performed to compare average predicted ventilator-days, intensive care unit (ICU) days, and length of stay for patients with and without DM. Risk-adjustment variables included demographics (age, sex, race, transfer, blunt vs. penetrating trauma, history of smoking), physiology (GCS motor, Emergency Department (ED) pulse, ED blood pressure, abdominal fascia left open), comorbidities (DM, obesity, hypertension, dialysis dependence, drug use, history of cirrhosis, congestive heart failure, routine steroid use, peripheral vascular disease, metastatic cancer, active chemotherapy, ascites, history of psychiatric illness, actively anticoagulated), and injury scoring (ISS, Abbreviated Injury Score (AIS) ≥3 in head/neck, face, chest, abdomen, extremity). Statistical analyses were performed using SAS software (version 9.3, Cary, NC).

## Results

### Demographics

The final analysis included 106,141 patients. Baseline patient demographics are presented in **Table 1**. Patients with DM were significantly older, more likely to be white, and more likely to be female. DM patients were significantly more likely to experience comorbid congestive heart failure, peripheral vascular disease, hypertension, cirrhosis, obesity, and be dependent on dialysis.

**Table 1:** Patient demographics.

	No DM	DM	p-value
	(n = 91,991) 86.7%	(n = 14,150) 13.3%	
Age	54.1 ± 23.2	69.6 ± 14.9	<0.001
Male	61.2%	55.5%	<0.001
ISS	12.3 ± 8.4	11.6 ± 6.9	<0.001
Race (Non-White)	25.2%	19.0%	<0.001
Congestive heart failure	2.5%	8.1%	<0.001
PVD	0.7%	2.2%	<0.001
Hypertension	31.4%	75.3%	<0.001
Dialysis	0.7%	3.8%	<0.001
Cirrhosis	0.6%	1.3%	<0.001
Metastasis	0.4%	0.6%	<0.001
Active chemotherapy	0.3%	0.6%	<0.001
Obesity	6.3%	12.0%	<0.001
Ascites	0.03%	0.09%	<0.001
Drug use	15.0%	5.0%	<0.001
Smoker	28.1%	14.7%	<0.001
Psychiatric history	16.0%	18.5%	<0.001
Anticoagulated	10.3%	26.9%	<0.001
Blunt mechanism	91.7%	98.0%	<0.001
Transfer	18.0%	19.7%	<0.001

ISS = injury severity score. PVD = peripheral vascular disease. DM = diabetes mellitus.

### Complication rates

Complication rates and risk adjusted odds ratios of complications of patients with and without DM are presented in **Table 2 and Table 3.** Patients with DM were significantly more likely to experience any complication. They had significantly higher rates of infection, renal complications, and cardiac complications. Rates of VTE between DM and non-DM patients showed no significant difference. There was no significant in-hospital mortality difference. Diabetic trauma patients had significantly higher odds of developing any complication, a severe complication, any infection, any renal complication, and any cardiac complication during their hospitalization (OR range 1.21–1.61). Of the infectious complications, diabetic trauma patients had significantly increased odds of developing UTIs, sepsis, and pneumonia (OR range 1.21–1.45). Overall, DM patients had significantly higher odds of complications and in particular, were significantly more likely to develop infections including UTI, sepsis, and pneumonia.

**Table 2:** Complication rates of patients with and without DM.

	No Diabetes	Diabetes	p-value
	(n = 91,991)	(n = 14,150)	
Complications (Any)	7.5%	9.7%	<0.001
Mortality	4.6%	4.9%	0.09
Infection	4.4%	5.1%	<0.001
Cardiac	1.3%	1.9%	<0.001
Acute Renal Failure	0.4%	0.8%	<0.001
VTE	1.3%	1.3%	0.9

DM = diabetes mellitus. VTE = venous thromboembolism. DM = diabetes mellitus.

**Table 3:** Odds ratio of complications given DM.

	OR for Diabetes	[95% CI for OR]	p-value
Complications (Any)	1.27	[1.19, 1.36]	<0.001
Complications (Severe)	1.28	[1.30, 1.38]	<0.001
Mortality	1.13	[0.98, 1.30]	0.08
Infection	1.26	[1.14, 1.38]	<0.001
SSI	1.13	[0.79, 1.62]	0.5
UTI	1.21	[1.05, 1.39]	0.008
Cdiff	1.03	[0.74, 1.43]	0.2
Sepsis	1.45	[1.18, 1.79]	<0.001
Pneumonia	1.27	[1.14, 1.40]	<0.001
Cardiac	1.40	[1.19, 1.64]	<0.001
Acute Renal Failure	1.61	[1.27, 2.04]	<0.001
VTE	1.15	[0.99, 1.33]	0.07

SSI = surgical site infection. UTI = urinary tract infection. Cdiff = Clostridium difficile infection. VTE = venous thromboembolism. DM = diabetes mellitus. OR = odds ratio. CI = confidence interval.

### Complication OR, stratified by ISS

Interestingly, trends in increased complication risk in diabetic patients were primarily found in the subset of patients with the lowest Injury Severity Scale rating 5–15 in **Table 4**. Diabetic patients in this category had increased odds of all complication groupings, including death, any complication, serious complication, infection, and cardiac complication. For patients with ISS 16–24, diabetic patients were only at a greater odds of developing infectious complication (OR 1.25). Patients with ISS 25–35 had greater odds of developing any, serious, and cardiac complications (OR range 1.22–1.66).

**Table 4:** Outcomes for diabetic vs non-diabetic patients, stratified by ISS category.

Adjusted OR for DM (95% CI)				
	ISS 5–15	ISS 16–24	ISS 25–35	ISS >35
	n = 80289	n = 16097	n = 7742	n = 2013
Mortality	1.25 (1.06, 1.48)*	1.02 (0.80, 1.30)	1.08 (0.87, 1.32)	1.01 (0.56, 1.81)
Complications (Any)	1.37 (1.26, 1.48)*	1.13 (0.99, 1.29)	1.22 (1.01, 1.48)*	1.15 (0.78, 1.70)
Complications (Serious)	1.38 (1.28, 1.49)*	1.13(0.99, 1.29)	1.30 (1.07, 1.59)*	1.14 (0.76, 1.71)
Infection	1.32 (1.18, 1.48)*	1.25 (1.10, 1.44)*	1.13 (0.90, 1.42)	1.25 (0.85, 1.85)
Cardiac	1.43 (1.14, 1.79)*	1.33 (0.94, 1.89)	1.66 (1.20, 2.29)*	1.07 (0.60, 1.88)

ISS = injury severity score.

* Indicates statistically significant with p-value <0.05.

### Hospital length of stay (HLOS), ICU length of stay (ILOS), and ventilator-days

Adjusted effect of DM on length of stay and ventilator-days are presented in **Table 5.** 38% of DM patients and 37% of non-DM patients were admitted to the ICU (p-value = 0.23). 14% of DM patients and 13% of non-DM patients were mechanically ventilated (p-value = <0.001). Trauma patients with DM experienced a significantly longer ILOS (5.8 vs. 5.3 days, p<0.001), HLOS (5.7 vs. 5.3 days, p<0.001), and a greater number of ventilator-days (7.5 vs. 6.6 days, p = 0.003). Diabetic patients remained on the ventilator, stayed in the ICU, and stayed in the hospital longer than their counterparts without DM, indicating greater health system resource consumption than their non-diabetic counterparts.

**Table 5:** Hospital days, ICU days, and ventilator-days of patients with and without DM.

	No DM (n = 91,991)	DM (n = 14,150)	p-value
Mechanical Ventilation, %	12.9	13.7	<0.001
Admitted to ICU, %	37.0	37.7	0.23
Ventilator-Days*	6.55 ± 0.27	7.50 ± 0.40	0.003
ICU Days†	5.25 ± 0.21	5.78 ± 0.27	<0.001
Length of Stay‡	5.34 ± 0.17	5.69 ± 0.18	<0.001

ICU = intensive care unit. DM = DM mellitus.

* Average predicted, for patients with >0 ventilator-days

† Average predicted, for patients with >0 ICU days

‡ Average predicted

### Complication rates, age ≥65

Complication rates of patients ≥65 years old with and without DM are presented in **Table 6.** As compared to our entire patient sample, patients ≥65 with DM were likewise significantly more likely to suffer any complication, a severe complication, renal failure, a cardiac complication, or any infection (OR range 1.26–1.28). The infectious complications patients ≥65 with DM were significantly more likely to experience than their non-DM peers were sepsis and pneumonia. Overall, our subset of patients ≥65 with DM did not experience more complications than patients with DM in the entire study population. Although DM patients tend to be older, their increased risk of trauma complications is not due to the aging process but rather associated with the presence of DM.

**Table 6:** Odds ratio of complication given DM, age ≥65.

	OR for DM	[95% CI for OR]	p-value
Complications (Any)	1.26	[1.14, 1.37]	<0.001
Complications (Severe)	1.26	[1.14, 1.39]	<0.001
Mortality	1.08	[0.95, 1.24]	0.24
Infection	1.28	[1.13, 1.45]	<0.001
SSI	1.67	[0.84, 3.33]	0.15
UTI	1.15	[0.99, 1.34]	0.07
Cdiff	1.15	[0.74, 1.78]	0.5
Sepsis	1.69	[1.22, 2.32]	0.001
Pneumonia	1.26	[1.07, 1.48]	0.006
Cardiac	1.25	[1.06, 1.48]	0.008
Acute Renal Failure	1. 70	[1.28, 2.25]	<0.001
VTE	1.12	[0.86, 1.44]	0.4

SSI = surgical site infection. UTI = urinary tract infection. Cdiff = Clostridium difficile infection. VTE = venous thromboembolism. DM = DM mellitus. OR = odds ratio. CI = confidence interval.

### Full regression results and diagnostics

Full model specifications and results for all adjusted results (Tables 1, 3, 4, 5 and 6) are presented in **S1 Appendix**. Variance inflation factors (VIFs) to assess collinearity are also shown in **S1 Appendix**.

## Discussion

With the elderly, obese and diabetic populations expanding and populating our trauma centers, it is imperative that we understand the complications they are at risk for during hospitalization. Current literature clearly associates hyperglycemia with adverse outcomes in trauma and critical illness. However, DM has profound physiologic impacts in addition to hyperglycemia and contributes to immune suppression, physiologic frailty, and immune senescence which are all linked to poor outcomes. In this study, we report that trauma patients who are admitted with DM suffer an increased number and severity of complications, infections, and a greater number of ventilator-days and ILOS than those without DM. Most importantly, we have demonstrated that the largest DM group sustains the least severe trauma injuries (ISS 5–15) yet suffers higher odds of hospital mortality and complication rates compared with those in more severe injury categories. Lastly, we show that those who suffer from traumatic insults and have DM require more hospital resources to provide adequate care.

The association of hyperglycemia with adverse outcomes in the critically ill population is not novel.[17] Laird and colleagues performed a retrospective study of 516 ICU patients showing early stress hyperglycemia in critically ill patients leads to increased infection and mortality.[9] Bochicchio and coauthors examined prospective data on 942 trauma patients admitted to the ICU and found persistent hyperglycemia contributed to greater ILOS, HLOS, ventilator-days, infection rate, and mortality.[10] Chin performed a prospective study of 2,231 acutely injured patients and found tight glucose control to be associated with lower mortality and adverse outcomes.[11] However, these studies were designed to examine stress hyperglycemia and excluded patients with DM. While Yendamuri included DM patients in their study of 738 trauma patients showing admission hyperglycemia was associated with increased ILOS, HLOS, infectious morbidity, and mortality, the study did not separate diabetic from non-diabetic patients, precluding meaningful conclusions regarding the impact of DM on trauma outcomes.[12]

More recent studies have examined the association of comorbid DM and trauma outcomes but have not accounted for all confounders or outcomes. Kao examined 343,250 patients from the National Trauma Data Bank and showed DM was a weak risk factor for infectious morbidity and ILOS. However, their analysis was limited by inconsistent reporting of comorbidities between institutions and they did not examine the impact of DM on specific infectious complications.[13] Ahmad performed a retrospective analysis of 24,9778 matched trauma patients with and without DM and found greater ILOS, ventilator-days, and complications in patients with DM.[14] While their data was matched by age, sex, and ISS, many other unidentified factors could have contributed to differences in outcome, including dialysis, mechanism of injury, and smoking status.

Our study’s findings build upon previous reports, which have likewise found DM to be associated with increased ICU length of stay[13,14] and 1-2-day increase in ventilator-days.[14] While our data show an increased average predicted total length of stay (TLOS), literature has been inconsistent, in one case associating DM with a longer TLOS,[13] and in another case showing no difference in TLOS between patients with and without DM. In all studies, DM and hyperglycemia have been associated with significantly increased odds of multiple infectious complications.[9,12–14] Consistent with our results, DM has further been found to significantly increase a patient’s odds of developing infections like sepsis, UTI, wound infection, pneumonia, and decubitus ulcers.[14] However, our study uniquely examined the association of DM with severe complications including ARDS, pneumonia, unplanned intubation, VTE, ARF, stroke, cardiac arrest requiring CPR, MI, sepsis, ACS, extremity compartment syndrome, decubitus ulcer, and enterocutaneous fistula.

It is evident that DM patients require more healthcare resources than their non-diabetic counterparts and that DM has a substantial impact on the healthcare economy. The American DM Association performed a prevalence-based cost study in 2012 and found patients with DM spent 2.3 times more on medical expenditures than non-diabetic patients.[18] Their estimated total cost of diagnosed DM in 2012 was $245 billion, which included $176 billion in direct medical costs and $69 billion in reduced productivity. Inpatient hospital care comprised 43% of medical costs and was the largest burden of medical expenditures. These statistics are unsurprising, given our findings associating DM with poorer trauma outcomes.

Our finding that statistical increased mortality in diabetic patients was only found in mildly injured patients (ISS <15) warrants additional attention. In our study, DM patients with severe injury defined as ISS >15 had no statistically significant differences in mortality as compared to their non-diabetic counterparts. Increased odds for any, severe, and cardiac complications were significant in ISS 25–35 diabetic patients, however we were unable to evaluate clear patterns in mortality in patients with ISS >15 due to lower prevalence of ISS >15 injury as compared to ISS 5–15 injury (25,852 with ISS >15 vs. 80,289 with ISS ≤15). Other studies have included ISS in their analyses but have matched by ISS[14] or used ISS as a continuous rather than categorical variable [13], which overlooks the non-linear relationship between ISS and outcomes. Future studies are necessary to determine if differences in mortality between non-diabetic and DM patients are consistent between severely injured (ISS >15) and non-severely injured patients.

The negative impact of increased ventilator-days as seen in DM patients in our study is clear. In previous studies, increased ventilator-days has been shown to be associated with increased ventilator-associated pneumonia (VAP), increased costs,[19,20] longer ICU stay, and increased mortality.[21,22] Arthur 2016’s review of 12 studies with 3,571 participants found the estimated attributable mortality of VAP to be 13%. Cook 1998 and Arthur 2016 found that 10–20% of patients who are mechanically ventilated for greater than 24 hours developed VAP.[22,23] According to 2016 Infectious Diseases Society of America and the American Thoracic Society clinical guidelines, empiric antibiotic treatment for VAP necessitates broad-spectrum antibiotic coverage for *Pseudomonas aeruginosa*, other gram-negative bacilli, and often methicillin resistant *S*. *aureus*, depending on local antimicrobial resistant rates.[24] Empiric antibiotic regimens are not free from harm—rates of superinfection on empiric antibiotic coverage have been found to be up to 21% and adverse effects as high as 49%, depending on the choice of antibiotic.[22] Not only are DM patients more intrinsically susceptible to infection, they are also more likely to experience prolonged ventilator courses in comparison to their non-DM peers, and thus more likely to contract VAP and be exposed to the adverse drugs effects of broad-spectrum antibiotics.

Several reports have examined the connection between increased infectious morbidity and DM and have hypothesized that diabetic patients are predisposed to infection due to impaired neutrophil function, decreased adaptive immune response, and dysfunctional immune cell function through high serum levels of inflammatory mediators.[25,26] DM patients are also associated with bacterial pathogens with increased antibiotic resistance such as MRSA, *Pseudomonas*, and *Acinetobacter*, which are associated with ICU-related mortality.[27] Several of these effects are exacerbated by the hyperglycemia seen in poorly controlled diabetic patients [25] and improved with insulin administration.[26,28] Surgical ICU patients randomized to intensive insulin therapy in Van den Berghe’s study of 1,548 patients experienced a significant decrease in hospital mortality of 32%.[29] Of note, this reduction in mortality was not found in the medical ICU.[29,30] Further research is warranted on other immunomodulatory interventions to address the immune perturbations caused by DM.

There are several limitations associated with working with a large database like the MTQIP database. The MTQIP is a prospectively collected and retrospectively analyzed dataset. The diagnosis of DM in our case was broad-based and not predicated on the American Diabetes Association diagnosis criteria. Patients were coded as diabetic by self-report, exogenous parenteral insulin or oral hypoglycemic agent use, or by previous DM diagnosis per chart documentation. The MTQIP database was unable to distinguish between patients with Type 1 and Type 2 DM and did not have data on length of diabetic history, which are significant factors in development of DM-related complications. As the incidence of DM in our study is 13%, slightly higher than the 9% DM population-based incidence nationwide,[6] we can reasonably infer that the vast majority, over 90%, of our study patients have Type II DM and not Type I DM. In-hospital glycemic data was not available, and as such, we were unable to compare outcomes between DM/non-DM patients and hyperglycemic/euglycemic patients. Additionally, due to the nature of the data collected, we were unable to comment on the rate of new diagnosis of DM in our cohort, which would be informative given the tendency of trauma for unmasking undiagnosed DM. Finally, our dataset included in-hospital mortality only. Due to the low frequency of death and short overall hospital stay in our cohort, we only unable to detect a difference in mortality between diabetic and non-diabetic patients in mildly injured patients with ISS <15. Further studies are warranted to investigate mortality trends in diabetic patients suffering trauma.

Future directions of study include evaluating outcomes in DM populations undergoing acute surgery, separating the impact of DM from hyperglycemia, and examining differences in outcomes between Type 1 and Type 2 DM or between insulin-dependent DM and non-insulin-dependent DM. Further studies warrant investigating proactive infection prevention strategies such as immune modulatory therapy or prophylactic antibiotic therapy in DM cohorts.[31,32] With continued improvements in biomarker technology, cellular function determination and host/pathogen genomic interaction modeling, strategically-engineered combinations of immune modulators could be deployed based on patient comorbidity profiles, immune function, and recovery course. There is a tremendous need to elucidate the cellular interactions between DM and the immunologic system that predispose DM patients to infectious morbidity and to develop pharmacologic interventions to decrease DM patients’ risk of infection, recidivism, healthcare cost, and death.

## Conclusions

We found DM to be an independent risk factor for adverse outcomes in trauma including infection, sepsis, cardiac complications, acute renal failure, ventilator-days, ICU days, and total length of stay. In subgroup analysis, these trends were primarily seen in non-severely injured patients with ISS <16 and ISS 25–35. Notably, increased mortality in diabetic patients was only seen in ISS<15 patients. As DM rates increase worldwide, the trauma patient population will become increasingly co-morbidly challenged, physiologically frail, and susceptible to infectious morbidity with bacteria that are progressively resistant to conventional infection management. A better understanding of the physiologic pathways affecting immunologic systems in DM trauma patients is vital to developing non-pharmacologic and pharmacologic interventions to decrease infectious morbidity and mortality in DM patients.

## Supporting information

S1 Appendix Full analyses. (DOCX)
