# Supplementary material for: Complications and resource utilization in trauma patients with diabetes
Source: PLoS One. 2019 Aug 28;14(8):e0221414. doi: 10.1371/journal.pone.0221414 (PMC6713394; doi:10.1371/journal.pone.0221414)
Supplement: S1 Appendix — (DOCX) [file pone.0221414.s001.docx]

**S1 Appendix.** Full analyses.

1 .

2 .

3 .

1. .
2. .

6 .

1. . tab comp_any diabetes, col chi2

Key

*frequency column percentage*

| comp_any | (max) diabetes | |
| --- | --- | --- |
|  | 0 1 | Total |
| 0 | **85,073 12,838**  **92.48 90.73** | **97,911**  **92.25** |
| 1 | **6,918 1,312**  **7.52 9.27** | **8,230**  **7.75** |
| Total | **91,991 14,150**  **100.00 100.00** | **106,141**  **100.00** |

Pearson chi2(**1**) = **52.6156** Pr = **0.000**

1. . tab comp_sev_any diabetes, col chi2

Key

*frequency column percentage*

| comp_sev_a  ny | (max) diabetes | |
| --- | --- | --- |
|  | 0 1 | Total |
| 0 | **86,074 13,022**  **93.57 92.03** | **99,096**  **93.36** |
| 1 | **5,917 1,128**  **6.43 7.97** | **7,045**  **6.64** |
| Total | **91,991 14,150**  **100.00 100.00** | **106,141**  **100.00** |

Pearson chi2(**1**) = **46.9086** Pr = **0.000**

1. . tab dead diabetes, col chi2

Key

*frequency column percentage*

| dead | (max) diabetes | |
| --- | --- | --- |
|  | 0 1 | Total |
| 0 | **87,810 13,462**  **95.45 95.14** | **101,272**  **95.41** |
| 1 | **4,181 688**  **4.55 4.86** | **4,869**  **4.59** |
| Total | **91,991 14,150**  **100.00 100.00** | **106,141**  **100.00** |

Pearson chi2(**1**) = **2.8188** Pr = **0.093**

1. . tab comp_infection diabetes, col chi2

Key

*frequency column percentage*

| comp_infec  tion | (max) diabetes | |
| --- | --- | --- |
|  | 0 1 | Total |
| 0 | **87,940 13,424**  **95.60 94.87** | **101,364**  **95.50** |
| 1 | **4,051 726**  **4.40 5.13** | **4,777**  **4.50** |
| Total | **91,991 14,150**  **100.00 100.00** | **106,141**  **100.00** |

Pearson chi2(**1**) = **15.0826** Pr = **0.000**

1. . tab comp_cardiac diabetes, col chi2

Key

*frequency column percentage*

| comp_cardi  ac | (max) diabetes | |
| --- | --- | --- |
|  | 0 1 | Total |
| 0 | **90,791 13,876**  **98.70 98.06** | **104,667**  **98.61** |
| 1 | **1,200 274**  **1.30 1.94** | **1,474**  **1.39** |
| Total | **91,991 14,150**  **100.00 100.00** | **106,141**  **100.00** |

Pearson chi2(**1**) = **35.7604** Pr = **0.000**

1. . tab acute_renal_failure diabetes, col chi2

Key

*frequency column percentage*

| (max) acute_rena l_failure | (max) | diabetes | |
| --- | --- | --- | --- |
|  | 0 | 1 | Total |
| 0 | **91,583** | **14,031** | **105,614** |
|  | **99.56** | **99.16** | **99.50** |
| 1 | **408** | **119** | **527** |
|  | **0.44** | **0.84** | **0.50** |
| Total | **91,991** | **14,150** | **106,141** |
|  | **100.00** | **100.00** | **100.00** |

Pearson chi2(**1**) = **39.2154** Pr = **0.000**

1. . tab comp_vte diabetes, col chi2

Key

*frequency column percentage*

| comp_vte | (max) diabetes | |
| --- | --- | --- |
|  | 0 1 | Total |
| 0 | **90,814 13,967**  **98.72 98.71** | **104,781**  **98.72** |
| 1 | **1,177 183**  **1.28 1.29** | **1,360**  **1.28** |
| Total | **91,991 14,150**  **100.00 100.00** | **106,141**  **100.00** |

Pearson chi2(**1**) = **0.0185** Pr = **0.892**

14 .

15 .

16 .

1. . /* Characteristics of Patients with and without diabetes */
2. . encode sex, gen(sex_num)
3. . gen missing_pulse=(pulse==.)
4. . gen missing_gcs=(gcsmcat==.)
5. . gen missing_bp=(bp==.) 22 .

23 . gen male=(sex=="M")

24 .

1. . gen pulse2=pulse

(2,676 missing values generated)

1. . replace pulse2=99 if pulse==. (2,676 real changes made)

27 .

1. . gen bp2=bp

(2,949 missing values generated)

1. . replace bp2=99 if bp==. (2,949 real changes made)

30 .

1. . gen gcsmcat2=gcsmcat

(7,230 missing values generated)

1. . replace gcsmcat2=99 if gcsmcat==. (7,230 real changes made)

33 .

1. . gen blunt_ind=1
2. . replace blunt_ind=0 if blunt ~="Blunt" (7,882 real changes made)

36 .

37 . global elixvars congestive_heart_failure pvd hypertension dialysis documented_history_of_cirrhos

- psych ac_coag routine_steroid_use

38 .

1. . **Table 1 Data
2. . tab diabetes

| (max) diabetes | Freq. | Percent | Cum. |
| --- | --- | --- | --- |
| 0 | **91,991** | **86.67** | **86.67** |
| 1 | **14,150** | **13.33** | **100.00** |
| Total | **106,141** | **100.00** |  |

41 .

42 . foreach X of varlist age iss {

1. di in red "`X'"
2. di
3. ttest `X', by(diabetes)
4. di
5. di

7. }

# age

Two-sample t test with equal variances

| Group | Obs | Mean | Std. Err. | Std. Dev. | [95% Conf. | Interval] |
| --- | --- | --- | --- | --- | --- | --- |
| 0 | **91,991** | **54.09068** | **.076517** | **23.20762** | **53.94071** | **54.24065** |
| 1 | **14,150** | **69.64093** | **.1255794** | **14.93814** | **69.39478** | **69.88709** |
| combined | **106,141** | **56.16374** | **.0702946** | **22.90147** | **56.02596** | **56.30151** |
| diff |  | **-15.55025** | **.2012191** |  | **-15.94464** | **-15.15587** |

diff = mean(**0**) - mean(**1**) t = **-77.2802**

Ho: diff = 0 degrees of freedom = **106139**

Ha: diff < 0 Ha: diff != 0 Ha: diff > 0 Pr(T < t) = **0.0000** Pr(|T| > |t|) = **0.0000** Pr(T > t) = **1.0000**

# iss

Two-sample t test with equal variances

| Group | Obs | Mean | Std. Err. | Std. Dev. | [95% Conf. | Interval] |
| --- | --- | --- | --- | --- | --- | --- |
| 0 | **91,991** | **12.3467** | **.0276265** | **8.379125** | **12.29255** | **12.40084** |
| 1 | **14,149** | **11.55644** | **.0583414** | **6.939682** | **11.44208** | **11.67079** |
| combined | **106,140** | **12.24135** | **.0251886** | **8.206215** | **12.19198** | **12.29072** |
| diff |  | **.7902619** | **.0740655** |  | **.6450944** | **.9354293** |

diff = mean(**0**) - mean(**1**) t = **10.6698**

Ho: diff = 0 degrees of freedom = **106138**

| Ha: diff < 0 | Ha: diff | != 0 | Ha: diff > 0 |
| --- | --- | --- | --- |
| Pr(T < t) = **1.0000** | Pr(\|T\| > \|t\|) | = **0.0000** | Pr(T > t) = **0.0000** |

43 .

44 . foreach X of varlist agecat male isscat aishn aisfac aischs aisabd aisext gcsmcat2 pulse2 bp2 ra

1. di in red "`X'"
2. di
3. tab `X' diabetes , col chi2 5. }

# agecat

Key

*frequency column percentage*

| agecat | (max) | diabetes | |
| --- | --- | --- | --- |
|  | 0 | 1 | Total |
| 18-25 | **13,226** | **117** | **13,343** |
|  | **14.38** | **0.83** | **12.57** |
| 26-45 | **21,875** | **750** | **22,625** |
|  | **23.78** | **5.30** | **21.32** |
| 46-65 | **25,489** | **4,165** | **29,654** |
|  | **27.71** | **29.43** | **27.94** |
| 66-75 | **9,543** | **3,422** | **12,965** |
|  | **10.37** | **24.18** | **12.21** |
| >75 | **21,858** | **5,696** | **27,554** |
|  | **23.76** | **40.25** | **25.96** |
| Total | **91,991** | **14,150** | **106,141** |
|  | **100.00** | **100.00** | **100.00** |

Pearson chi2(**4**) = **7.0e+03** Pr = **0.000**

# male

Key

*frequency column percentage*

| male | (max) | diabetes | |
| --- | --- | --- | --- |
|  | 0 | 1 | Total |
| 0 | **35,704** | **6,301** | **42,005** |
|  | **38.81** | **44.53** | **39.57** |
| 1 | **56,287** | **7,849** | **64,136** |
|  | **61.19** | **55.47** | **60.43** |
| Total | **91,991** | **14,150** | **106,141** |
|  | **100.00** | **100.00** | **100.00** |

Pearson chi2(**1**) = **167.6490** Pr = **0.000**

# isscat

Key

*frequency column percentage*

| isscat | (max) diabetes | |
| --- | --- | --- |
|  | 0 1 | Total |
| 5-15 | **69,264 11,025**  **75.29 77.92** | **80,289**  **75.64** |
| 16-24 | **14,064 2,033**  **15.29 14.37** | **16,097**  **15.17** |
| 25-35 | **6,770 972**  **7.36 6.87** | **7,742**  **7.29** |

| >35 | **1,893**  **2.06** | **120**  **0.85** | **2,013**  **1.90** |
| --- | --- | --- | --- |
| Total | **91,991** | **14,150** | **106,141** |
|  | **100.00** | **100.00** | **100.00** |

Pearson chi2(**3**) = **116.6693** Pr = **0.000**

# aishn

Key

*frequency column percentage*

| aishn | (max) diabetes | |
| --- | --- | --- |
|  | 0 1 | Total |
| 0 | **69,757 10,236**  **75.83 72.34** | **79,993**  **75.36** |
| 1 | **22,234 3,914**  **24.17 27.66** | **26,148**  **24.64** |
| Total | **91,991 14,150**  **100.00 100.00** | **106,141**  **100.00** |

Pearson chi2(**1**) = **80.5007** Pr = **0.000**

# aisfac

Key

*frequency column percentage*

| aisfac | (max) diabetes | |
| --- | --- | --- |
|  | 0 1 | Total |
| 0 | **91,484 14,113**  **99.45 99.74** | **105,597**  **99.49** |
| 1 | **507 37**  **0.55 0.26** | **544**  **0.51** |
| Total | **91,991 14,150**  **100.00 100.00** | **106,141**  **100.00** |

Pearson chi2(**1**) = **20.1791** Pr = **0.000**

# aischs

Key

*frequency column percentage*

| aischs | (max) diabetes | |
| --- | --- | --- |
|  | 0 1 | Total |
| 0 | **71,527 11,230**  **77.75 79.36** | **82,757**  **77.97** |
| 1 | **20,464 2,920**  **22.25 20.64** | **23,384**  **22.03** |
| Total | **91,991 14,150**  **100.00 100.00** | **106,141**  **100.00** |

Pearson chi2(**1**) = **18.4971** Pr = **0.000**

# aisabd

Key

*frequency column percentage*

| aisabd | (max) diabetes | |
| --- | --- | --- |
|  | 0 1 | Total |
| 0 | **85,867 13,658**  **93.34 96.52** | **99,525**  **93.77** |
| 1 | **6,124 492**  **6.66 3.48** | **6,616**  **6.23** |
| Total | **91,991 14,150**  **100.00 100.00** | **106,141**  **100.00** |

Pearson chi2(**1**) = **212.2023** Pr = **0.000**

# aisext

Key

*frequency column percentage*

| aisext | (max) diabetes | |
| --- | --- | --- |
|  | 0 1 | Total |
| 0 | **73,939 11,197**  **80.38 79.13** | **85,136**  **80.21** |
| 1 | **18,052 2,953**  **19.62 20.87** | **21,005**  **19.79** |
| Total | **91,991 14,150**  **100.00 100.00** | **106,141**  **100.00** |

Pearson chi2(**1**) = **11.9869** Pr = **0.001**

# gcsmcat2

Key

*frequency column percentage*

| gcsmcat2 | (max) diabetes | |
| --- | --- | --- |
|  | 0 1 | Total |
| 0 | **76,951 12,041**  **83.65 85.10** | **88,992**  **83.84** |
| 1 | **4,863 571**  **5.29 4.04** | **5,434**  **5.12** |
| 2 | **4,162 323**  **4.52 2.28** | **4,485**  **4.23** |
| 99 | **6,015 1,215**  **6.54 8.59** | **7,230**  **6.81** |
| Total | **91,991 14,150**  **100.00 100.00** | **106,141**  **100.00** |

Pearson chi2(**3**) = **261.8920** Pr = **0.000**

# pulse2

Key

*frequency column percentage*

| pulse2 | (max) diabetes | |
| --- | --- | --- |
|  | 0 1 | Total |
| 0 | **83,410 13,150**  **90.67 92.93** | **96,560**  **90.97** |
| 1 | **5,404 464**  **5.87 3.28** | **5,868**  **5.53** |
| 2 | **906 131**  **0.98 0.93** | **1,037**  **0.98** |
| 99 | **2,271 405**  **2.47 2.86** | **2,676**  **2.52** |
| Total | **91,991 14,150**  **100.00 100.00** | **106,141**  **100.00** |

Pearson chi2(**3**) = **164.2766** Pr = **0.000**

# bp2

Key

*frequency column percentage*

| bp2 | (max) | diabetes | |
| --- | --- | --- | --- |
|  | 0 | 1 | Total |
| 0 | **86,507** | **13,381** | **99,888** |
|  | **94.04** | **94.57** | **94.11** |
| 1 | **2,462** | **307** | **2,769** |
|  | **2.68** | **2.17** | **2.61** |
| 2 | **494** | **41** | **535** |
|  | **0.54** | **0.29** | **0.50** |
| 99 | **2,528** | **421** | **2,949** |
|  | **2.75** | **2.98** | **2.78** |
| Total | **91,991** | **14,150** | **106,141** |
|  | **100.00** | **100.00** | **100.00** |

Pearson chi2(**3**) = **29.5851** Pr = **0.000**

# race

Key

*frequency column percentage*

| race | (max) diabetes | |
| --- | --- | --- |
|  | 0 1 | Total |
| 0 | **68,794 11,461**  **74.78 81.00** | **80,255**  **75.61** |
| 1 | **23,197 2,689**  **25.22 19.00** | **25,886**  **24.39** |
| Total | **91,991 14,150**  **100.00 100.00** | **106,141**  **100.00** |

Pearson chi2(**1**) = **256.7199** Pr = **0.000**

# blunt_ind

Key

*frequency column percentage*

| blunt_ind | (max) diabetes | |
| --- | --- | --- |
|  | 0 1 | Total |
| 0 | **7,594 288**  **8.26 2.04** | **7,882**  **7.43** |
| 1 | **84,397 13,862**  **91.74 97.96** | **98,259**  **92.57** |
| Total | **91,991 14,150**  **100.00 100.00** | **106,141**  **100.00** |

Pearson chi2(**1**) = **690.1314** Pr = **0.000**

# transfer

Key

*frequency column percentage*

| transfer | (max) diabetes | |
| --- | --- | --- |
|  | 0 1 | Total |
| 0 | **75,409 11,367**  **81.97 80.33** | **86,776**  **81.76** |
| 1 | **16,582 2,783**  **18.03 19.67** | **19,365**  **18.24** |
| Total | **91,991 14,150**  **100.00 100.00** | **106,141**  **100.00** |

Pearson chi2(**1**) = **22.1719** Pr = **0.000 congestive_heart_failure**

Key

*frequency column percentage*

| (max) congestive  _heart_fai  lure | (max) | diabetes | |
| --- | --- | --- | --- |
|  | 0 | 1 | Total |
| 0 | **89,724** | **13,004** | **102,728** |
|  | **97.54** | **91.90** | **96.78** |
| 1 | **2,267** | **1,146** | **3,413** |
|  | **2.46** | **8.10** | **3.22** |
| Total | **91,991** | **14,150** | **106,141** |
|  | **100.00** | **100.00** | **100.00** |

Pearson chi2(**1**) = **1.3e+03** Pr = **0.000**

# pvd

Key

*frequency column percentage*

| (max) pvd | (max) diabetes | |
| --- | --- | --- |
|  | 0 1 | Total |
| 0 | **91,371 13,844**  **99.33 97.84** | **105,215**  **99.13** |
| 1 | **620 306**  **0.67 2.16** | **926**  **0.87** |
| Total | **91,991 14,150**  **100.00 100.00** | **106,141**  **100.00** |

Pearson chi2(**1**) = **314.2187** Pr = **0.000 hypertension**

Key

*frequency column percentage*

| (max) hypertensi  on | (max) | diabetes | |
| --- | --- | --- | --- |
|  | 0 | 1 | Total |
| 0 | **63,122** | **3,497** | **66,619** |
|  | **68.62** | **24.71** | **62.76** |
| 1 | **28,869** | **10,653** | **39,522** |
|  | **31.38** | **75.29** | **37.24** |
| Total | **91,991** | **14,150** | **106,141** |
|  | **100.00** | **100.00** | **100.00** |

Pearson chi2(**1**) = **1.0e+04** Pr = **0.000**

# dialysis

Key

*frequency column percentage*

| (max) dialysis | (max) diabetes | |
| --- | --- | --- |
|  | 0 1 | Total |
| 0 | **91,352 13,610**  **99.31 96.18** | **104,962**  **98.89** |
| 1 | **639 540**  **0.69 3.82** | **1,179**  **1.11** |
| Total | **91,991 14,150**  **100.00 100.00** | **106,141**  **100.00** |

Pearson chi2(**1**) = **1.1e+03** Pr = **0.000 documented_history_of_cirrhosis**

Key

*frequency column percentage*

| (max) documented  _history_o f_cirrhosi  s | (max) | diabetes | |
| --- | --- | --- | --- |
|  | 0 | 1 | Total |
| 0 | **91,434** | **13,963** | **105,397** |
|  | **99.39** | **98.68** | **99.30** |
| 1 | **557** | **187** | **744** |
|  | **0.61** | **1.32** | **0.70** |
| Total | **91,991** | **14,150** | **106,141** |
|  | **100.00** | **100.00** | **100.00** |

Pearson chi2(**1**) = **90.3407** Pr = **0.000**

# metastasis

Key

*frequency column percentage*

| (max) metastasis | (max) diabetes | |
| --- | --- | --- |
|  | 0 1 | Total |
| 0 | **91,653 14,066**  **99.63 99.41** | **105,719**  **99.60** |
| 1 | **338 84**  **0.37 0.59** | **422**  **0.40** |
| Total | **91,991 14,150**  **100.00 100.00** | **106,141**  **100.00** |

Pearson chi2(**1**) = **15.8472** Pr = **0.000 active_chemotherapy**

Key

*frequency column percentage*

| (max) active_che motherapy | (max) | diabetes | |
| --- | --- | --- | --- |
|  | 0 | 1 | Total |
| 0 | **91,694** | **14,067** | **105,761** |
|  | **99.68** | **99.41** | **99.64** |
| 1 | **297** | **83** | **380** |
|  | **0.32** | **0.59** | **0.36** |
| Total | **91,991** | **14,150** | **106,141** |
|  | **100.00** | **100.00** | **100.00** |

Pearson chi2(**1**) = **23.9081** Pr = **0.000**

# obesity

Key

*frequency column percentage*

| (max) obesity | (max) diabetes | |
| --- | --- | --- |
|  | 0 1 | Total |
| 0 | **86,200 12,457**  **93.70 88.04** | **98,657**  **92.95** |
| 1 | **5,791 1,693**  **6.30 11.96** | **7,484**  **7.05** |
| Total | **91,991 14,150**  **100.00 100.00** | **106,141**  **100.00** |

Pearson chi2(**1**) = **601.4648** Pr = **0.000**

# ascites

Key

*frequency column percentage*

| (max) ascites | (max) diabetes | |
| --- | --- | --- |
|  | 0 1 | Total |
| 0 | **91,965 14,137**  **99.97 99.91** | **106,102**  **99.96** |
| 1 | **26 13**  **0.03 0.09** | **39**  **0.04** |
| Total | **91,991 14,150**  **100.00 100.00** | **106,141**  **100.00** |

Pearson chi2(**1**) = **13.5094** Pr = **0.000**

# drug_use

Key

*frequency column percentage*

| (max) drug_use | (max) diabetes | |
| --- | --- | --- |
|  | 0 1 | Total |
| 0 | **78,152 13,444**  **84.96 95.01** | **91,596**  **86.30** |
| 1 | **13,839 706**  **15.04 4.99** | **14,545**  **13.70** |
| Total | **91,991 14,150**  **100.00 100.00** | **106,141**  **100.00** |

Pearson chi2(**1**) = **1.0e+03** Pr = **0.000**

# smoker

Key

*frequency column percentage*

| (max) smoker | (max) diabetes | |
| --- | --- | --- |
|  | 0 1 | Total |
| 0 | **66,117 12,064**  **71.87 85.26** | **78,181**  **73.66** |
| 1 | **25,874 2,086**  **28.13 14.74** | **27,960**  **26.34** |
| Total | **91,991 14,150**  **100.00 100.00** | **106,141**  **100.00** |

Pearson chi2(**1**) = **1.1e+03** Pr = **0.000**

# psych

Key

*frequency column percentage*

| (max) psych | (max) diabetes | |
| --- | --- | --- |
|  | 0 1 | Total |
| 0 | **77,292 11,526**  **84.02 81.46** | **88,818**  **83.68** |
| 1 | **14,699 2,624**  **15.98 18.54** | **17,323**  **16.32** |
| Total | **91,991 14,150**  **100.00 100.00** | **106,141**  **100.00** |

Pearson chi2(**1**) = **59.0993** Pr = **0.000**

# ac_coag

Key

*frequency column percentage*

| ac_coag | (max) diabetes | |
| --- | --- | --- |
|  | 0 1 | Total |
| 0 | **82,489 10,350**  **89.67 73.14** | **92,839**  **87.47** |
| 1 | **9,502 3,800**  **10.33 26.86** | **13,302**  **12.53** |
| Total | **91,991 14,150**  **100.00 100.00** | **106,141**  **100.00** |

Pearson chi2(**1**) = **3.1e+03** Pr = **0.000 routine_steroid_use**

Key

*frequency column percentage*

| (max) routine_st eroid_use | (max) | diabetes | |
| --- | --- | --- | --- |
|  | 0 | 1 | Total |
| 0 | **90,736** | **13,785** | **104,521** |
|  | **98.64** | **97.42** | **98.47** |
| 1 | **1,255** | **365** | **1,620** |
|  | **1.36** | **2.58** | **1.53** |
| Total | **91,991** | **14,150** | **106,141** |
|  | **100.00** | **100.00** | **100.00** |

Pearson chi2(**1**) = **120.5012** Pr = **0.000**

45 .

1. . ttest male, by(diabetes)

Two-sample t test with equal variances

| Group | Obs | Mean | Std. Err. | Std. Dev. | [95% Conf. | Interval] |
| --- | --- | --- | --- | --- | --- | --- |
| 0 | **91,991** | **.6118751** | **.0016067** | **.4873259** | **.6087259** | **.6150243** |
| 1 | **14,150** | **.5546996** | **.0041782** | **.4970165** | **.5465098** | **.5628895** |
| combined | **106,141** | **.6042528** | **.001501** | **.4890129** | **.6013109** | **.6071948** |
| diff |  | **.0571754** | **.0044123** |  | **.0485273** | **.0658236** |

diff = mean(**0**) - mean(**1**) t = **12.9580**

Ho: diff = 0 degrees of freedom = **106139**

| Ha: diff < 0 | Ha: diff | != 0 | Ha: diff > 0 |
| --- | --- | --- | --- |
| Pr(T < t) = **1.0000** | Pr(\|T\| > \|t\|) | = **0.0000** | Pr(T > t) = **0.0000** |

1. . ttest blunt_ind, by(diabetes)

Two-sample t test with equal variances

| Group | Obs | Mean | Std. Err. | Std. Dev. | [95% Conf. | Interval] |
| --- | --- | --- | --- | --- | --- | --- |
| 0 | **91,991** | **.9174484** | **.0009074** | **.2752047** | **.91567** | **.9192269** |
| 1 | **14,150** | **.9796466** | **.0011871** | **.1412109** | **.9773198** | **.9819735** |
| combined | **106,141** | **.9257403** | **.0008048** | **.2621943** | **.9241629** | **.9273177** |
| diff |  | **-.0621982** | **.0023599** |  | **-.0668236** | **-.0575728** |

diff = mean(**0**) - mean(**1**) t = **-26.3559**

Ho: diff = 0 degrees of freedom = **106139**

| Ha: | diff < 0 | Ha: diff | != 0 | Ha: diff > 0 |
| --- | --- | --- | --- | --- |
| Pr(T < | t) = **0.0000** | Pr(\|T\| > \|t\|) | = **0.0000** | Pr(T > t) = **1.0000** |

1. . ttest transfer, by(diabetes)

Two-sample t test with equal variances

| Group | Obs | Mean | Std. Err. | Std. Dev. | [95% Conf. | Interval] |
| --- | --- | --- | --- | --- | --- | --- |
| 0 | **91,991** | **.1802568** | **.0012674** | **.3844033** | **.1777727** | **.1827409** |
| 1 | **14,150** | **.1966784** | **.0033416** | **.3975012** | **.1901284** | **.2032285** |
| combined | **106,141** | **.182446** | **.0011855** | **.3862135** | **.1801225** | **.1847695** |
| diff |  | **-.0164217** | **.0034872** |  | **-.0232565** | **-.0095868** |

diff = mean(**0**) - mean(**1**) t = **-4.7092**

Ho: diff = 0 degrees of freedom = **106139**

| Ha: diff < 0 | Ha: | diff | != 0 | Ha: diff > 0 |
| --- | --- | --- | --- | --- |
| Pr(T < t) = **0.0000** | Pr(\|T\| > | \|t\|) | = **0.0000** | Pr(T > t) = **1.0000** |

49 .

50 .

1. . **Table 2 Data
2. . foreach X of varlist dead comp_any comp_infection comp_cardiac acute_renal_failure comp_vte{
3. di in red "`X'"
4. di
5. tab `X' (diabetes), col chi2
6. di
7. di

7. }

# dead

Key

*frequency column percentage*

| dead | (max) diabetes | |
| --- | --- | --- |
|  | 0 1 | Total |
| 0 | **87,810 13,462**  **95.45 95.14** | **101,272**  **95.41** |
| 1 | **4,181 688**  **4.55 4.86** | **4,869**  **4.59** |
| Total | **91,991 14,150**  **100.00 100.00** | **106,141**  **100.00** |

Pearson chi2(**1**) = **2.8188** Pr = **0.093**

# comp_any

Key

*frequency column percentage*

| comp_any | (max) diabetes | |
| --- | --- | --- |
|  | 0 1 | Total |
| 0 | **85,073 12,838**  **92.48 90.73** | **97,911**  **92.25** |
| 1 | **6,918 1,312**  **7.52 9.27** | **8,230**  **7.75** |
| Total | **91,991 14,150**  **100.00 100.00** | **106,141**  **100.00** |

Pearson chi2(**1**) = **52.6156** Pr = **0.000**

# comp_infection

Key

*frequency column percentage*

| comp_infec  tion | (max) diabetes | |
| --- | --- | --- |
|  | 0 1 | Total |
| 0 | **87,940 13,424**  **95.60 94.87** | **101,364**  **95.50** |
| 1 | **4,051 726**  **4.40 5.13** | **4,777**  **4.50** |
| Total | **91,991 14,150**  **100.00 100.00** | **106,141**  **100.00** |

Pearson chi2(**1**) = **15.0826** Pr = **0.000**

# comp_cardiac

Key

*frequency column percentage*

| comp_cardi  ac | (max) diabetes | |
| --- | --- | --- |
|  | 0 1 | Total |
| 0 | **90,791 13,876**  **98.70 98.06** | **104,667**  **98.61** |
| 1 | **1,200 274**  **1.30 1.94** | **1,474**  **1.39** |
| Total | **91,991 14,150**  **100.00 100.00** | **106,141**  **100.00** |

Pearson chi2(**1**) = **35.7604** Pr = **0.000**

# acute_renal_failure

Key

*frequency column percentage*

| (max) acute_rena l_failure | (max) | diabetes | |
| --- | --- | --- | --- |
|  | 0 | 1 | Total |
| 0 | **91,583** | **14,031** | **105,614** |
|  | **99.56** | **99.16** | **99.50** |
| 1 | **408** | **119** | **527** |
|  | **0.44** | **0.84** | **0.50** |
| Total | **91,991** | **14,150** | **106,141** |
|  | **100.00** | **100.00** | **100.00** |

Pearson chi2(**1**) = **39.2154** Pr = **0.000**

# comp_vte

Key

*frequency column percentage*

| comp_vte | (max) diabetes | |
| --- | --- | --- |
|  | 0 1 | Total |
| 0 | **90,814 13,967**  **98.72 98.71** | **104,781**  **98.72** |
| 1 | **1,177 183**  **1.28 1.29** | **1,360**  **1.28** |
| Total | **91,991 14,150**  **100.00 100.00** | **106,141**  **100.00** |

Pearson chi2(**1**) = **0.0185** Pr = **0.892**

53 .

54 .

1. . **Table 3 Data: Odds ratio of complications given DM.
2. . **Report OR for diabetes 57 .

58 . **Appendix data: full model results from these regressions 59 .

1. . gen date1=date(ed_arrdate,"DM20Y")
2. . gen year_admit=year(date1)

62 .

63 .

1. . foreach X of varlist comp_any comp_sev_any dead comp_infection comp_ssi urinary_tract_infection
   - omp_vte{ 2.
2. . di in red "`X'"
3. di
4. logit `X' diabetes i.agecat male i.isscat aishn aisfac aischs aisabd aisext i.gcsmcat
   - cluster traumactr)
5. di
6. di

7.

66 .

67 . }

# comp_any

Iteration 0: log pseudolikelihood = **-28946.312**

Iteration 1: log pseudolikelihood = **-27643.632**

Iteration 2: log pseudolikelihood = **-25446.59**

Iteration 3: log pseudolikelihood = **-24303.553**

Iteration 4: log pseudolikelihood = **-24082.209**

Iteration 5: log pseudolikelihood = **-24081.712**

Iteration 6: log pseudolikelihood = **-24081.712**

| Logistic regression | Number of obs | = | **106,141** |
| --- | --- | --- | --- |
|  | Wald chi2(33) | = | **.** |
|  | Prob > chi2 | = | **.** |
| Log pseudolikelihood = **-24081.712** | Pseudo R2 | = | **0.1681** |

(Std. Err. adjusted for **35** clusters in traumactr)

| comp_any | Odds Ratio | Robust Std. Err. | z | P>\|z\| | [95% Conf. | Interval] |
| --- | --- | --- | --- | --- | --- | --- |
| diabetes | **1.273809** | **.0425058** | **7.25** | **0.000** | **1.193165** | **1.359903** |
| agecat 26-45 | **1.158656** | **.0485803** | **3.51** | **0.000** | **1.067247** | **1.257893** |
| 46-65 | **1.646486** | **.0857201** | **9.58** | **0.000** | **1.486766** | **1.823366** |
| 66-75 | **1.989009** | **.120071** | **11.39** | **0.000** | **1.767063** | **2.238832** |
| >75 | **1.880613** | **.1167353** | **10.18** | **0.000** | **1.665186** | **2.12391** |
| male | **1.289136** | **.0488709** | **6.70** | **0.000** | **1.196823** | **1.38857** |
| isscat 16-24 | **2.316393** | **.0899691** | **21.63** | **0.000** | **2.146602** | **2.499615** |
| 25-35 | **3.677959** | **.2269706** | **21.10** | **0.000** | **3.258955** | **4.150834** |
| >35 | **4.418322** | **.4378894** | **14.99** | **0.000** | **3.638286** | **5.365596** |
| aishn | **1.3145** | **.0702203** | **5.12** | **0.000** | **1.183831** | **1.459593** |
| aisfac | **1.36909** | **.1900724** | **2.26** | **0.024** | **1.042939** | **1.797237** |
| aischs | **1.620648** | **.0811531** | **9.64** | **0.000** | **1.469147** | **1.787773** |
| aisabd | **1.867756** | **.1193054** | **9.78** | **0.000** | **1.647967** | **2.116859** |
| aisext | **1.915748** | **.0727625** | **17.12** | **0.000** | **1.778315** | **2.063802** |
| gcsmcat2  1 | **3.072322** | **.1244704** | **27.71** | **0.000** | **2.837799** | **3.326226** |
| 2 | **2.817994** | **.2362277** | **12.36** | **0.000** | **2.391031** | **3.321198** |
| 99 | **1.347307** | **.1280881** | **3.14** | **0.002** | **1.118261** | **1.623267** |
| pulse2 |  | | | | | |
| 1 | **1.701935** | **.0902658** | **10.03** | **0.000** | **1.533902** | **1.888375** |
| 2 | **1.15744** | **.1199403** | **1.41** | **0.158** | **.944696** | **1.418092** |
| 99 | **.5320404** | **.0970481** | **-3.46** | **0.001** | **.3721166** | **.7606942** |
| bp2 1 | **1.752947** | **.1008722** | **9.75** | **0.000** | **1.565983** | **1.962233** |
| 2 | **1.149057** | **.1714416** | **0.93** | **0.352** | **.8577105** | **1.539369** |
| 99 | **1.435396** | **.1946268** | **2.67** | **0.008** | **1.100415** | **1.87235** |
| race | **1.103804** | **.0577389** | **1.89** | **0.059** | **.9962461** | **1.222975** |
| blunt_ind | **.6641944** | **.040559** | **-6.70** | **0.000** | **.5892732** | **.7486413** |
| transfer | **1.163071** | **.09192** | **1.91** | **0.056** | **.9961715** | **1.357934** |
| congestive_heart_failure | **1.504802** | **.1201718** | **5.12** | **0.000** | **1.286777** | **1.759768** |
| pvd | **1.246333** | **.1312504** | **2.09** | **0.037** | **1.013899** | **1.532052** |
| hypertension | **1.070678** | **.0364408** | **2.01** | **0.045** | **1.001585** | **1.144536** |
| dialysis | **1.472074** | **.161932** | **3.52** | **0.000** | **1.186576** | **1.826266** |
| documented_history_of_cirrhosis | **2.050299** | **.2429699** | **6.06** | **0.000** | **1.625346** | **2.586357** |
| metastasis | **1.138897** | **.2441193** | **0.61** | **0.544** | **.7482241** | **1.733553** |
| active_chemotherapy | **.9565114** | **.2126027** | **-0.20** | **0.841** | **.6187206** | **1.478719** |
| obesity | **1.369213** | **.0832466** | **5.17** | **0.000** | **1.215399** | **1.542493** |
| ascites | **1.101233** | **.6926643** | **0.15** | **0.878** | **.3209831** | **3.778125** |
| drug_use | **1.111887** | **.0478185** | **2.47** | **0.014** | **1.022006** | **1.209673** |
| smoker | **1.073837** | **.0396555** | **1.93** | **0.054** | **.9988601** | **1.154443** |
| psych | **1.119178** | **.0503941** | **2.50** | **0.012** | **1.02464** | **1.222438** |
| ac_coag | **1.238301** | **.0705621** | **3.75** | **0.000** | **1.107445** | **1.384619** |
| routine_steroid_use | **1.375383** | **.1332486** | **3.29** | **0.001** | **1.137519** | **1.662988** |
| year_admit  2013 | **.9294452** | **.0549979** | **-1.24** | **0.216** | **.8276673** | **1.043739** |
| 2014 | **.9176387** | **.0637767** | **-1.24** | **0.216** | **.8007786** | **1.051553** |
| 2015 | **.9043965** | **.0791989** | **-1.15** | **0.251** | **.7617602** | **1.073741** |
| 2016 | **.8028979** | **.0691864** | **-2.55** | **0.011** | **.6781278** | **.9506248** |
| 2017 | **.7233286** | **.0704559** | **-3.33** | **0.001** | **.5976188** | **.8754816** |
| 2018 | **.6691202** | **.0702725** | **-3.83** | **0.000** | **.5446395** | **.8220518** |

_cons

**.0200685 .0025182 -31.15 0.000**

**.015693**

**.0256641**

Note: **_cons** estimates baseline odds.

# comp_sev_any

Iteration 0: log pseudolikelihood = **-25915.058**

Iteration 1: log pseudolikelihood = **-25841.377**

Iteration 2: log pseudolikelihood = **-23943.956**

Iteration 3: log pseudolikelihood = **-21483.511**

Iteration 4: log pseudolikelihood = **-21266.137**

Iteration 5: log pseudolikelihood = **-21264.497**

Iteration 6: log pseudolikelihood = **-21264.497**

Logistic regression Number of obs = **106,141**

Wald chi2(33) = **.**

Prob > chi2 = **.**

Log pseudolikelihood = **-21264.497** Pseudo R2 = **0.1795**

(Std. Err. adjusted for **35** clusters in traumactr)

comp_sev_any

diabetes

Robust

Odds Ratio Std. Err. z P>|z| [95% Conf. Interval]

# 1.288209 .0445414 7.32 0.000 1.203802 1.378535

agecat

| 26-45 | **1.204974** | **.0521116** | **4.31** | **0.000** | **1.107046** | **1.311565** |
| --- | --- | --- | --- | --- | --- | --- |
| 46-65 | **1.754647** | **.0983817** | **10.03** | **0.000** | **1.572039** | **1.958465** |
| 66-75 | **2.118972** | **.1396091** | **11.40** | **0.000** | **1.862274** | **2.411054** |
| >75 | **1.909655** | **.1370872** | **9.01** | **0.000** | **1.659014** | **2.198161** |
| male | **1.496434** | **.0517051** | **11.67** | **0.000** | **1.398449** | **1.601284** |
| isscat 16-24 | **2.412254** | **.0945655** | **22.46** | **0.000** | **2.23385** | **2.604905** |
| 25-35 | **3.971915** | **.2352823** | **23.28** | **0.000** | **3.536533** | **4.460896** |
| >35 | **4.840195** | **.4910562** | **15.54** | **0.000** | **3.967393** | **5.905008** |
| aishn | **1.313626** | **.0758368** | **4.73** | **0.000** | **1.17309** | **1.470999** |
| aisfac | **1.377032** | **.1900966** | **2.32** | **0.020** | **1.0506** | **1.80489** |
| aischs | **1.641612** | **.0899539** | **9.05** | **0.000** | **1.474443** | **1.827734** |
| aisabd | **1.686078** | **.102014** | **8.63** | **0.000** | **1.497534** | **1.89836** |
| aisext | **1.876043** | **.0705434** | **16.73** | **0.000** | **1.742753** | **2.019528** |
| gcsmcat2  1 | **3.126586** | **.1337962** | **26.64** | **0.000** | **2.875047** | **3.400133** |
| 2 | **3.090706** | **.2766307** | **12.61** | **0.000** | **2.593413** | **3.683356** |
| 99 | **1.443084** | **.1304806** | **4.06** | **0.000** | **1.208726** | **1.722882** |
| pulse2 |  | | | | | |
| 1 | **1.74886** | **.0915793** | **10.67** | **0.000** | **1.578272** | **1.937887** |
| 2 | **1.237197** | **.122035** | **2.16** | **0.031** | **1.019713** | **1.501067** |
| 99 | **.4784309** | **.0820549** | **-4.30** | **0.000** | **.3418464** | **.6695876** |
| bp2 1 | **1.763467** | **.1049141** | **9.54** | **0.000** | **1.569375** | **1.981563** |
| 2 | **1.225471** | **.1974157** | **1.26** | **0.207** | **.8936753** | **1.680453** |
| 99 | **1.578545** | **.2000705** | **3.60** | **0.000** | **1.231324** | **2.023677** |
| race | **1.114052** | **.0604721** | **1.99** | **0.047** | **1.001616** | **1.23911** |
| blunt_ind | **.7439794** | **.049585** | **-4.44** | **0.000** | **.6528745** | **.8477974** |
| transfer | **1.09851** | **.0813285** | **1.27** | **0.204** | **.9501344** | **1.270056** |
| congestive_heart_failure | **1.487366** | **.1196437** | **4.94** | **0.000** | **1.27042** | **1.74136** |
| pvd | **1.27054** | **.140112** | **2.17** | **0.030** | **1.023576** | **1.577091** |

| hypertension | **1.053303** | **.0406936** | **1.34** | **0.179** | **.97649** | **1.136159** |
| --- | --- | --- | --- | --- | --- | --- |
| dialysis | **1.56335** | **.1866404** | **3.74** | **0.000** | **1.237187** | **1.975499** |
| documented_history_of_cirrhosis | **1.923674** | **.2585618** | **4.87** | **0.000** | **1.478159** | **2.503467** |
| metastasis | **1.156569** | **.2605577** | **0.65** | **0.518** | **.7437184** | **1.7986** |
| active_chemotherapy | **1.083635** | **.2365522** | **0.37** | **0.713** | **.7064314** | **1.66225** |
| obesity | **1.452413** | **.0957667** | **5.66** | **0.000** | **1.276336** | **1.65278** |
| ascites | **1.165673** | **.8168164** | **0.22** | **0.827** | **.2952011** | **4.602939** |
| drug_use | **1.059579** | **.0443866** | **1.38** | **0.167** | **.9760581** | **1.150246** |
| smoker | **1.085895** | **.0438672** | **2.04** | **0.041** | **1.003233** | **1.175369** |
| psych | **1.122889** | **.0541063** | **2.41** | **0.016** | **1.021696** | **1.234104** |
| ac_coag | **1.206428** | **.0785177** | **2.88** | **0.004** | **1.061947** | **1.370566** |
| routine_steroid_use | **1.460861** | **.1475264** | **3.75** | **0.000** | **1.198532** | **1.780608** |
| year_admit  2013 | **.9183555** | **.0591574** | **-1.32** | **0.186** | **.8094298** | **1.041939** |
| 2014 | **.904889** | **.0719437** | **-1.26** | **0.209** | **.7743192** | **1.057476** |
| 2015 | **.9399565** | **.0827409** | **-0.70** | **0.482** | **.7910058** | **1.116956** |
| 2016 | **.8974404** | **.0814408** | **-1.19** | **0.233** | **.7512093** | **1.072137** |
| 2017 | **.8498916** | **.0782494** | **-1.77** | **0.077** | **.7095671** | **1.017967** |
| 2018 | **.7993103** | **.0830458** | **-2.16** | **0.031** | **.652046** | **.9798341** |
| _cons | **.0116853** | **.0014407** | **-36.09** | **0.000** | **.0091768** | **.0148795** |

Note: **_cons** estimates baseline odds.

# dead

Iteration 0: log pseudolikelihood = **-19761.247**

Iteration 1: log pseudolikelihood = **-14690.028**

Iteration 2: log pseudolikelihood = **-12210.336**

Iteration 3: log pseudolikelihood = **-11239.425**

Iteration 4: log pseudolikelihood = **-11197.754**

Iteration 5: log pseudolikelihood = **-11197.606**

Iteration 6: log pseudolikelihood = **-11197.606**

Logistic regression Number of obs = **106,141**

Wald chi2(33) = **.**

Prob > chi2 = **.**

Log pseudolikelihood = **-11197.606** Pseudo R2 = **0.4334**

(Std. Err. adjusted for **35** clusters in traumactr)

Robust dead Odds Ratio Std. Err.

z

P>|z|

[95% Conf. Interval]

diabetes

**1.132698 .0816911**

**1.73 0.084**

**.9833875**

**1.304678**

agecat

| 26-45 | **1.116398** | **.072278** | **1.70** | **0.089** | **.9833551** | **1.26744** |
| --- | --- | --- | --- | --- | --- | --- |
| 46-65 | **2.048334** | **.1480343** | **9.92** | **0.000** | **1.777804** | **2.360031** |
| 66-75 | **4.05897** | **.4302005** | **13.22** | **0.000** | **3.297608** | **4.996117** |
| >75 | **8.419763** | **.8790467** | **20.41** | **0.000** | **6.861705** | **10.3316** |
| male | **1.29875** | **.0624176** | **5.44** | **0.000** | **1.181999** | **1.427033** |
| isscat 16-24 | **2.672464** | **.2219788** | **11.83** | **0.000** | **2.270962** | **3.144951** |
| 25-35 | **9.683472** | **1.054978** | **20.84** | **0.000** | **7.821606** | **11.98854** |
| >35 | **16.53662** | **2.518888** | **18.42** | **0.000** | **12.26846** | **22.28965** |
| aishn | **1.232312** | **.0764058** | **3.37** | **0.001** | **1.091301** | **1.391543** |
| aisfac | **.493321** | **.0618943** | **-5.63** | **0.000** | **.3857749** | **.6308489** |
| aischs | **1.025692** | **.0594365** | **0.44** | **0.662** | **.9155708** | **1.149059** |
| aisabd | **1.052779** | **.0899418** | **0.60** | **0.547** | **.8904648** | **1.24468** |
| aisext | **.9357701** | **.0638353** | **-0.97** | **0.330** | **.8186586** | **1.069635** |

| gcsmcat2 |  | | | | | |
| --- | --- | --- | --- | --- | --- | --- |
| 1 | **4.798948** | **.3120462** | **24.12** | **0.000** | **4.224717** | **5.45123** |
| 2 | **25.10449** | **2.1254** | **38.07** | **0.000** | **21.26605** | **29.63575** |
| 99 | **1.44455** | **.1826042** | **2.91** | **0.004** | **1.127543** | **1.850684** |
| pulse2 |  |  |  |  |  |  |
| 1 | **1.809758** | **.1346125** | **7.98** | **0.000** | **1.564252** | **2.093795** |
| 2 | **2.102356** | **.2395327** | **6.52** | **0.000** | **1.681606** | **2.62838** |
| 99 | **1.604131** | **.2422659** | **3.13** | **0.002** | **1.193125** | **2.156719** |
| bp2 1 | **2.176322** | **.1586078** | **10.67** | **0.000** | **1.886638** | **2.510485** |
| 2 | **2.886159** | **.4720858** | **6.48** | **0.000** | **2.094547** | **3.976952** |
| 99 | **2.406336** | **.3943093** | **5.36** | **0.000** | **1.745323** | **3.317697** |
| race | **.9124419** | **.0628552** | **-1.33** | **0.183** | **.7972025** | **1.04434** |
| blunt_ind | **.2533927** | **.0195074** | **-17.83** | **0.000** | **.2179036** | **.2946618** |
| transfer | **.6358711** | **.0389751** | **-7.39** | **0.000** | **.5638915** | **.7170388** |
| congestive_heart_failure | **1.709391** | **.157071** | **5.83** | **0.000** | **1.427667** | **2.046708** |
| pvd | **1.630846** | **.2598422** | **3.07** | **0.002** | **1.193414** | **2.228613** |
| hypertension | **.8220812** | **.0475373** | **-3.39** | **0.001** | **.7339957** | **.9207377** |
| dialysis | **2.400958** | **.3762331** | **5.59** | **0.000** | **1.766038** | **3.264141** |
| documented_history_of_cirrhosis | **4.649885** | **.8545109** | **8.36** | **0.000** | **3.243521** | **6.666039** |
| metastasis | **2.570008** | **.5599987** | **4.33** | **0.000** | **1.676717** | **3.939212** |
| active_chemotherapy | **1.162973** | **.3133429** | **0.56** | **0.575** | **.6858487** | **1.972019** |
| obesity | **1.01838** | **.093375** | **0.20** | **0.843** | **.85087** | **1.218867** |
| ascites | **.8057286** | **.5342626** | **-0.33** | **0.745** | **.2196716** | **2.955314** |
| drug_use | **.5738354** | **.0443625** | **-7.18** | **0.000** | **.4931533** | **.6677174** |
| smoker | **.6021571** | **.0491966** | **-6.21** | **0.000** | **.5130576** | **.70673** |
| psych | **.9432169** | **.0488816** | **-1.13** | **0.259** | **.8521157** | **1.044058** |
| ac_coag | **1.364156** | **.1090291** | **3.89** | **0.000** | **1.16636** | **1.595496** |
| routine_steroid_use | **1.483066** | **.2202811** | **2.65** | **0.008** | **1.108487** | **1.984222** |
| year_admit  2013 | **.9956201** | **.0946369** | **-0.05** | **0.963** | **.8263883** | **1.199508** |
| 2014 | **.9999315** | **.0813069** | **-0.00** | **0.999** | **.8526228** | **1.172691** |
| 2015 | **1.089688** | **.0919401** | **1.02** | **0.309** | **.9236** | **1.285644** |
| 2016 | **1.098** | **.0915605** | **1.12** | **0.262** | **.9324427** | **1.292953** |
| 2017 | **1.047513** | **.0904368** | **0.54** | **0.591** | **.8844458** | **1.240646** |
| 2018 | **.8895046** | **.0759092** | **-1.37** | **0.170** | **.7525021** | **1.05145** |
| _cons | **.0082464** | **.0008728** | **-45.33** | **0.000** | **.0067014** | **.0101475** |

Note: **_cons** estimates baseline odds.

| **comp_infection** |  |  |  | | |
| --- | --- | --- | --- | --- | --- |
| Iteration 0: log pseudolikelihood | = | **-19481.118** |  |  |  |
| Iteration 1: log pseudolikelihood | = | **-16943.961** |  |  |  |
| Iteration 2: log pseudolikelihood | = | **-16431.005** |  |  |  |
| Iteration 3: log pseudolikelihood | = | **-16424.258** |  |  |  |
| Iteration 4: log pseudolikelihood | = | **-16424.253** |  |  |  |
| Iteration 5: log pseudolikelihood | = | **-16424.253** |  |  |  |
| Logistic regression  Log pseudolikelihood = **-16424.253** |  |  | Number of obs Wald chi2(33) Prob > chi2 Pseudo R2 | =  =  =  = | **106,141**  **.**  **. 0.1569** |

(Std. Err. adjusted for **35** clusters in traumactr)

| comp_infection | Odds Ratio | Robust Std. Err. | z | P>\|z\| | [95% Conf. | Interval] |
| --- | --- | --- | --- | --- | --- | --- |
| diabetes | **1.25515** | **.0599534** | **4.76** | **0.000** | **1.142976** | **1.378333** |
| agecat 26-45 | **1.115937** | **.0599911** | **2.04** | **0.041** | **1.004339** | **1.239935** |
| 46-65 | **1.423801** | **.1153719** | **4.36** | **0.000** | **1.214719** | **1.668872** |
| 66-75 | **1.579716** | **.1153134** | **6.26** | **0.000** | **1.36913** | **1.822693** |
| >75 | **1.431127** | **.1267574** | **4.05** | **0.000** | **1.203055** | **1.702435** |
| male | **1.18979** | **.0603067** | **3.43** | **0.001** | **1.077273** | **1.31406** |
| isscat 16-24 | **2.453299** | **.1476353** | **14.91** | **0.000** | **2.180352** | **2.760414** |
| 25-35 | **3.55235** | **.2936609** | **15.33** | **0.000** | **3.020993** | **4.177166** |
| >35 | **3.801992** | **.4056419** | **12.52** | **0.000** | **3.084572** | **4.686273** |
| aishn | **1.487131** | **.0850837** | **6.94** | **0.000** | **1.32938** | **1.663602** |
| aisfac | **1.532115** | **.261423** | **2.50** | **0.012** | **1.096609** | **2.140577** |
| aischs | **1.70505** | **.0883928** | **10.29** | **0.000** | **1.540314** | **1.887404** |
| aisabd | **1.766563** | **.109754** | **9.16** | **0.000** | **1.564031** | **1.995323** |
| aisext | **1.508651** | **.0642264** | **9.66** | **0.000** | **1.387879** | **1.639934** |
| gcsmcat2  1 | **3.28729** | **.2016896** | **19.40** | **0.000** | **2.914829** | **3.707345** |
| 2 | **2.530434** | **.2705556** | **8.68** | **0.000** | **2.052031** | **3.120369** |
| 99 | **1.320563** | **.1545318** | **2.38** | **0.017** | **1.049909** | **1.660988** |
| pulse2 |  | | | | | |
| 1 | **1.422395** | **.1165347** | **4.30** | **0.000** | **1.211386** | **1.670159** |
| 2 | **.7603857** | **.1005703** | **-2.07** | **0.038** | **.5867488** | **.9854071** |
| 99 | **.5272337** | **.1387701** | **-2.43** | **0.015** | **.3147483** | **.8831673** |
| bp2 1 | **1.438485** | **.1035622** | **5.05** | **0.000** | **1.249177** | **1.656482** |
| 2 | **.6432903** | **.1000277** | **-2.84** | **0.005** | **.474297** | **.8724964** |
| 99 | **1.183002** | **.2091521** | **0.95** | **0.342** | **.8365558** | **1.672923** |
| race | **1.101439** | **.0694675** | **1.53** | **0.126** | **.9733644** | **1.246366** |
| blunt_ind | **.7773796** | **.0713781** | **-2.74** | **0.006** | **.6493468** | **.9306567** |
| transfer | **1.231298** | **.125694** | **2.04** | **0.042** | **1.008022** | **1.504028** |
| congestive_heart_failure | **1.519072** | **.152483** | **4.17** | **0.000** | **1.247773** | **1.849359** |
| pvd | **1.138697** | **.1669549** | **0.89** | **0.376** | **.8542907** | **1.517787** |
| hypertension | **1.099705** | **.0266839** | **3.92** | **0.000** | **1.048629** | **1.153268** |
| dialysis | **.9242893** | **.1481762** | **-0.49** | **0.623** | **.6750699** | **1.265514** |
| documented_history_of_cirrhosis | **1.711622** | **.2105254** | **4.37** | **0.000** | **1.344968** | **2.178229** |
| metastasis | **1.25361** | **.3537251** | **0.80** | **0.423** | **.7210793** | **2.179425** |
| active_chemotherapy | **.8057001** | **.2850053** | **-0.61** | **0.541** | **.4027844** | **1.611663** |
| obesity | **1.361139** | **.1142095** | **3.67** | **0.000** | **1.15473** | **1.604443** |
| ascites | **1.20346** | **.7725858** | **0.29** | **0.773** | **.3419681** | **4.235239** |
| drug_use | **1.154246** | **.0596476** | **2.78** | **0.006** | **1.043064** | **1.277278** |
| smoker | **1.216122** | **.0540869** | **4.40** | **0.000** | **1.114603** | **1.326888** |
| psych | **1.211576** | **.0641069** | **3.63** | **0.000** | **1.092225** | **1.34397** |
| ac_coag | **1.225427** | **.0616396** | **4.04** | **0.000** | **1.11038** | **1.352395** |
| routine_steroid_use | **1.304653** | **.1780275** | **1.95** | **0.051** | **.9984896** | **1.704694** |
| year_admit  2013 | **.9082523** | **.0718833** | **-1.22** | **0.224** | **.7777473** | **1.060656** |
| 2014 | **.8740767** | **.075724** | **-1.55** | **0.120** | **.7375769** | **1.035838** |
| 2015 | **.8075504** | **.0897314** | **-1.92** | **0.054** | **.649513** | **1.004041** |
| 2016 | **.5672238** | **.0619112** | **-5.19** | **0.000** | **.4579813** | **.7025239** |
| 2017 | **.5148321** | **.0565255** | **-6.05** | **0.000** | **.4151535** | **.6384436** |
| 2018 | **.4824248** | **.0562722** | **-6.25** | **0.000** | **.3838325** | **.6063418** |

_cons

**.0131693 .0022043 -25.87 0.000**

**.0094861**

**.0182826**

Note: **_cons** estimates baseline odds.

# comp_ssi

note: metastasis != 0 predicts failure perfectly metastasis dropped and 422 obs not used

note: active_chemotherapy != 0 predicts failure perfectly active_chemotherapy dropped and 282 obs not used

note: ascites != 0 predicts failure perfectly ascites dropped and 37 obs not used

Iteration 0: log pseudolikelihood = **-3354.9002**

Iteration 1: log pseudolikelihood = **-3319.074**

Iteration 2: log pseudolikelihood = **-2701.8783**

Iteration 3: log pseudolikelihood = **-2681.006**

Iteration 4: log pseudolikelihood = **-2680.679**

Iteration 5: log pseudolikelihood = **-2680.6779**

Iteration 6: log pseudolikelihood = **-2680.6779**

| Logistic regression  Log pseudolikelihood | = | Number of obs Wald chi2(33) Prob > chi2  **-2680.6779** Pseudo R2 | | | | =  =  =  = | **105,400**  **.**  **. 0.2010** | | |
| --- | --- | --- | --- | --- | --- | --- | --- | --- | --- |
|  | | |  | (Std. Err. | adjusted | | for **35** | clusters in | traumactr) |
| comp_ssi | | | Odds Ratio | Robust Std. Err. | z | | P>\|z\| | [95% Conf. | Interval] |
| diabetes | | | **1.12745** | **.2079216** | **0.65** | | **0.515** | **.7854539** | **1.618355** |
| agecat 26-45 | | | **1.189377** | **.1542605** | **1.34** | | **0.181** | **.9224011** | **1.533625** |
| 46-65 | | | **1.233686** | **.1730919** | **1.50** | | **0.134** | **.937081** | **1.624173** |
| 66-75 | | | **.6812247** | **.1509108** | **-1.73** | | **0.083** | **.4412907** | **1.051613** |
| >75 | | | **.3369568** | **.0751138** | **-4.88** | | **0.000** | **.2176837** | **.5215819** |
| male | | | **1.361644** | **.1535094** | **2.74** | | **0.006** | **1.091694** | **1.698348** |
| isscat 16-24 | | | **2.669336** | **.2929842** | **8.95** | | **0.000** | **2.152663** | **3.310019** |
| 25-35 | | | **3.029673** | **.5558979** | **6.04** | | **0.000** | **2.114528** | **4.340882** |
| >35 | | | **3.111435** | **.717267** | **4.92** | | **0.000** | **1.980326** | **4.888605** |
| aishn | | | **.7299379** | **.0893247** | **-2.57** | | **0.010** | **.5742776** | **.9277906** |
| aisfac | | | **1.229839** | **.5739816** | **0.44** | | **0.658** | **.4926992** | **3.069834** |
| aischs | | | **1.127856** | **.1498239** | **0.91** | | **0.365** | **.8693215** | **1.463278** |
| aisabd | | | **4.013753** | **.5834515** | **9.56** | | **0.000** | **3.018682** | **5.336837** |
| aisext | | | **2.258658** | **.1876121** | **9.81** | | **0.000** | **1.919317** | **2.657996** |
| gcsmcat2  1 | | | **2.131915** | **.177822** | **9.08** | | **0.000** | **1.810388** | **2.510546** |
| 2 | | | **1.524011** | **.2670526** | **2.40** | | **0.016** | **1.081015** | **2.148544** |
| 99 | | | **1.63626** | **.4325098** | **1.86** | | **0.062** | **.9746656** | **2.746938** |
| pulse2 | | |  | | | | | | |
| 1 | | | **1.470517** | **.1346212** | **4.21** | **0.000** | | **1.228981** | **1.759523** |
| 2 | | | **.3198281** | **.1886067** | **-1.93** | **0.053** | | **.1006821** | **1.01597** |
| 99 | | | **.5508393** | **.3067804** | **-1.07** | **0.284** | | **.184911** | **1.640918** |
| bp2 | | |  |  |  |  | |  |  |

| 1 | **1.328723** | **.1806078** | **2.09** | **0.037** | **1.017969** | **1.734342** |
| --- | --- | --- | --- | --- | --- | --- |
| 2 | **.9151939** | **.2444593** | **-0.33** | **0.740** | **.5421842** | **1.544825** |
| 99 | **1.031703** | **.3622202** | **0.09** | **0.929** | **.5184495** | **2.053065** |
| race | **1.229313** | **.163646** | **1.55** | **0.121** | **.9470013** | **1.595785** |
| blunt_ind | **.2894075** | **.0465736** | **-7.70** | **0.000** | **.2111194** | **.3967268** |
| transfer | **1.266837** | **.2343508** | **1.28** | **0.201** | **.8815719** | **1.82047** |
| congestive_heart_failure | **.8882927** | **.45486** | **-0.23** | **0.817** | **.3256035** | **2.423389** |
| pvd | **2.421694** | **1.218749** | **1.76** | **0.079** | **.9031111** | **6.493777** |
| hypertension | **1.055599** | **.1517739** | **0.38** | **0.707** | **.7963674** | **1.399216** |
| dialysis | **.7170947** | **.5328237** | **-0.45** | **0.654** | **.1671504** | **3.076421** |
| documented_history_of_cirrhosis | **2.483435** | **.9268542** | **2.44** | **0.015** | **1.195011** | **5.160998** |
| metastasis | **1** | (omitted) |  |  |  |  |
| active_chemotherapy | **1** | (omitted) |  |  |  |  |
| obesity | **1.574233** | **.2690117** | **2.66** | **0.008** | **1.126191** | **2.200523** |
| ascites | **1** | (omitted) |  |  |  |  |
| drug_use | **1.459283** | **.1629155** | **3.39** | **0.001** | **1.172494** | **1.81622** |
| smoker | **1.270002** | **.1046622** | **2.90** | **0.004** | **1.080578** | **1.492632** |
| psych | **1.226251** | **.1654474** | **1.51** | **0.131** | **.9413129** | **1.59744** |
| ac_coag | **1.262016** | **.2767261** | **1.06** | **0.289** | **.8211439** | **1.939592** |
| routine_steroid_use | **1.722355** | **.7460422** | **1.26** | **0.209** | **.7369241** | **4.025526** |
| year_admit  2013 | **.8855334** | **.1818651** | **-0.59** | **0.554** | **.592094** | **1.3244** |
| 2014 | **1.220162** | **.2105387** | **1.15** | **0.249** | **.8700473** | **1.711166** |
| 2015 | **.9061049** | **.1669996** | **-0.53** | **0.593** | **.6313903** | **1.300347** |
| 2016 | **.9478156** | **.172661** | **-0.29** | **0.769** | **.6632278** | **1.354519** |
| 2017 | **.9187865** | **.1754179** | **-0.44** | **0.657** | **.6319756** | **1.335761** |
| 2018 | **.6898045** | **.1708859** | **-1.50** | **0.134** | **.4244799** | **1.120972** |
| _cons | **.0027178** | **.0007977** | **-20.13** | **0.000** | **.0015289** | **.0048311** |

Note: **_cons** estimates baseline odds.

# urinary_tract_infection

Iteration 0: log pseudolikelihood = **-6598.9384**

Iteration 1: log pseudolikelihood = **-5973.7677**

Iteration 2: log pseudolikelihood = **-5790.7465**

Iteration 3: log pseudolikelihood = **-5784.6393**

Iteration 4: log pseudolikelihood = **-5784.6209**

Iteration 5: log pseudolikelihood = **-5784.6209**

| Logistic regression  Log pseudolikelihood | Number of obs Wald chi2(33) Prob > chi2  = **-5784.6209** Pseudo R2 | | | | =  =  =  = | **106,141**  **.**  **. 0.1234** | | |
| --- | --- | --- | --- | --- | --- | --- | --- | --- |
| (Std. Err. adjusted for **35** clusters in traumactr) | | | | | | | | |
| urinary_tract_infection | | Odds Ratio | Robust Std. Err. | z | P>\|z\| | | [95% Conf. | Interval] |
| diabetes | | **1.210649** | **.0874614** | **2.65** | **0.008** | | **1.050811** | **1.3948** |
| agecat 26-45 | | **.8574264** | **.0901942** | **-1.46** | **0.144** | | **.6976819** | **1.053747** |
| 46-65 | | **1.339338** | **.1393116** | **2.81** | **0.005** | | **1.092326** | **1.642208** |
| 66-75 | | **1.692766** | **.2375412** | **3.75** | **0.000** | | **1.285731** | **2.228659** |
| >75 | | **1.871427** | **.2110833** | **5.56** | **0.000** | | **1.50025** | **2.334437** |
| male | | **.4617931** | **.044725** | **-7.98** | **0.000** | | **.3819513** | **.5583247** |
| isscat 16-24 | | **2.005431** | **.1917493** | **7.28** | **0.000** | | **1.662724** | **2.418775** |

| 25-35 | **2.886618** | **.4176498** | **7.33** | **0.000** | **2.173869** | **3.833056** |
| --- | --- | --- | --- | --- | --- | --- |
| >35 | **3.124675** | **.4935327** | **7.21** | **0.000** | **2.292769** | **4.25843** |
| aishn | **1.486838** | **.1251978** | **4.71** | **0.000** | **1.260634** | **1.753631** |
| aisfac | **1.133294** | **.3711671** | **0.38** | **0.702** | **.5964363** | **2.153382** |
| aischs | **1.529301** | **.1261234** | **5.15** | **0.000** | **1.301048** | **1.797598** |
| aisabd | **1.66606** | **.1814569** | **4.69** | **0.000** | **1.345808** | **2.06252** |
| aisext | **1.555642** | **.1027725** | **6.69** | **0.000** | **1.366708** | **1.770695** |
| gcsmcat2  1 | **2.342925** | **.2443839** | **8.16** | **0.000** | **1.90973** | **2.874384** |
| 2 | **1.375012** | **.1659913** | **2.64** | **0.008** | **1.0853** | **1.742062** |
| 99 | **.9660857** | **.1123382** | **-0.30** | **0.767** | **.7691949** | **1.213375** |
| pulse2 |  |  |  |  |  |  |
| 1 | **1.19205** | **.1069195** | **1.96** | **0.050** | **.9998777** | **1.421157** |
| 2 | **.7866085** | **.2060563** | **-0.92** | **0.360** | **.4707408** | **1.314424** |
| 99 | **.8742996** | **.2808578** | **-0.42** | **0.676** | **.4658246** | **1.640961** |
| bp2 1 | **1.517301** | **.1747128** | **3.62** | **0.000** | **1.210761** | **1.901451** |
| 2 | **.8952135** | **.2990213** | **-0.33** | **0.740** | **.4651642** | **1.722848** |
| 99 | **.9130745** | **.223207** | **-0.37** | **0.710** | **.5654894** | **1.474307** |
| race | **1.164472** | **.1318166** | **1.35** | **0.179** | **.932769** | **1.45373** |
| blunt_ind | **.9232936** | **.1996402** | **-0.37** | **0.712** | **.6043491** | **1.410561** |
| transfer | **1.32664** | **.1792249** | **2.09** | **0.036** | **1.018025** | **1.728812** |
| congestive_heart_failure | **1.505679** | **.2199437** | **2.80** | **0.005** | **1.130817** | **2.004807** |
| pvd | **1.34022** | **.3753946** | **1.05** | **0.296** | **.774025** | **2.320582** |
| hypertension | **1.063098** | **.0718989** | **0.90** | **0.366** | **.9311197** | **1.213784** |
| dialysis | **.8805449** | **.271639** | **-0.41** | **0.680** | **.4810217** | **1.611901** |
| documented_history_of_cirrhosis | **1.707666** | **.6765957** | **1.35** | **0.177** | **.7855027** | **3.712429** |
| metastasis | **1.646834** | **.5618014** | **1.46** | **0.144** | **.8438607** | **3.213875** |
| active_chemotherapy | **.6220648** | **.3477118** | **-0.85** | **0.396** | **.207991** | **1.860487** |
| obesity | **1.08234** | **.1335383** | **0.64** | **0.521** | **.8498514** | **1.378428** |
| ascites | **.6671865** | **.6042697** | **-0.45** | **0.655** | **.1130617** | **3.937124** |
| drug_use | **1.14645** | **.1177252** | **1.33** | **0.183** | **.93745** | **1.402046** |
| smoker | **1.038656** | **.0766851** | **0.51** | **0.607** | **.8987243** | **1.200375** |
| psych | **1.202905** | **.1106595** | **2.01** | **0.045** | **1.004445** | **1.440577** |
| ac_coag | **1.322663** | **.1128789** | **3.28** | **0.001** | **1.118938** | **1.563481** |
| routine_steroid_use | **1.20515** | **.2895209** | **0.78** | **0.437** | **.7525777** | **1.929881** |
| year_admit  2013 | **.8706246** | **.1063297** | **-1.13** | **0.257** | **.6852884** | **1.106085** |
| 2014 | **.7273075** | **.0986783** | **-2.35** | **0.019** | **.5574813** | **.948868** |
| 2015 | **.6622348** | **.1107129** | **-2.47** | **0.014** | **.4772077** | **.9190022** |
| 2016 | **.3149961** | **.0667368** | **-5.45** | **0.000** | **.2079529** | **.4771396** |
| 2017 | **.1450789** | **.0250048** | **-11.20** | **0.000** | **.1034897** | **.2033814** |
| 2018 | **.1551106** | **.0305964** | **-9.45** | **0.000** | **.1053749** | **.228321** |
| _cons | **.0088909** | **.0024101** | **-17.42** | **0.000** | **.0052264** | **.0151247** |

Note: **_cons** estimates baseline odds.

# cdiff

note: active_chemotherapy != 0 predicts failure perfectly active_chemotherapy dropped and 380 obs not used

Iteration 0: log pseudolikelihood = **-2691.3917**

Iteration 1: log pseudolikelihood = **-2497.3202**

Iteration 2: log pseudolikelihood = **-2453.363**

Iteration 3: log pseudolikelihood = **-2453.0587**

Iteration 4: log pseudolikelihood = **-2453.0581**

Iteration 5: log pseudolikelihood = **-2453.0581**

| Logistic regression  Log pseudolikelihood | = | Number of obs Wald chi2(33) Prob > chi2  **-2453.0581** Pseudo R2 | | | | =  =  =  = | **105,761**  **.**  **. 0.0886** | | |
| --- | --- | --- | --- | --- | --- | --- | --- | --- | --- |
| (Std. Err. adjusted for **35** clusters in traumactr) | | | | | | | | | |
| cdiff | | | Odds Ratio | Robust Std. Err. | z | P>\|z\| | | [95% Conf. | Interval] |
| diabetes | | | **1.027564** | **.1743491** | **0.16** | **0.873** | | **.7368579** | **1.432961** |
| agecat 26-45 | | | **.8602381** | **.1036731** | **-1.25** | **0.212** | | **.6792577** | **1.089439** |
| 46-65 | | | **.7289286** | **.1436168** | **-1.60** | **0.109** | | **.4954244** | **1.072488** |
| 66-75 | | | **.9857471** | **.2890896** | **-0.05** | **0.961** | | **.5547968** | **1.751447** |
| >75 | | | **.9583973** | **.2418301** | **-0.17** | **0.866** | | **.5844723** | **1.571546** |
| male | | | **1.462648** | **.2090522** | **2.66** | **0.008** | | **1.105299** | **1.935529** |
| isscat 16-24 | | | **2.576854** | **.3992893** | **6.11** | **0.000** | | **1.901928** | **3.491286** |
| 25-35 | | | **3.661335** | **.8293559** | **5.73** | **0.000** | | **2.3487** | **5.707572** |
| >35 | | | **3.249894** | **.6263634** | **6.12** | **0.000** | | **2.227481** | **4.741592** |
| aishn | | | **1.307138** | **.1435216** | **2.44** | **0.015** | | **1.054049** | **1.620996** |
| aisfac | | | **1.186872** | **.5889394** | **0.35** | **0.730** | | **.4487745** | **3.138917** |
| aischs | | | **1.18967** | **.1453316** | **1.42** | **0.155** | | **.9363601** | **1.511508** |
| aisabd | | | **1.99492** | **.2271002** | **6.07** | **0.000** | | **1.595972** | **2.493594** |
| aisext | | | **1.903001** | **.2507489** | **4.88** | **0.000** | | **1.469875** | **2.463756** |
| gcsmcat2  1 | | | **2.465627** | **.3375084** | **6.59** | **0.000** | | **1.88543** | **3.224367** |
| 2 | | | **1.627299** | **.2818946** | **2.81** | **0.005** | | **1.158817** | **2.285176** |
| 99 | | | **1.288767** | **.210354** | **1.55** | **0.120** | | **.9359237** | **1.774633** |
| pulse2 | | |  | | | | | | |
| 1 | | | **1.200818** | **.21063** | **1.04** | **0.297** | | **.8514752** | **1.69349** |
| 2 | | | **.8005442** | **.3982555** | **-0.45** | **0.655** | | **.3019456** | **2.122472** |
| 99 | | | **.7928869** | **.3705211** | **-0.50** | **0.619** | | **.3172776** | **1.98145** |
| bp2 1 | | | **1.269308** | **.2197958** | **1.38** | **0.168** | | **.9040056** | **1.782225** |
| 2 | | | **.2984478** | **.2111017** | **-1.71** | **0.087** | | **.0746082** | **1.193852** |
| 99 | | | **1.040269** | **.4979874** | **0.08** | **0.934** | | **.407068** | **2.658426** |
| race | | | **1.116567** | **.1476652** | **0.83** | **0.404** | | **.8616161** | **1.446956** |
| blunt_ind | | | **.6031193** | **.099615** | **-3.06** | **0.002** | | **.4363283** | **.8336679** |
| transfer | | | **1.215616** | **.202383** | **1.17** | **0.241** | | **.8771691** | **1.684649** |
| congestive_heart_failure | | | **.8483737** | **.2682904** | **-0.52** | **0.603** | | **.4564598** | **1.576783** |
| pvd | | | **1.208836** | **.5223013** | **0.44** | **0.661** | | **.5183094** | **2.819329** |
| hypertension | | | **1.570737** | **.2448075** | **2.90** | **0.004** | | **1.157282** | **2.131904** |
| dialysis | | | **1.8382** | **.6525325** | **1.71** | **0.086** | | **.9167049** | **3.686007** |
| documented_history_of_cirrhosis | | | **3.305427** | **.9500474** | **4.16** | **0.000** | | **1.881805** | **5.806044** |
| metastasis | | | **1.73838** | **1.083162** | **0.89** | **0.375** | | **.5125928** | **5.895453** |
| active_chemotherapy | | | **1** | (omitted) |  |  | |  |  |
| obesity | | | **1.13111** | **.2659624** | **0.52** | **0.600** | | **.7134419** | **1.793293** |
| ascites | | | **1.485187** | **1.688288** | **0.35** | **0.728** | | **.1600211** | **13.78431** |
| drug_use | | | **1.335087** | **.2381975** | **1.62** | **0.105** | | **.941117** | **1.89398** |
| smoker | | | **1.027234** | **.1299239** | **0.21** | **0.832** | | **.8016962** | **1.316221** |

| psych | **1.144928** | **.1679246** | **0.92** | **0.356** | **.8588825** | **1.526238** |
| --- | --- | --- | --- | --- | --- | --- |
| ac_coag | **.9273022** | **.1399371** | **-0.50** | **0.617** | **.6898717** | **1.246448** |
| routine_steroid_use | **1.010039** | **.4685454** | **0.02** | **0.983** | **.4068886** | **2.507268** |
| year_admit  2013 | **1.489794** | **.2781879** | **2.13** | **0.033** | **1.033195** | **2.148178** |
| 2014 | **1.213519** | **.3134592** | **0.75** | **0.454** | **.7314355** | **2.013339** |
| 2015 | **1.127794** | **.1798784** | **0.75** | **0.451** | **.8250238** | **1.541675** |
| 2016 | **1.080783** | **.1893659** | **0.44** | **0.657** | **.7666511** | **1.523628** |
| 2017 | **.9721085** | **.2623636** | **-0.10** | **0.917** | **.5727735** | **1.649858** |
| 2018 | **.8506176** | **.1879551** | **-0.73** | **0.464** | **.551633** | **1.311651** |
| _cons | **.0012768** | **.0003628** | **-23.45** | **0.000** | **.0007316** | **.0022283** |
| Note: **_cons** estimates baseline odds. |  |  |  |  |  |  |
| **systemic_sepsis** |  |  |  |  |  |  |
| note: ascites != 0 predicts failure | perfectly |  |  |  |  |  |

ascites dropped and 39 obs not used

Iteration 0: log pseudolikelihood = **-3353.1663**

Iteration 1: log pseudolikelihood = **-3044.6092**

Iteration 2: log pseudolikelihood = **-2927.8611**

Iteration 3: log pseudolikelihood = **-2927.0657**

Iteration 4: log pseudolikelihood = **-2927.0647**

Iteration 5: log pseudolikelihood = **-2927.0647**

| Logistic regression  Log pseudolikelihood | Number of obs Wald chi2(33) Prob > chi2  = **-2927.0647** Pseudo R2 | | | | =  =  =  = | **106,102**  **.**  **. 0.1271** | | |
| --- | --- | --- | --- | --- | --- | --- | --- | --- |
|  | |  | (Std. Err. | adjusted | | for **35** | clusters in | traumactr) |
| systemic_sepsis | | Odds Ratio | Robust Std. Err. | z | | P>\|z\| | [95% Conf. | Interval] |
| diabetes | | **1.453623** | **.1528816** | **3.56** | | **0.000** | **1.182847** | **1.786385** |
| agecat 26-45 | | **.9456561** | **.1192851** | **-0.44** | | **0.658** | **.7385206** | **1.210888** |
| 46-65 | | **1.533577** | **.1923972** | **3.41** | | **0.001** | **1.19927** | **1.961076** |
| 66-75 | | **1.850984** | **.3471572** | **3.28** | | **0.001** | **1.281615** | **2.673301** |
| >75 | | **1.60014** | **.2984557** | **2.52** | | **0.012** | **1.110179** | **2.306337** |
| male | | **1.646912** | **.1503238** | **5.47** | | **0.000** | **1.377133** | **1.96954** |
| isscat 16-24 | | **2.594415** | **.3780007** | **6.54** | | **0.000** | **1.94994** | **3.451896** |
| 25-35 | | **3.420275** | **.6507353** | **6.46** | | **0.000** | **2.355662** | **4.966027** |
| >35 | | **3.059863** | **.8154992** | **4.20** | | **0.000** | **1.814864** | **5.158934** |
| aishn | | **1.184857** | **.1259133** | **1.60** | | **0.110** | **.9620767** | **1.459224** |
| aisfac | | **1.248603** | **.4481074** | **0.62** | | **0.536** | **.6179303** | **2.522955** |
| aischs | | **1.761834** | **.2076204** | **4.81** | | **0.000** | **1.398481** | **2.219593** |
| aisabd | | **1.922775** | **.2735175** | **4.60** | | **0.000** | **1.454935** | **2.541049** |
| aisext | | **1.647478** | **.1851564** | **4.44** | | **0.000** | **1.321767** | **2.053451** |
| gcsmcat2  1 | | **2.630953** | **.2206181** | **11.54** | | **0.000** | **2.232213** | **3.100919** |
| 2 | | **2.018873** | **.3026653** | **4.69** | | **0.000** | **1.504869** | **2.708441** |
| 99 | | **1.379536** | **.3471586** | **1.28** | | **0.201** | **.8424209** | **2.259108** |
| pulse2 | |  | | | | | | |

| 1 | **1.641419** | **.1894938** | **4.29** | **0.000** | **1.309038** | **2.058194** |
| --- | --- | --- | --- | --- | --- | --- |
| 2 | **.5985313** | **.3170425** | **-0.97** | **0.333** | **.211936** | **1.69032** |
| 99 | **.4483997** | **.1612442** | **-2.23** | **0.026** | **.2216021** | **.9073121** |
| bp2 1 | **1.872032** | **.3258632** | **3.60** | **0.000** | **1.3309** | **2.633185** |
| 2 | **1.365106** | **.4012291** | **1.06** | **0.290** | **.7673311** | **2.428565** |
| 99 | **2.736867** | **.8150954** | **3.38** | **0.001** | **1.526681** | **4.906356** |
| race | **1.367829** | **.148341** | **2.89** | **0.004** | **1.105908** | **1.691782** |
| blunt_ind | **.6276339** | **.063229** | **-4.62** | **0.000** | **.5151751** | **.7646417** |
| transfer | **1.413829** | **.148326** | **3.30** | **0.001** | **1.151056** | **1.73659** |
| congestive_heart_failure | **1.985946** | **.365124** | **3.73** | **0.000** | **1.385067** | **2.847503** |
| pvd | **.3918032** | **.2471801** | **-1.49** | **0.137** | **.1137793** | **1.349188** |
| hypertension | **1.00643** | **.0988594** | **0.07** | **0.948** | **.830179** | **1.220099** |
| dialysis | **1.654049** | **.5809036** | **1.43** | **0.152** | **.8310089** | **3.292238** |
| documented_history_of_cirrhosis | **3.350921** | **1.118906** | **3.62** | **0.000** | **1.741565** | **6.447463** |
| metastasis | **1.381658** | **.7564945** | **0.59** | **0.555** | **.472438** | **4.040695** |
| active_chemotherapy | **2.587962** | **.8699439** | **2.83** | **0.005** | **1.339141** | **5.001375** |
| obesity | **1.571756** | **.2725906** | **2.61** | **0.009** | **1.118821** | **2.208053** |
| ascites | **1** | (omitted) |  |  |  |  |
| drug_use | **1.148489** | **.1258257** | **1.26** | **0.206** | **.9265548** | **1.423582** |
| smoker | **1.219839** | **.1047552** | **2.31** | **0.021** | **1.030871** | **1.443446** |
| psych | **1.422447** | **.1880974** | **2.66** | **0.008** | **1.097685** | **1.843295** |
| ac_coag | **1.243993** | **.1724892** | **1.57** | **0.115** | **.9479645** | **1.632464** |
| routine_steroid_use | **1.87485** | **.4376107** | **2.69** | **0.007** | **1.186551** | **2.962418** |
| year_admit  2013 | **.9580708** | **.1861821** | **-0.22** | **0.826** | **.6546112** | **1.402206** |
| 2014 | **.8452534** | **.2223242** | **-0.64** | **0.523** | **.5047755** | **1.415388** |
| 2015 | **.6598842** | **.1804359** | **-1.52** | **0.128** | **.3861169** | **1.12776** |
| 2016 | **.6077211** | **.1632288** | **-1.85** | **0.064** | **.358987** | **1.028798** |
| 2017 | **.7665899** | **.1576456** | **-1.29** | **0.196** | **.5122918** | **1.14712** |
| 2018 | **.5534972** | **.1824608** | **-1.79** | **0.073** | **.290079** | **1.056123** |
| _cons | **.0009641** | **.0002299** | **-29.13** | **0.000** | **.0006042** | **.0015384** |

Note: **_cons** estimates baseline odds.

# pneumonia

Iteration 0: log pseudolikelihood = **-13821.552**

Iteration 1: log pseudolikelihood = **-11875.717**

Iteration 2: log pseudolikelihood = **-11119.086**

Iteration 3: log pseudolikelihood = **-11101.481**

Iteration 4: log pseudolikelihood = **-11101.428**

Iteration 5: log pseudolikelihood = **-11101.428**

Logistic regression Number of obs = **106,141**

Wald chi2(33) = **.**

Prob > chi2 = **.**

Log pseudolikelihood = **-11101.428** Pseudo R2 = **0.1968**

(Std. Err. adjusted for **35** clusters in traumactr)

Robust pneumonia Odds Ratio Std. Err.

z

P>|z|

[95% Conf. Interval]

diabetes

**1.269113 .0680548**

**4.44 0.000**

**1.142498**

**1.409759**

agecat

| 26-45 | **1.239142** | **.0762816** | **3.48** | **0.000** | **1.0983** | **1.398045** |
| --- | --- | --- | --- | --- | --- | --- |
| 46-65 | **1.60071** | **.1590419** | **4.73** | **0.000** | **1.317467** | **1.944848** |
| 66-75 | **1.656942** | **.16157** | **5.18** | **0.000** | **1.368692** | **2.005898** |
| >75 | **1.290125** | **.1574917** | **2.09** | **0.037** | **1.015597** | **1.63886** |

| male | **1.667587** | **.0805158** | **10.59** | **0.000** | **1.517016** | **1.833103** |
| --- | --- | --- | --- | --- | --- | --- |
| isscat 16-24 | **2.788349** | **.1844533** | **15.50** | **0.000** | **2.449283** | **3.174354** |
| 25-35 | **4.389177** | **.3990307** | **16.27** | **0.000** | **3.672809** | **5.245271** |
| >35 | **4.797142** | **.6374485** | **11.80** | **0.000** | **3.69721** | **6.224307** |
| aishn | **1.655726** | **.1359328** | **6.14** | **0.000** | **1.409633** | **1.944782** |
| aisfac | **1.39938** | **.2284311** | **2.06** | **0.040** | **1.01622** | **1.927008** |
| aischs | **1.928411** | **.1133348** | **11.17** | **0.000** | **1.718595** | **2.163842** |
| aisabd | **1.277898** | **.0712214** | **4.40** | **0.000** | **1.14566** | **1.425399** |
| aisext | **1.200574** | **.0579377** | **3.79** | **0.000** | **1.092223** | **1.319674** |
| gcsmcat2  1 | **3.927953** | **.2898685** | **18.54** | **0.000** | **3.398996** | **4.539226** |
| 2 | **3.335183** | **.3809274** | **10.55** | **0.000** | **2.666243** | **4.171954** |
| 99 | **1.584169** | **.19584** | **3.72** | **0.000** | **1.243292** | **2.018505** |
| pulse2 |  |  |  |  |  |  |
| 1 | **1.418377** | **.1281937** | **3.87** | **0.000** | **1.188118** | **1.69326** |
| 2 | **.8881496** | **.1115007** | **-0.94** | **0.345** | **.6944228** | **1.135921** |
| 99 | **.4426372** | **.1232884** | **-2.93** | **0.003** | **.2564256** | **.7640721** |
| bp2 1 | **1.371681** | **.1143763** | **3.79** | **0.000** | **1.164867** | **1.615212** |
| 2 | **.6174636** | **.1139506** | **-2.61** | **0.009** | **.4300563** | **.8865383** |
| 99 | **1.15068** | **.2082566** | **0.78** | **0.438** | **.8070477** | **1.640627** |
| race | **1.056891** | **.0932903** | **0.63** | **0.531** | **.8889881** | **1.256506** |
| blunt_ind | **1.232249** | **.1245172** | **2.07** | **0.039** | **1.010847** | **1.502143** |
| transfer | **1.128389** | **.12573** | **1.08** | **0.278** | **.9070144** | **1.403794** |
| congestive_heart_failure | **1.577293** | **.1790145** | **4.02** | **0.000** | **1.262716** | **1.970241** |
| pvd | **1.277887** | **.3121826** | **1.00** | **0.316** | **.7916755** | **2.062707** |
| hypertension | **1.036634** | **.0463475** | **0.80** | **0.421** | **.9496612** | **1.131573** |
| dialysis | **.8835556** | **.1432138** | **-0.76** | **0.445** | **.643079** | **1.213957** |
| documented_history_of_cirrhosis | **1.359647** | **.1816149** | **2.30** | **0.021** | **1.04647** | **1.766548** |
| metastasis | **1.132382** | **.3473158** | **0.41** | **0.685** | **.6207526** | **2.0657** |
| active_chemotherapy | **.6141631** | **.3339982** | **-0.90** | **0.370** | **.2115328** | **1.783157** |
| obesity | **1.479817** | **.1844595** | **3.14** | **0.002** | **1.159059** | **1.889342** |
| ascites | **1.27367** | **1.031302** | **0.30** | **0.765** | **.2605141** | **6.227051** |
| drug_use | **1.02986** | **.0662157** | **0.46** | **0.647** | **.9079238** | **1.168172** |
| smoker | **1.279005** | **.0773795** | **4.07** | **0.000** | **1.13599** | **1.440024** |
| psych | **1.248462** | **.0802419** | **3.45** | **0.001** | **1.100694** | **1.416069** |
| ac_coag | **1.123658** | **.0756573** | **1.73** | **0.083** | **.9847402** | **1.282173** |
| routine_steroid_use | **1.290801** | **.2412181** | **1.37** | **0.172** | **.8949342** | **1.861776** |
| year_admit  2013 | **.874517** | **.0841606** | **-1.39** | **0.164** | **.7241882** | **1.056051** |
| 2014 | **.8375343** | **.0945061** | **-1.57** | **0.116** | **.6713582** | **1.044843** |
| 2015 | **.846832** | **.1012675** | **-1.39** | **0.164** | **.6698959** | **1.070501** |
| 2016 | **.5726167** | **.0713029** | **-4.48** | **0.000** | **.4486126** | **.7308976** |
| 2017 | **.5978935** | **.0737437** | **-4.17** | **0.000** | **.4695018** | **.7613957** |
| 2018 | **.613153** | **.0751161** | **-3.99** | **0.000** | **.4822696** | **.7795569** |
| _cons | **.0031651** | **.0005463** | **-33.34** | **0.000** | **.0022567** | **.0044393** |

Note: **_cons** estimates baseline odds.

# comp_cardiac

Iteration 0: log pseudolikelihood = **-7767.7038**

Iteration 1: log pseudolikelihood = **-7569.3173**

Iteration 2: log pseudolikelihood = **-6387.7653**

Iteration 3: log pseudolikelihood = **-6341.7745**

Iteration 4: log pseudolikelihood = **-6341.0498**

Iteration 5: log pseudolikelihood = **-6341.0493**

| Logistic regression  Log pseudolikelihood | Number of obs Wald chi2(33) Prob > chi2  = **-6341.0493** Pseudo R2 | | | | =  =  =  = | **106,141**  **.**  **. 0.1837** | | |
| --- | --- | --- | --- | --- | --- | --- | --- | --- |
| (Std. Err. adjusted for **35** clusters in traumactr) | | | | | | | | |
| comp_cardiac | | Odds Ratio | Robust Std. Err. | z | P>\|z\| | | [95% Conf. | Interval] |
| diabetes | | **1.401236** | **.1155435** | **4.09** | **0.000** | | **1.192127** | **1.647024** |
| agecat 26-45 | | **1.286336** | **.1034879** | **3.13** | **0.002** | | **1.098686** | **1.506035** |
| 46-65 | | **2.241115** | **.2148855** | **8.42** | **0.000** | | **1.857155** | **2.704457** |
| 66-75 | | **2.858193** | **.3968049** | **7.56** | **0.000** | | **2.177302** | **3.752014** |
| >75 | | **3.75338** | **.4056953** | **12.24** | **0.000** | | **3.036812** | **4.639031** |
| male | | **1.265014** | **.0729083** | **4.08** | **0.000** | | **1.129892** | **1.416296** |
| isscat 16-24 | | **2.619256** | **.2263054** | **11.14** | **0.000** | | **2.211228** | **3.102575** |
| 25-35 | | **4.146498** | **.3907382** | **15.09** | **0.000** | | **3.447227** | **4.987616** |
| >35 | | **7.72936** | **1.066533** | **14.82** | **0.000** | | **5.897811** | **10.12969** |
| aishn | | **.7719359** | **.0842852** | **-2.37** | **0.018** | | **.6232198** | **.9561393** |
| aisfac | | **.840966** | **.2680834** | **-0.54** | **0.587** | | **.4502276** | **1.570814** |
| aischs | | **1.26094** | **.1118387** | **2.61** | **0.009** | | **1.059736** | **1.500347** |
| aisabd | | **1.305211** | **.1212983** | **2.87** | **0.004** | | **1.087866** | **1.56598** |
| aisext | | **1.735047** | **.1130783** | **8.45** | **0.000** | | **1.526989** | **1.971454** |
| gcsmcat2  1 | | **2.686108** | **.2912724** | **9.11** | **0.000** | | **2.171811** | **3.322193** |
| 2 | | **4.892588** | **.571912** | **13.58** | **0.000** | | **3.890798** | **6.152316** |
| 99 | | **1.524277** | **.176457** | **3.64** | **0.000** | | **1.214857** | **1.912506** |
| pulse2 | |  | | | | | | |
| 1 | | **1.782674** | **.1432** | **7.20** | **0.000** | | **1.522986** | **2.086642** |
| 2 | | **1.832633** | **.2791476** | **3.98** | **0.000** | | **1.359627** | **2.470193** |
| 99 | | **.748806** | **.1450841** | **-1.49** | **0.135** | | **.5122071** | **1.094695** |
| bp2 1 | | **1.934914** | **.20818** | **6.13** | **0.000** | | **1.567039** | **2.389151** |
| 2 | | **2.372909** | **.5036784** | **4.07** | **0.000** | | **1.56532** | **3.597152** |
| 99 | | **1.772576** | **.3728735** | **2.72** | **0.007** | | **1.173677** | **2.677077** |
| race | | **1.299989** | **.0960941** | **3.55** | **0.000** | | **1.124655** | **1.502656** |
| blunt_ind | | **.4311509** | **.0461189** | **-7.87** | **0.000** | | **.349606** | **.531716** |
| transfer | | **.8622713** | **.0805298** | **-1.59** | **0.113** | | **.7180389** | **1.035476** |
| congestive_heart_failure | | **1.382748** | **.1571169** | **2.85** | **0.004** | | **1.106684** | **1.727675** |
| pvd | | **1.655609** | **.2804448** | **2.98** | **0.003** | | **1.187879** | **2.307509** |
| hypertension | | **1.123736** | **.0833752** | **1.57** | **0.116** | | **.9716499** | **1.299628** |
| dialysis | | **2.47113** | **.519243** | **4.31** | **0.000** | | **1.63696** | **3.730381** |
| documented_history_of_cirrhosis | | **2.694904** | **.476925** | **5.60** | **0.000** | | **1.905039** | **3.812264** |
| metastasis | | **.3984063** | **.2156193** | **-1.70** | **0.089** | | **.1379279** | **1.150801** |
| active_chemotherapy | | **.6828489** | **.3293588** | **-0.79** | **0.429** | | **.2653165** | **1.757458** |
| obesity | | **1.125014** | **.1113153** | **1.19** | **0.234** | | **.9266916** | **1.36578** |
| ascites | | **.2966898** | **.3622794** | **-1.00** | **0.320** | | **.0270972** | **3.24848** |
| drug_use | | **.8732739** | **.0603219** | **-1.96** | **0.050** | | **.7626992** | **.9998796** |
| smoker | | **.7117203** | **.0590838** | **-4.10** | **0.000** | | **.6048484** | **.8374757** |

| psych | **.8296126** | **.0816907** | **-1.90** | **0.058** | **.6840043** | **1.006218** |
| --- | --- | --- | --- | --- | --- | --- |
| ac_coag | **1.352054** | **.0990234** | **4.12** | **0.000** | **1.171258** | **1.560757** |
| routine_steroid_use | **1.768065** | **.3197887** | **3.15** | **0.002** | **1.240344** | **2.520313** |
| year_admit  2013 | **.9213859** | **.1381757** | **-0.55** | **0.585** | **.6867379** | **1.236209** |
| 2014 | **.9750602** | **.1208322** | **-0.20** | **0.839** | **.7648005** | **1.243125** |
| 2015 | **1.304041** | **.1856171** | **1.87** | **0.062** | **.9865781** | **1.723659** |
| 2016 | **1.112579** | **.1952587** | **0.61** | **0.543** | **.7887586** | **1.569342** |
| 2017 | **1.100928** | **.1763136** | **0.60** | **0.548** | **.8043385** | **1.50688** |
| 2018 | **1.284347** | **.2320609** | **1.39** | **0.166** | **.9013305** | **1.830126** |
| _cons | **.0026874** | **.0006244** | **-25.48** | **0.000** | **.0017043** | **.0042374** |

Note: **_cons** estimates baseline odds.

# acute_renal_failure

note: aisfac != 0 predicts failure perfectly aisfac dropped and 544 obs not used

Iteration 0: log pseudolikelihood = **-3318.8801**

Iteration 1: log pseudolikelihood = **-3020.1331**

Iteration 2: log pseudolikelihood = **-2830.3189**

Iteration 3: log pseudolikelihood = **-2825.7782**

Iteration 4: log pseudolikelihood = **-2825.7632**

Iteration 5: log pseudolikelihood = **-2825.7632**

| Logistic regression  Log pseudolikelihood | Number of obs Wald chi2(33) Prob > chi2  = **-2825.7632** Pseudo R2 | | | | =  =  =  = | **105,597**  **.**  **. 0.1486** | | |
| --- | --- | --- | --- | --- | --- | --- | --- | --- |
|  | |  | (Std. Err. | adjusted | | for **35** | clusters in | traumactr) |
| acute_renal_failure | | Odds Ratio | Robust Std. Err. | z | | P>\|z\| | [95% Conf. | Interval] |
| diabetes | | **1.60779** | **.1947855** | **3.92** | | **0.000** | **1.26796** | **2.0387** |
| agecat 26-45 | | **1.998225** | **.4102605** | **3.37** | | **0.001** | **1.336232** | **2.988179** |
| 46-65 | | **2.691773** | **.5798118** | **4.60** | | **0.000** | **1.764771** | **4.105712** |
| 66-75 | | **4.5597** | **1.11456** | **6.21** | | **0.000** | **2.824041** | **7.362097** |
| >75 | | **3.940253** | **.9912737** | **5.45** | | **0.000** | **2.406479** | **6.451581** |
| male | | **1.872953** | **.1837637** | **6.40** | | **0.000** | **1.545296** | **2.270084** |
| isscat 16-24 | | **2.566671** | **.3361285** | **7.20** | | **0.000** | **1.985627** | **3.317742** |
| 25-35 | | **4.490056** | **.6674338** | **10.10** | | **0.000** | **3.355237** | **6.008699** |
| >35 | | **5.02012** | **1.187259** | **6.82** | | **0.000** | **3.157947** | **7.980375** |
| aishn | | **.6404182** | **.1014719** | **-2.81** | | **0.005** | **.469455** | **.8736417** |
| aisfac | | **1** | (omitted) |  | |  |  |  |
| aischs | | **1.568316** | **.2046314** | **3.45** | | **0.001** | **1.214423** | **2.025336** |
| aisabd | | **2.294563** | **.3432794** | **5.55** | | **0.000** | **1.711415** | **3.076411** |
| aisext | | **2.143507** | **.2643381** | **6.18** | | **0.000** | **1.683273** | **2.729578** |
| gcsmcat2  1 | | **2.246474** | **.31554** | **5.76** | | **0.000** | **1.705853** | **2.95843** |
| 2 | | **1.793471** | **.2904149** | **3.61** | | **0.000** | **1.30575** | **2.463364** |
| 99 | | **1.146416** | **.2828178** | **0.55** | | **0.580** | **.7068919** | **1.859223** |
| pulse2 | |  | | | | | | |

| 1 | **2.180779** | **.2872412** | **5.92** | **0.000** | **1.684595** | **2.823109** |
| --- | --- | --- | --- | --- | --- | --- |
| 2 | **1.457253** | **.4384501** | **1.25** | **0.211** | **.808035** | **2.628087** |
| 99 | **.5296415** | **.2163279** | **-1.56** | **0.120** | **.2378568** | **1.179366** |
| bp2 1 | **1.363074** | **.2581243** | **1.64** | **0.102** | **.9404331** | **1.975653** |
| 2 | **1.02856** | **.3852043** | **0.08** | **0.940** | **.4936821** | **2.142948** |
| 99 | **1.589367** | **.4885383** | **1.51** | **0.132** | **.8701269** | **2.903125** |
| race | **1.435841** | **.1526319** | **3.40** | **0.001** | **1.165796** | **1.76844** |
| blunt_ind | **.5212759** | **.0823249** | **-4.13** | **0.000** | **.3825056** | **.7103912** |
| transfer | **1.320784** | **.1505182** | **2.44** | **0.015** | **1.056398** | **1.651337** |
| congestive_heart_failure | **2.032817** | **.6410245** | **2.25** | **0.024** | **1.095676** | **3.771503** |
| pvd | **1.148606** | **.4535167** | **0.35** | **0.726** | **.5297633** | **2.49035** |
| hypertension | **1.280122** | **.1794301** | **1.76** | **0.078** | **.9726158** | **1.684849** |
| dialysis | **1.499711** | **.3409695** | **1.78** | **0.075** | **.960464** | **2.341715** |
| documented_history_of_cirrhosis | **4.275968** | **1.227763** | **5.06** | **0.000** | **2.435724** | **7.506558** |
| metastasis | **1.211877** | **.7941839** | **0.29** | **0.769** | **.3354573** | **4.378039** |
| active_chemotherapy | **1.47567** | **.7716268** | **0.74** | **0.457** | **.5295376** | **4.112273** |
| obesity | **1.714285** | **.2290724** | **4.03** | **0.000** | **1.319291** | **2.227539** |
| ascites | **.6340683** | **.8946181** | **-0.32** | **0.747** | **.0399173** | **10.0719** |
| drug_use | **.6827591** | **.1165628** | **-2.24** | **0.025** | **.4885935** | **.9540855** |
| smoker | **.9633148** | **.1127996** | **-0.32** | **0.750** | **.7657669** | **1.211825** |
| psych | **.9244355** | **.1515356** | **-0.48** | **0.632** | **.670418** | **1.274699** |
| ac_coag | **1.088484** | **.1254669** | **0.74** | **0.462** | **.8683729** | **1.364389** |
| routine_steroid_use | **1.513059** | **.4435848** | **1.41** | **0.158** | **.8517424** | **2.687839** |
| year_admit  2013 | **.7568071** | **.1462702** | **-1.44** | **0.149** | **.5181686** | **1.105349** |
| 2014 | **.7748825** | **.212001** | **-0.93** | **0.351** | **.4532675** | **1.324699** |
| 2015 | **.9487187** | **.3328595** | **-0.15** | **0.881** | **.4769711** | **1.887048** |
| 2016 | **1.064671** | **.3562927** | **0.19** | **0.851** | **.5525355** | **2.051495** |
| 2017 | **.8843754** | **.2740412** | **-0.40** | **0.692** | **.4818091** | **1.623298** |
| 2018 | **.7108489** | **.242164** | **-1.00** | **0.316** | **.3645856** | **1.385974** |
| _cons | **.0005177** | **.0002141** | **-18.29** | **0.000** | **.0002302** | **.0011645** |
| Note: **_cons** estimates baseline odds. |  |  |  |  |  |  |
| **comp_vte** |  |  |  |  |  |  |
| note: ascites != 0 predicts failure | perfectly |  |  |  |  |  |

ascites dropped and 39 obs not used

Iteration 0: log pseudolikelihood = **-7276.6524**

Iteration 1: log pseudolikelihood = **-6631.7889**

Iteration 2: log pseudolikelihood = **-6429.7711**

Iteration 3: log pseudolikelihood = **-6428.7931**

Iteration 4: log pseudolikelihood = **-6428.7923**

Iteration 5: log pseudolikelihood = **-6428.7923**

| Logistic regression |  | Number of obs | = | **106,102** |
| --- | --- | --- | --- | --- |
|  |  | Wald chi2(33) | = | **.** |
|  |  | Prob > chi2 | = | **.** |
| Log pseudolikelihood | = **-6428.7923** | Pseudo R2 | = | **0.1165** |

(Std. Err. adjusted for **35** clusters in traumactr)

| comp_vte | Odds Ratio | Robust Std. Err. | z | P>\|z\| | [95% Conf. | Interval] |
| --- | --- | --- | --- | --- | --- | --- |
| diabetes | **1.146404** | **.0852572** | **1.84** | **0.066** | **.9909104** | **1.326297** |
| agecat 26-45 | **1.433236** | **.1326619** | **3.89** | **0.000** | **1.195445** | **1.718327** |
| 46-65 | **2.038017** | **.2120534** | **6.84** | **0.000** | **1.66204** | **2.499047** |
| 66-75 | **2.1476** | **.2562853** | **6.41** | **0.000** | **1.699709** | **2.713514** |
| >75 | **1.433537** | **.1880717** | **2.75** | **0.006** | **1.108501** | **1.853879** |
| male | **1.340266** | **.0807752** | **4.86** | **0.000** | **1.190942** | **1.508312** |
| isscat 16-24 | **2.625492** | **.1519175** | **16.68** | **0.000** | **2.344002** | **2.940785** |
| 25-35 | **3.539994** | **.3471257** | **12.89** | **0.000** | **2.921025** | **4.290125** |
| >35 | **4.130576** | **.6081191** | **9.63** | **0.000** | **3.095234** | **5.512235** |
| aishn | **1.320016** | **.1284346** | **2.85** | **0.004** | **1.090835** | **1.597347** |
| aisfac | **1.480142** | **.2457707** | **2.36** | **0.018** | **1.06897** | **2.04947** |
| aischs | **1.310509** | **.0786837** | **4.50** | **0.000** | **1.16502** | **1.474167** |
| aisabd | **1.235458** | **.1221628** | **2.14** | **0.032** | **1.017796** | **1.499669** |
| aisext | **2.549465** | **.1952465** | **12.22** | **0.000** | **2.194124** | **2.962354** |
| gcsmcat2  1 | **1.997445** | **.1727924** | **8.00** | **0.000** | **1.685933** | **2.366517** |
| 2 | **1.627929** | **.1807763** | **4.39** | **0.000** | **1.309521** | **2.023759** |
| 99 | **1.146255** | **.2139933** | **0.73** | **0.465** | **.7950074** | **1.652689** |
| pulse2 |  | | | | | |
| 1 | **1.736596** | **.1094003** | **8.76** | **0.000** | **1.534885** | **1.964816** |
| 2 | **.9933354** | **.1913937** | **-0.03** | **0.972** | **.6809077** | **1.449117** |
| 99 | **.7283625** | **.2534993** | **-0.91** | **0.362** | **.3682098** | **1.440787** |
| bp2 1 | **1.561736** | **.1459687** | **4.77** | **0.000** | **1.300318** | **1.87571** |
| 2 | **.9817765** | **.2408104** | **-0.07** | **0.940** | **.6070573** | **1.587799** |
| 99 | **1.04334** | **.3185951** | **0.14** | **0.889** | **.573459** | **1.898233** |
| race | **1.176952** | **.083911** | **2.29** | **0.022** | **1.023463** | **1.35346** |
| blunt_ind | **.6767551** | **.0651612** | **-4.06** | **0.000** | **.5603685** | **.8173147** |
| transfer | **1.377577** | **.1371728** | **3.22** | **0.001** | **1.133332** | **1.67446** |
| congestive_heart_failure | **.7697959** | **.1439485** | **-1.40** | **0.162** | **.5335862** | **1.110572** |
| pvd | **1.291053** | **.3432245** | **0.96** | **0.337** | **.7667498** | **2.173876** |
| hypertension | **.9191615** | **.0793531** | **-0.98** | **0.329** | **.7760789** | **1.088624** |
| dialysis | **.4761001** | **.2227943** | **-1.59** | **0.113** | **.190271** | **1.191308** |
| documented_history_of_cirrhosis | **1.107327** | **.2892938** | **0.39** | **0.696** | **.6635844** | **1.847804** |
| metastasis | **1.236848** | **.4924614** | **0.53** | **0.593** | **.5667654** | **2.699165** |
| active_chemotherapy | **1.12729** | **.6287404** | **0.21** | **0.830** | **.3778175** | **3.363482** |
| obesity | **1.842085** | **.2557773** | **4.40** | **0.000** | **1.403197** | **2.418248** |
| ascites | **1** | (omitted) |  |  |  |  |
| drug_use | **1.106976** | **.0788356** | **1.43** | **0.154** | **.9627604** | **1.272795** |
| smoker | **.8546107** | **.0604152** | **-2.22** | **0.026** | **.7440363** | **.9816179** |
| psych | **.9438319** | **.0741191** | **-0.74** | **0.462** | **.8091887** | **1.100879** |
| ac_coag | **1.128372** | **.1162687** | **1.17** | **0.241** | **.9220269** | **1.380896** |
| routine_steroid_use | **.7755017** | **.170076** | **-1.16** | **0.246** | **.5045509** | **1.191957** |
| year_admit  2013 | **.855655** | **.1147471** | **-1.16** | **0.245** | **.6578833** | **1.112881** |
| 2014 | **.8042023** | **.0948829** | **-1.85** | **0.065** | **.6381713** | **1.013429** |
| 2015 | **.8971179** | **.1458209** | **-0.67** | **0.504** | **.6523671** | **1.233693** |
| 2016 | **.9618751** | **.0916556** | **-0.41** | **0.683** | **.7980112** | **1.159387** |
| 2017 | **.9055287** | **.1135835** | **-0.79** | **0.429** | **.708163** | **1.157901** |
| 2018 | **.869057** | **.1235346** | **-0.99** | **0.323** | **.6577362** | **1.148272** |

_cons

**.0030145 .0004412 -39.66 0.000**

**.0022627**

**.0040161**

Note: **_cons** estimates baseline odds.

68 .

69 . **Table 4 Data: Outcomes for diabetic vs non-diabetic patients, stratified by ISS category. 70 .

1. . **Sample size
2. . tab isscat

| isscat | Freq. | Percent | Cum. |
| --- | --- | --- | --- |
| 5-15 | **80,289** | **75.64** | **75.64** |
| 16-24 | **16,097** | **15.17** | **90.81** |
| 25-35 | **7,742** | **7.29** | **98.10** |
| >35 | **2,013** | **1.90** | **100.00** |
| Total | **106,141** | **100.00** |  |

1. . tab isscat, nolab

| isscat | Freq. | Percent | Cum. |
| --- | --- | --- | --- |
| 0 | **80,289** | **75.64** | **75.64** |
| 1 | **16,097** | **15.17** | **90.81** |
| 2 | **7,742** | **7.29** | **98.10** |
| 3 | **2,013** | **1.90** | **100.00** |
| Total | **106,141** | **100.00** |  |

74 .

1. .
2. .
3. .
4. .
5. .
6. .
7. .
8. .

83 .

84 .

85 .

86 . **For ISS 5-15

87 . outcomes_iss 0

(25,852 observations deleted)

| isscat | Freq. | Percent | Cum. |
| --- | --- | --- | --- |
| 5-15 | **80,289** | **100.00** | **100.00** |
| Total | **80,289** | **100.00** |  |

# dead

Iteration 0: log pseudolikelihood = **-5909.9028**

Iteration 1: log pseudolikelihood = **-5138.532**

Iteration 2: log pseudolikelihood = **-4684.7198**

Iteration 3: log pseudolikelihood = **-4598.904**

Iteration 4: log pseudolikelihood = **-4597.1709**

Iteration 5: log pseudolikelihood = **-4597.1671**

Iteration 6: log pseudolikelihood = **-4597.1671**

Logistic regression Number of obs = **80,289**

Wald chi2(33) = **.**

Prob > chi2 = **.**

Log pseudolikelihood = **-4597.1671** Pseudo R2 = **0.2221**

(Std. Err. adjusted for **35** clusters in traumactr)

| dead diabetes agecat | Robust  Odds Ratio Std. Err. z P>\|z\| [95% Conf. Interval]  **1.251675 .1072067 2.62 0.009 1.058243 1.480462** | | | | | |
| --- | --- | --- | --- | --- | --- | --- |
| 26-45 | **.9205329** | **.186095** | **-0.41** | **0.682** | **.6193846** | **1.368101** |
| 46-65 | **2.262379** | **.3318982** | **5.57** | **0.000** | **1.697037** | **3.016056** |
| 66-75 | **5.473958** | **.9099275** | **10.23** | **0.000** | **3.951915** | **7.582202** |
| >75 | **11.97838** | **1.492667** | **19.93** | **0.000** | **9.382677** | **15.29217** |
| male | **1.361046** | **.1209688** | **3.47** | **0.001** | **1.143453** | **1.620044** |
| aishn | **2.061363** | **.1439829** | **10.36** | **0.000** | **1.797626** | **2.363794** |
| aisfac | **1.470382** | **1.184099** | **0.48** | **0.632** | **.3033591** | **7.126941** |
| aischs | **1.581242** | **.1538411** | **4.71** | **0.000** | **1.306724** | **1.913431** |
| aisabd | **2.135927** | **.2917167** | **5.56** | **0.000** | **1.634303** | **2.791517** |
| aisext | **1.461718** | **.1457156** | **3.81** | **0.000** | **1.202289** | **1.777125** |
| gcsmcat2  1 | **4.20376** | **.460259** | **13.12** | **0.000** | **3.391892** | **5.209953** |
| 2 | **40.5463** | **5.291518** | **28.37** | **0.000** | **31.3953** | **52.3646** |
| 99 | **.9408779** | **.1181126** | **-0.49** | **0.627** | **.7356617** | **1.20334** |
| pulse2 |  | | | | | |
| 1 | **2.694935** | **.3423528** | **7.80** | **0.000** | **2.100948** | **3.456855** |
| 2 | **1.473743** | **.4208131** | **1.36** | **0.174** | **.8421108** | **2.579135** |
| 99 | **1.690532** | **.4272876** | **2.08** | **0.038** | **1.0301** | **2.77439** |

| bp2 1 | **3.513241** | **.4230917** | **10.43** | **0.000** | **2.774595** | **4.448527** |
| --- | --- | --- | --- | --- | --- | --- |
| 2 | **5.316586** | **1.611024** | **5.51** | **0.000** | **2.935641** | **9.628591** |
| 99 | **1.861696** | **.5971607** | **1.94** | **0.053** | **.9928321** | **3.490933** |
| race | **.8847458** | **.1015758** | **-1.07** | **0.286** | **.7064701** | **1.108009** |
| blunt_ind | **.3157296** | **.0469732** | **-7.75** | **0.000** | **.235872** | **.422624** |
| transfer | **.9294455** | **.0924416** | **-0.74** | **0.462** | **.764829** | **1.129493** |
| congestive_heart_failure | **2.190973** | **.2522106** | **6.81** | **0.000** | **1.748446** | **2.745502** |
| pvd | **1.824699** | **.3578002** | **3.07** | **0.002** | **1.242457** | **2.679792** |
| hypertension | **.9284078** | **.0589842** | **-1.17** | **0.242** | **.819709** | **1.051521** |
| dialysis | **2.873296** | **.5292669** | **5.73** | **0.000** | **2.002568** | **4.122621** |
| documented_history_of_cirrhosis | **5.387259** | **1.472883** | **6.16** | **0.000** | **3.152451** | **9.206346** |
| metastasis | **2.763355** | **.7617635** | **3.69** | **0.000** | **1.609864** | **4.743339** |
| active_chemotherapy | **1.409712** | **.5681784** | **0.85** | **0.394** | **.6398192** | **3.106015** |
| obesity | **.9276666** | **.1357532** | **-0.51** | **0.608** | **.6963515** | **1.23582** |
| ascites | **2.325291** | **1.497001** | **1.31** | **0.190** | **.6583876** | **8.212455** |
| drug_use | **.4672548** | **.1082523** | **-3.28** | **0.001** | **.296722** | **.7357966** |
| smoker | **.6471052** | **.0723837** | **-3.89** | **0.000** | **.5197105** | **.8057277** |
| psych | **1.035523** | **.0802126** | **0.45** | **0.652** | **.8896611** | **1.205298** |
| ac_coag | **1.299252** | **.1511937** | **2.25** | **0.024** | **1.034282** | **1.632103** |
| routine_steroid_use | **1.764629** | **.3359872** | **2.98** | **0.003** | **1.215021** | **2.562849** |
| year_admit  2013 | **1.134288** | **.2348826** | **0.61** | **0.543** | **.755894** | **1.702103** |
| 2014 | **1.164298** | **.2237698** | **0.79** | **0.429** | **.7988575** | **1.696911** |
| 2015 | **1.297269** | **.2473509** | **1.36** | **0.172** | **.8927524** | **1.885077** |
| 2016 | **1.430887** | **.2368799** | **2.16** | **0.030** | **1.034405** | **1.979336** |
| 2017 | **1.200667** | **.1792073** | **1.23** | **0.220** | **.8961386** | **1.608681** |
| 2018 | **.8463576** | **.1451646** | **-0.97** | **0.331** | **.6047258** | **1.184539** |
| _cons | **.0026444** | **.0007108** | **-22.08** | **0.000** | **.0015615** | **.0044786** |

Note: **_cons** estimates baseline odds.

(25,852 observations deleted)

| isscat | Freq. | Percent | Cum. |
| --- | --- | --- | --- |
| 5-15 | **80,289** | **100.00** | **100.00** |
| Total | **80,289** | **100.00** |  |

| **comp_any**  Iteration 0: log pseudolikelihood | = | **-13552.131** |  | | |
| --- | --- | --- | --- | --- | --- |
| Iteration 1: log pseudolikelihood | = | **-13335.279** |  |  |  |
| Iteration 2: log pseudolikelihood | = | **-12507.095** |  |  |  |
| Iteration 3: log pseudolikelihood | = | **-12490.039** |  |  |  |
| Iteration 4: log pseudolikelihood | = | **-12484.29** |  |  |  |
| Iteration 5: log pseudolikelihood | = | **-12484.268** |  |  |  |
| Iteration 6: log pseudolikelihood | = | **-12484.268** |  |  |  |
| Logistic regression  Log pseudolikelihood = **-12484.268** |  |  | Number of obs Wald chi2(33) Prob > chi2 Pseudo R2 | =  =  =  = | **80,289**  **.**  **. 0.0788** |

(Std. Err. adjusted for **35** clusters in traumactr)

| comp_any | Odds Ratio | | Robust Std. Err. | z | P>\|z\| | [95% Conf. | Interval] |
| --- | --- | --- | --- | --- | --- | --- | --- |
| diabetes | **1.369924** | | **.0565472** | **7.63** | **0.000** | **1.263458** | **1.485361** |
| agecat 26-45 | **1.073107** | | **.094055** | **0.81** | **0.421** | **.903727** | **1.274232** |
| 46-65 | **1.975853** | | **.2363563** | **5.69** | **0.000** | **1.562902** | **2.497913** |
| 66-75 | **2.637637** | | **.3522638** | **7.26** | **0.000** | **2.03018** | **3.426852** |
| >75 | **2.664069** | | **.3521096** | **7.41** | **0.000** | **2.05609** | **3.451824** |
| male | **1.322592** | | **.0635275** | **5.82** | **0.000** | **1.203762** | **1.453153** |
| aishn | **1.842245** | | **.1161972** | **9.69** | **0.000** | **1.628017** | **2.084663** |
| aisfac | **3.35568** | | **1.017349** | **3.99** | **0.000** | **1.852337** | **6.079126** |
| aischs | **1.974097** | | **.1480476** | **9.07** | **0.000** | **1.704247** | **2.286675** |
| aisabd | **3.21394** | | **.5333295** | **7.04** | **0.000** | **2.321598** | **4.449265** |
| aisext | **2.429534** | | **.1287109** | **16.76** | **0.000** | **2.189921** | **2.695365** |
| gcsmcat2  1 | **3.285872** | | **.2761358** | **14.16** | **0.000** | **2.786878** | **3.874211** |
| 2 | **6.440939** | | **.7791652** | **15.40** | **0.000** | **5.081346** | **8.164314** |
| 99 | **1.051828** | | **.0912546** | **0.58** | **0.560** | **.8873521** | **1.24679** |
| pulse2 |  | | | | | | |
| 1 | | **2.010962** | **.1559139** | **9.01** | **0.000** | **1.727462** | **2.340988** |
| 2 | | **1.328942** | **.2127017** | **1.78** | **0.076** | **.9711093** | **1.818627** |
| 99 | | **.802713** | **.1237106** | **-1.43** | **0.154** | **.5934401** | **1.085785** |
| bp2 1 | | **1.945769** | **.2042789** | **6.34** | **0.000** | **1.583897** | **2.390318** |
| 2 | | **2.078889** | **.2982874** | **5.10** | **0.000** | **1.569269** | **2.754007** |
| 99 | | **1.334205** | **.267677** | **1.44** | **0.151** | **.9004279** | **1.976953** |
| race | | **1.01338** | **.0664927** | **0.20** | **0.839** | **.8910886** | **1.152454** |
| blunt_ind | | **.6429352** | **.0667178** | **-4.26** | **0.000** | **.5246111** | **.7879468** |
| transfer | | **1.238987** | **.1073889** | **2.47** | **0.013** | **1.045416** | **1.4684** |
| congestive_heart_failure | | **1.661951** | **.1514233** | **5.58** | **0.000** | **1.390157** | **1.986885** |
| pvd | | **1.109373** | **.1305587** | **0.88** | **0.378** | **.8808504** | **1.397181** |
| hypertension | | **1.119595** | **.0458832** | **2.76** | **0.006** | **1.033182** | **1.213235** |
| dialysis | | **1.740726** | **.220721** | **4.37** | **0.000** | **1.357686** | **2.231831** |
| documented_history_of_cirrhosis | | **2.263582** | **.4102385** | **4.51** | **0.000** | **1.586826** | **3.228963** |
| metastasis | | **1.411227** | **.2769712** | **1.76** | **0.079** | **.9605895** | **2.073271** |
| active_chemotherapy | | **1.160371** | **.2747412** | **0.63** | **0.530** | **.7295547** | **1.845594** |
| obesity | | **1.15962** | **.0855341** | **2.01** | **0.045** | **1.003531** | **1.339987** |
| ascites | | **2.376262** | **1.192674** | **1.72** | **0.085** | **.8885182** | **6.355099** |
| drug_use | | **1.174022** | **.0932535** | **2.02** | **0.043** | **1.004766** | **1.371791** |
| smoker | | **1.087602** | **.0753417** | **1.21** | **0.225** | **.9495208** | **1.245763** |
| psych | | **1.245819** | **.0496296** | **5.52** | **0.000** | **1.152248** | **1.34699** |
| ac_coag | | **1.286372** | **.0798489** | **4.06** | **0.000** | **1.139017** | **1.452792** |
| routine_steroid_use | | **1.460337** | **.1531938** | **3.61** | **0.000** | **1.188939** | **1.793687** |
| year_admit  2013 | | **.9063151** | **.0672938** | **-1.32** | **0.185** | **.7835696** | **1.048288** |
| 2014 | | **.8996048** | **.0725166** | **-1.31** | **0.189** | **.7681339** | **1.053578** |
| 2015 | | **.818254** | **.0812823** | **-2.02** | **0.043** | **.6734928** | **.9941303** |
| 2016 | | **.7112244** | **.0773474** | **-3.13** | **0.002** | **.5746935** | **.880191** |
| 2017 | | **.6432696** | **.0808368** | **-3.51** | **0.000** | **.5028359** | **.8229241** |
| 2018 | | **.5258457** | **.0628255** | **-5.38** | **0.000** | **.4160647** | **.6645929** |
| _cons | | **.0136596** | **.0024963** | **-23.49** | **0.000** | **.0095474** | **.0195432** |
| Note: **_cons** estimates baseline odds. | |  |  |  |  |  |  |

(25,852 observations deleted)

| isscat | Freq. | Percent | Cum. |
| --- | --- | --- | --- |
| 5-15 | **80,289** | **100.00** | **100.00** |
| Total | **80,289** | **100.00** |  |

# comp_sev

Iteration 0: log pseudolikelihood = **-11445.164**

Iteration 1: log pseudolikelihood = **-10698.054**

Iteration 2: log pseudolikelihood = **-10486.589**

Iteration 3: log pseudolikelihood = **-10481.093**

Iteration 4: log pseudolikelihood = **-10481.076**

Iteration 5: log pseudolikelihood = **-10481.076**

Logistic regression Number of obs = **80,289**

Wald chi2(33) = **.**

Prob > chi2 = **.**

Log pseudolikelihood = **-10481.076** Pseudo R2 = **0.0842**

(Std. Err. adjusted for **35** clusters in traumactr)

| comp_sev diabetes agecat | Robust  Odds Ratio Std. Err. z P>\|z\| [95% Conf. Interval]  **1.379837 .0542313 8.19 0.000 1.277537 1.49033** | | | | | |
| --- | --- | --- | --- | --- | --- | --- |
| 26-45 | **1.119389** | **.127334** | **0.99** | **0.321** | **.8956824** | **1.398968** |
| 46-65 | **2.129917** | **.2914148** | **5.53** | **0.000** | **1.628926** | **2.784992** |
| 66-75 | **2.915393** | **.4658063** | **6.70** | **0.000** | **2.131555** | **3.987472** |
| >75 | **2.809458** | **.4126352** | **7.03** | **0.000** | **2.106704** | **3.746636** |
| male | **1.599539** | **.0692102** | **10.86** | **0.000** | **1.469482** | **1.741107** |
| aishn | **1.948977** | **.1348758** | **9.64** | **0.000** | **1.70177** | **2.232096** |
| aisfac | **3.906854** | **1.318755** | **4.04** | **0.000** | **2.016067** | **7.570935** |
| aischs | **2.062851** | **.1624223** | **9.20** | **0.000** | **1.767856** | **2.40707** |
| aisabd | **2.909386** | **.5141669** | **6.04** | **0.000** | **2.057648** | **4.11369** |
| aisext | **2.590474** | **.1429125** | **17.25** | **0.000** | **2.324983** | **2.886281** |
| gcsmcat2  1 | **3.442803** | **.3228721** | **13.18** | **0.000** | **2.864738** | **4.137513** |
| 2 | **7.659907** | **.9898906** | **15.75** | **0.000** | **5.945969** | **9.867892** |
| 99 | **1.108571** | **.0912117** | **1.25** | **0.210** | **.9434693** | **1.302564** |
| pulse2 |  | | | | | |
| 1 | **2.153262** | **.1800299** | **9.17** | **0.000** | **1.827804** | **2.536671** |
| 2 | **1.351454** | **.1944329** | **2.09** | **0.036** | **1.019387** | **1.791691** |
| 99 | **.7357921** | **.105181** | **-2.15** | **0.032** | **.5560021** | **.9737193** |
| bp2 1 | **1.95537** | **.2287988** | **5.73** | **0.000** | **1.554639** | **2.459396** |
| 2 | **2.430434** | **.3862936** | **5.59** | **0.000** | **1.779893** | **3.318746** |
| 99 | **1.533381** | **.3255431** | **2.01** | **0.044** | **1.011432** | **2.324683** |
| race | **1.032843** | **.0701085** | **0.48** | **0.634** | **.9041816** | **1.179813** |
| blunt_ind | **.7820054** | **.0815657** | **-2.36** | **0.018** | **.6374214** | **.9593849** |
| transfer | **1.185901** | **.103095** | **1.96** | **0.050** | **1.000115** | **1.406198** |
| congestive_heart_failure | **1.697456** | **.1607775** | **5.59** | **0.000** | **1.409859** | **2.043721** |
| pvd | **1.034418** | **.1426603** | **0.25** | **0.806** | **.7894121** | **1.355465** |
| hypertension | **1.124417** | **.0543329** | **2.43** | **0.015** | **1.022814** | **1.236113** |
| dialysis | **1.80623** | **.2320647** | **4.60** | **0.000** | **1.40414** | **2.323462** |
| documented_history_of_cirrhosis | **2.172548** | **.4625406** | **3.64** | **0.000** | **1.431352** | **3.297556** |
| metastasis | **1.457724** | **.2665463** | **2.06** | **0.039** | **1.018667** | **2.08602** |
| active_chemotherapy | **1.309908** | **.3104268** | **1.14** | **0.255** | **.8232273** | **2.084306** |

| obesity | **1.215775** | **.1086634** | **2.19** | **0.029** | **1.02041** | **1.448545** |
| --- | --- | --- | --- | --- | --- | --- |
| ascites | **2.526705** | **1.48737** | **1.57** | **0.115** | **.7970513** | **8.009819** |
| drug_use | **1.109141** | **.0972526** | **1.18** | **0.237** | **.9340087** | **1.317111** |
| smoker | **1.120893** | **.079323** | **1.61** | **0.107** | **.975723** | **1.287661** |
| psych | **1.278487** | **.051932** | **6.05** | **0.000** | **1.180648** | **1.384433** |
| ac_coag | **1.239459** | **.0930025** | **2.86** | **0.004** | **1.069947** | **1.435826** |
| routine_steroid_use | **1.562256** | **.1745257** | **3.99** | **0.000** | **1.255051** | **1.944658** |
| year_admit  2013 | **.9471156** | **.0870378** | **-0.59** | **0.554** | **.7910054** | **1.134035** |
| 2014 | **.9218168** | **.0966249** | **-0.78** | **0.437** | **.7506225** | **1.132055** |
| 2015 | **.8757261** | **.090859** | **-1.28** | **0.201** | **.7145848** | **1.073205** |
| 2016 | **.8573383** | **.0958821** | **-1.38** | **0.169** | **.6885832** | **1.067451** |
| 2017 | **.8133403** | **.1005647** | **-1.67** | **0.095** | **.6383021** | **1.036378** |
| 2018 | **.6775479** | **.0786259** | **-3.35** | **0.001** | **.5397126** | **.8505846** |
| _cons | **.0061948** | **.0012534** | **-25.13** | **0.000** | **.0041668** | **.00921** |

Note: **_cons** estimates baseline odds.

(25,852 observations deleted)

# 80,289 100.00

| isscat | Freq. | Percent | Cum. |
| --- | --- | --- | --- |
| 5-15 | **80,289** | **100.00** | **100.00** |
| Total |  | | |

**comp_infection**

Iteration 0: log pseudolikelihood = **-8239.2149**

Iteration 1: log pseudolikelihood = **-7722.6845**

Iteration 2: log pseudolikelihood = **-7584.1918**

Iteration 3: log pseudolikelihood = **-7580.67**

Iteration 4: log pseudolikelihood = **-7580.6611**

Iteration 5: log pseudolikelihood = **-7580.6611**

Logistic regression Number of obs = **80,289**

Wald chi2(33) = **.**

Prob > chi2 = **.**

Log pseudolikelihood = **-7580.6611** Pseudo R2 = **0.0799**

(Std. Err. adjusted for **35** clusters in traumactr)

comp_infection

diabetes

Robust

Odds Ratio Std. Err. z P>|z| [95% Conf. Interval]

# 1.323113 .0775368 4.78 0.000 1.179546 1.484154

agecat

| 26-45 | **1.250362** | **.1568306** | **1.78** | **0.075** | **.9778477** | **1.598823** |
| --- | --- | --- | --- | --- | --- | --- |
| 46-65 | **2.075754** | **.2720968** | **5.57** | **0.000** | **1.605453** | **2.683826** |
| 66-75 | **2.658861** | **.3700876** | **7.03** | **0.000** | **2.024029** | **3.492808** |
| >75 | **2.639523** | **.3871383** | **6.62** | **0.000** | **1.980067** | **3.518609** |
| male | **1.148956** | **.0914403** | **1.74** | **0.081** | **.983015** | **1.34291** |
| aishn | **2.053596** | **.1539598** | **9.60** | **0.000** | **1.772963** | **2.378649** |
| aisfac | **1.902021** | **.8892842** | **1.38** | **0.169** | **.7607439** | **4.755454** |
| aischs | **2.522605** | **.2217725** | **10.52** | **0.000** | **2.123326** | **2.996966** |
| aisabd | **3.506291** | **.6012651** | **7.32** | **0.000** | **2.505431** | **4.906972** |
| aisext | **1.845687** | **.1293008** | **8.75** | **0.000** | **1.60889** | **2.117334** |
| gcsmcat2  1 | **3.903085** | **.4541706** | **11.70** | **0.000** | **3.107138** | **4.902928** |
| 2 | **5.784341** | **.8539539** | **11.89** | **0.000** | **4.331011** | **7.725356** |
| 99 | **1.036349** | **.1495054** | **0.25** | **0.805** | **.7811066** | **1.374997** |

| pulse2 |  | | | | | |
| --- | --- | --- | --- | --- | --- | --- |
| 1 | **1.849364** | **.2232409** | **5.09** | **0.000** | **1.459728** | **2.343003** |
| 2 | **1.19969** | **.295958** | **0.74** | **0.461** | **.7397437** | **1.945613** |
| 99 | **.9665846** | **.1906104** | **-0.17** | **0.863** | **.6567241** | **1.422646** |
| bp2 1 | **1.746637** | **.2440239** | **3.99** | **0.000** | **1.328253** | **2.296808** |
| 2 | **1.077901** | **.3042959** | **0.27** | **0.790** | **.6198424** | **1.874462** |
| 99 | **.9793704** | **.209527** | **-0.10** | **0.922** | **.6439327** | **1.489544** |
| race | **.9254561** | **.0903484** | **-0.79** | **0.427** | **.7642872** | **1.120611** |
| blunt_ind | **.6093831** | **.0755668** | **-3.99** | **0.000** | **.4778999** | **.777041** |
| transfer | **1.273996** | **.1481663** | **2.08** | **0.037** | **1.014315** | **1.600159** |
| congestive_heart_failure | **1.728924** | **.2107658** | **4.49** | **0.000** | **1.361474** | **2.195544** |
| pvd | **1.034348** | **.1755646** | **0.20** | **0.842** | **.7416323** | **1.442597** |
| hypertension | **1.102387** | **.0585209** | **1.84** | **0.066** | **.9934536** | **1.223265** |
| dialysis | **1.224589** | **.2539207** | **0.98** | **0.329** | **.8156279** | **1.838605** |
| documented_history_of_cirrhosis | **2.274955** | **.3533589** | **5.29** | **0.000** | **1.677874** | **3.08451** |
| metastasis | **1.441299** | **.501664** | **1.05** | **0.294** | **.728587** | **2.851192** |
| active_chemotherapy | **.9913565** | **.3848348** | **-0.02** | **0.982** | **.4632354** | **2.121573** |
| obesity | **1.163591** | **.1160592** | **1.52** | **0.129** | **.9569726** | **1.41482** |
| ascites | **2.364767** | **1.175885** | **1.73** | **0.083** | **.8923331** | **6.266858** |
| drug_use | **1.273689** | **.0962856** | **3.20** | **0.001** | **1.098288** | **1.477103** |
| smoker | **1.264313** | **.1179483** | **2.51** | **0.012** | **1.053042** | **1.517971** |
| psych | **1.328921** | **.0884743** | **4.27** | **0.000** | **1.166352** | **1.51415** |
| ac_coag | **1.370678** | **.095342** | **4.53** | **0.000** | **1.195989** | **1.570882** |
| routine_steroid_use | **1.513844** | **.2240707** | **2.80** | **0.005** | **1.132638** | **2.023351** |
| year_admit  2013 | **.8258377** | **.0678728** | **-2.33** | **0.020** | **.7029709** | **.9701794** |
| 2014 | **.825647** | **.0744236** | **-2.13** | **0.034** | **.6919382** | **.9851934** |
| 2015 | **.6982516** | **.0867743** | **-2.89** | **0.004** | **.5473058** | **.8908281** |
| 2016 | **.4516681** | **.0772004** | **-4.65** | **0.000** | **.3230946** | **.6314066** |
| 2017 | **.4099137** | **.0672235** | **-5.44** | **0.000** | **.2972352** | **.5653072** |
| 2018 | **.3408915** | **.0515513** | **-7.12** | **0.000** | **.2534506** | **.4584997** |
| _cons | **.0093674** | **.0022006** | **-19.88** | **0.000** | **.0059109** | **.014845** |

Note: **_cons** estimates baseline odds.

(25,852 observations deleted)

| isscat | Freq. | Percent | Cum. |
| --- | --- | --- | --- |
| 5-15 | **80,289** | **100.00** | **100.00** |
| Total | **80,289** | **100.00** |  |

| **comp_cardiac**  Iteration 0: log pseudolikelihood | = | **-3189.1176** |  | | |
| --- | --- | --- | --- | --- | --- |
| Iteration 1: log pseudolikelihood | = | **-3071.9783** |  |  |  |
| Iteration 2: log pseudolikelihood | = | **-2804.0836** |  |  |  |
| Iteration 3: log pseudolikelihood | = | **-2791.8234** |  |  |  |
| Iteration 4: log pseudolikelihood | = | **-2791.6273** |  |  |  |
| Iteration 5: log pseudolikelihood | = | **-2791.6271** |  |  |  |
| Logistic regression  Log pseudolikelihood = **-2791.6271** |  |  | Number of obs Wald chi2(33) Prob > chi2 Pseudo R2 | =  =  =  = | **80,289**  **.**  **. 0.1246** |

(Std. Err. adjusted for **35** clusters in traumactr)

| comp_cardiac | Odds Ratio | | Robust Std. Err. | z | P>\|z\| | [95% Conf. | Interval] |
| --- | --- | --- | --- | --- | --- | --- | --- |
| diabetes | **1.428111** | | **.1628356** | **3.13** | **0.002** | **1.142106** | **1.785736** |
| agecat 26-45 | **1.104284** | | **.3278119** | **0.33** | **0.738** | **.61716** | **1.975896** |
| 46-65 | **3.596434** | | **1.040958** | **4.42** | **0.000** | **2.039383** | **6.342279** |
| 66-75 | **5.868306** | | **2.159483** | **4.81** | **0.000** | **2.852846** | **12.07111** |
| >75 | **8.180664** | | **2.402554** | **7.16** | **0.000** | **4.600465** | **14.54706** |
| male | **1.546969** | | **.1364635** | **4.95** | **0.000** | **1.30135** | **1.838947** |
| aishn | **1.439203** | | **.2114109** | **2.48** | **0.013** | **1.079159** | **1.919371** |
| aisfac | **6.3559** | | **3.282995** | **3.58** | **0.000** | **2.309449** | **17.49225** |
| aischs | **1.450388** | | **.2002545** | **2.69** | **0.007** | **1.10652** | **1.901119** |
| aisabd | **2.464011** | | **.4819789** | **4.61** | **0.000** | **1.67935** | **3.615297** |
| aisext | **2.816071** | | **.3311368** | **8.80** | **0.000** | **2.236415** | **3.545969** |
| gcsmcat2  1 | **2.988477** | | **.6926894** | **4.72** | **0.000** | **1.897373** | **4.707032** |
| 2 | **12.1423** | | **2.581976** | **11.74** | **0.000** | **8.003851** | **18.42057** |
| 99 | **1.05121** | | **.1830432** | **0.29** | **0.774** | **.7472622** | **1.478788** |
| pulse2 |  | | | | | | |
| 1 | | **1.971041** | **.3923246** | **3.41** | **0.001** | **1.334347** | **2.911538** |
| 2 | | **1.777334** | **.450689** | **2.27** | **0.023** | **1.081247** | **2.921549** |
| 99 | | **.8446086** | **.2629704** | **-0.54** | **0.588** | **.4588094** | **1.554815** |
| bp2 1 | | **1.902203** | **.4137016** | **2.96** | **0.003** | **1.242032** | **2.913271** |
| 2 | | **5.681637** | **1.888879** | **5.23** | **0.000** | **2.961343** | **10.9008** |
| 99 | | **2.286313** | **.6926854** | **2.73** | **0.006** | **1.262544** | **4.140232** |
| race | | **1.136361** | **.0959022** | **1.51** | **0.130** | **.9631185** | **1.340765** |
| blunt_ind | | **.5463082** | **.0894148** | **-3.69** | **0.000** | **.3963883** | **.7529302** |
| transfer | | **1.001621** | **.1331836** | **0.01** | **0.990** | **.7718283** | **1.299828** |
| congestive_heart_failure | | **1.606415** | **.2319467** | **3.28** | **0.001** | **1.210471** | **2.13187** |
| pvd | | **1.570041** | **.3070127** | **2.31** | **0.021** | **1.070196** | **2.303343** |
| hypertension | | **1.4989** | **.165426** | **3.67** | **0.000** | **1.207341** | **1.860868** |
| dialysis | | **2.848438** | **.5303263** | **5.62** | **0.000** | **1.977556** | **4.102841** |
| documented_history_of_cirrhosis | | **1.901321** | **.7005981** | **1.74** | **0.081** | **.9234319** | **3.914767** |
| metastasis | | **.7906135** | **.4169001** | **-0.45** | **0.656** | **.2812647** | **2.222354** |
| active_chemotherapy | | **.8946971** | **.5198275** | **-0.19** | **0.848** | **.2864969** | **2.794037** |
| obesity | | **1.257748** | **.2234212** | **1.29** | **0.197** | **.8879525** | **1.781548** |
| ascites | | **1.958581** | **1.634984** | **0.81** | **0.421** | **.3813952** | **10.05791** |
| drug_use | | **.9116377** | **.1813201** | **-0.47** | **0.642** | **.6173375** | **1.346238** |
| smoker | | **.8573069** | **.0939328** | **-1.41** | **0.160** | **.6916279** | **1.062674** |
| psych | | **1.032021** | **.1385869** | **0.23** | **0.814** | **.7932009** | **1.342745** |
| ac_coag | | **1.23673** | **.1336293** | **1.97** | **0.049** | **1.000696** | **1.528438** |
| routine_steroid_use | | **2.024423** | **.3399246** | **4.20** | **0.000** | **1.456714** | **2.813378** |
| year_admit  2013 | | **.9435072** | **.1961104** | **-0.28** | **0.780** | **.6277992** | **1.417979** |
| 2014 | | **1.086191** | **.2555268** | **0.35** | **0.725** | **.6849534** | **1.72247** |
| 2015 | | **1.16769** | **.2078834** | **0.87** | **0.384** | **.8237368** | **1.655262** |
| 2016 | | **1.047344** | **.1720798** | **0.28** | **0.778** | **.7589898** | **1.445249** |
| 2017 | | **.9044912** | **.2332658** | **-0.39** | **0.697** | **.5456093** | **1.499433** |
| 2018 | | **1.007133** | **.2454526** | **0.03** | **0.977** | **.6246507** | **1.623816** |
| _cons | | **.0006283** | **.0002607** | **-17.77** | **0.000** | **.0002786** | **.0014171** |
| Note: **_cons** estimates baseline odds. | |  |  |  |  |  |  |

88 .

89 . **For ISS 16-24

90 . outcomes_iss 1

(90,044 observations deleted)

| isscat | Freq. | Percent | Cum. |
| --- | --- | --- | --- |
| 16-24 | **16,097** | **100.00** | **100.00** |
| Total | **16,097** | **100.00** |  |

# dead

note: ascites != 0 predicts failure perfectly ascites dropped and 8 obs not used

Iteration 0: log pseudolikelihood = **-3303.5662**

Iteration 1: log pseudolikelihood = **-2556.0179**

Iteration 2: log pseudolikelihood = **-2314.712**

Iteration 3: log pseudolikelihood = **-2304.9372**

Iteration 4: log pseudolikelihood = **-2304.9127**

Iteration 5: log pseudolikelihood = **-2304.9127**

| Logistic regression  Log pseudolikelihood | = | Number of obs Wald chi2(33) Prob > chi2  **-2304.9127** Pseudo R2 | | | | =  =  =  = | **16,089**  **.**  **. 0.3023** | | |
| --- | --- | --- | --- | --- | --- | --- | --- | --- | --- |
| (Std. Err. adjusted for **35** clusters in traumactr) | | | | | | | | | |
| dead | | | Odds Ratio | Robust Std. Err. | z | P>\|z\| | | [95% Conf. | Interval] |
| diabetes | | | **1.020613** | **.1278443** | **0.16** | **0.871** | | **.7984315** | **1.304622** |
| agecat 26-45 | | | **1.117894** | **.1401792** | **0.89** | **0.374** | | **.8743059** | **1.429347** |
| 46-65 | | | **2.247948** | **.3477798** | **5.24** | **0.000** | | **1.659958** | **3.044217** |
| 66-75 | | | **5.138815** | **1.142782** | **7.36** | **0.000** | | **3.323309** | **7.94612** |
| >75 | | | **13.06508** | **2.680621** | **12.53** | **0.000** | | **8.739107** | **19.53245** |
| male | | | **1.414704** | **.1567604** | **3.13** | **0.002** | | **1.138534** | **1.757865** |
| aishn | | | **.9736995** | **.1044003** | **-0.25** | **0.804** | | **.7891489** | **1.201409** |
| aisfac | | | **.2647717** | **.1346232** | **-2.61** | **0.009** | | **.0977413** | **.7172408** |
| aischs | | | **1.226656** | **.1567284** | **1.60** | **0.110** | | **.9549176** | **1.575723** |
| aisabd | | | **1.277755** | **.2052441** | **1.53** | **0.127** | | **.9326531** | **1.750552** |
| aisext | | | **.9796925** | **.1485049** | **-0.14** | **0.892** | | **.7278836** | **1.318614** |
| gcsmcat2  1 | | | **5.041204** | **.5331029** | **15.30** | **0.000** | | **4.097514** | **6.202233** |
| 2 | | | **26.9744** | **4.204335** | **21.14** | **0.000** | | **19.87376** | **36.61199** |
| 99 | | | **1.749428** | **.2550404** | **3.84** | **0.000** | | **1.31463** | **2.328029** |
| pulse2 | | |  | | | | | | |
| 1 | | | **1.934218** | **.2564507** | **4.98** | **0.000** | | **1.491585** | **2.508205** |
| 2 | | | **2.500041** | **.6998673** | **3.27** | **0.001** | | **1.444308** | **4.327474** |
| 99 | | | **1.296297** | **.3131747** | **1.07** | **0.283** | | **.8073484** | **2.081363** |
| bp2 1 | | | **2.629028** | **.4381747** | **5.80** | **0.000** | | **1.896388** | **3.644712** |
| 2 | | | **3.68451** | **1.046764** | **4.59** | **0.000** | | **2.111323** | **6.429908** |
| 99 | | | **5.206668** | **1.148864** | **7.48** | **0.000** | | **3.378627** | **8.02379** |
| race | | | **1.07714** | **.1085706** | **0.74** | **0.461** | | **.8840461** | **1.312409** |
| blunt_ind | | | **.2458831** | **.0372466** | **-9.26** | **0.000** | | **.1827207** | **.3308793** |
| transfer | | | **.5099623** | **.0679934** | **-5.05** | **0.000** | | **.3926875** | **.6622608** |
| congestive_heart_failure | | | **.9951609** | **.2273689** | **-0.02** | **0.983** | | **.635939** | **1.557296** |

| pvd | **1.644049** | **.6397081** | **1.28** | **0.201** | **.7668449** | **3.524697** |
| --- | --- | --- | --- | --- | --- | --- |
| hypertension | **.7750771** | **.0905282** | **-2.18** | **0.029** | **.6164892** | **.9744607** |
| dialysis | **2.190308** | **.480945** | **3.57** | **0.000** | **1.424294** | **3.368301** |
| documented_history_of_cirrhosis | **5.583936** | **1.636104** | **5.87** | **0.000** | **3.144393** | **9.916173** |
| metastasis | **1.838622** | **.8497371** | **1.32** | **0.188** | **.743193** | **4.548661** |
| active_chemotherapy | **.8735681** | **1.01371** | **-0.12** | **0.907** | **.0898549** | **8.492814** |
| obesity | **1.380783** | **.1976696** | **2.25** | **0.024** | **1.042964** | **1.828022** |
| ascites | **1** | (omitted) |  |  |  |  |
| drug_use | **.5747997** | **.1088818** | **-2.92** | **0.003** | **.396531** | **.8332126** |
| smoker | **.7906573** | **.1000793** | **-1.86** | **0.063** | **.6169436** | **1.013284** |
| psych | **.7464574** | **.0847083** | **-2.58** | **0.010** | **.5975997** | **.9323945** |
| ac_coag | **1.566474** | **.2363106** | **2.98** | **0.003** | **1.165508** | **2.105384** |
| routine_steroid_use | **.7136625** | **.2719809** | **-0.89** | **0.376** | **.3381388** | **1.506228** |
| year_admit  2013 | **.8489545** | **.1186773** | **-1.17** | **0.241** | **.6454951** | **1.116544** |
| 2014 | **.7228122** | **.0964518** | **-2.43** | **0.015** | **.5564696** | **.9388787** |
| 2015 | **.9413296** | **.1262925** | **-0.45** | **0.652** | **.723671** | **1.224453** |
| 2016 | **.9423862** | **.1483838** | **-0.38** | **0.706** | **.6921539** | **1.283084** |
| 2017 | **1.010861** | **.1519202** | **0.07** | **0.943** | **.7529503** | **1.357116** |
| 2018 | **.8281158** | **.1274802** | **-1.23** | **0.221** | **.6124309** | **1.11976** |
| _cons | **.0186788** | **.0054788** | **-13.57** | **0.000** | **.0105118** | **.033191** |
| Note: **_cons** estimates baseline odds. |  |  |  |  |  |  |

(90,044 observations deleted)

| isscat | Freq. | Percent | Cum. |
| --- | --- | --- | --- |
| 16-24 | **16,097** | **100.00** | **100.00** |
| Total | **16,097** | **100.00** |  |

# comp_any

Iteration 0: log pseudolikelihood = **-6149.7028**

Iteration 1: log pseudolikelihood = **-5710.8795**

Iteration 2: log pseudolikelihood = **-5619.7597**

Iteration 3: log pseudolikelihood = **-5619.5547**

Iteration 4: log pseudolikelihood = **-5619.5546**

Logistic regression Number of obs = **16,097**

Wald chi2(33) = **.**

Prob > chi2 = **.**

Log pseudolikelihood = **-5619.5546** Pseudo R2 = **0.0862**

(Std. Err. adjusted for **35** clusters in traumactr)

Robust comp_any Odds Ratio Std. Err.

z

P>|z|

[95% Conf. Interval]

diabetes

**1.133992 .0760452**

**1.88 0.061**

**.9943254**

**1.293276**

| agecat  26-45 | **1.176999** | **.1034619** | **1.85** | **0.064** | **.9907245** | **1.398297** |
| --- | --- | --- | --- | --- | --- | --- |
| 46-65 | **1.608496** | **.1230523** | **6.21** | **0.000** | **1.384529** | **1.868694** |
| 66-75 | **2.157338** | **.2430285** | **6.83** | **0.000** | **1.72993** | **2.690343** |
| >75 | **2.01421** | **.1652559** | **8.53** | **0.000** | **1.715015** | **2.365601** |
| male | **1.28668** | **.0958099** | **3.39** | **0.001** | **1.111956** | **1.488858** |
| aishn | **.8581201** | **.0979518** | **-1.34** | **0.180** | **.6860977** | **1.073273** |
| aisfac | **1.537786** | **.3401003** | **1.95** | **0.052** | **.9968789** | **2.372191** |
| aischs | **1.295927** | **.0875774** | **3.84** | **0.000** | **1.13516** | **1.479462** |
| aisabd | **1.477218** | **.136851** | **4.21** | **0.000** | **1.231937** | **1.771335** |
| aisext | **1.514088** | **.1174652** | **5.35** | **0.000** | **1.30051** | **1.762741** |

| gcsmcat2 |  | | | | | |
| --- | --- | --- | --- | --- | --- | --- |
| 1 | **3.667865** | **.1942135** | **24.54** | **0.000** | **3.306299** | **4.06897** |
| 2 | **4.863041** | **.7027383** | **10.95** | **0.000** | **3.663568** | **6.455228** |
| 99 | **1.60291** | **.2881573** | **2.62** | **0.009** | **1.126905** | **2.279979** |
| pulse2 |  |  |  |  |  |  |
| 1 | **1.757634** | **.1674737** | **5.92** | **0.000** | **1.458219** | **2.118527** |
| 2 | **1.060819** | **.1522419** | **0.41** | **0.681** | **.8007233** | **1.405401** |
| 99 | **.4201415** | **.1764215** | **-2.07** | **0.039** | **.1844878** | **.9568049** |
| bp2 1 | **1.762363** | **.1434229** | **6.96** | **0.000** | **1.502532** | **2.067126** |
| 2 | **1.130358** | **.3089149** | **0.45** | **0.654** | **.6615943** | **1.931259** |
| 99 | **1.576762** | **.4989207** | **1.44** | **0.150** | **.8480629** | **2.931597** |
| race | **1.107557** | **.0684804** | **1.65** | **0.098** | **.9811514** | **1.250247** |
| blunt_ind | **.6071384** | **.0518827** | **-5.84** | **0.000** | **.5135097** | **.7178385** |
| transfer | **.9305042** | **.0862151** | **-0.78** | **0.437** | **.7759808** | **1.115798** |
| congestive_heart_failure | **1.318316** | **.1982618** | **1.84** | **0.066** | **.981764** | **1.770239** |
| pvd | **1.068551** | **.2588755** | **0.27** | **0.784** | **.6646246** | **1.717964** |
| hypertension | **1.021373** | **.0851079** | **0.25** | **0.800** | **.8674742** | **1.202576** |
| dialysis | **1.356546** | **.3121208** | **1.33** | **0.185** | **.8641431** | **2.129527** |
| documented_history_of_cirrhosis | **2.341939** | **.4595719** | **4.34** | **0.000** | **1.594188** | **3.440421** |
| metastasis | **1.308329** | **.5704894** | **0.62** | **0.538** | **.5566159** | **3.075237** |
| active_chemotherapy | **.8920983** | **.4215387** | **-0.24** | **0.809** | **.3533449** | **2.252302** |
| obesity | **1.694238** | **.1661844** | **5.38** | **0.000** | **1.397918** | **2.053369** |
| ascites | **.3640821** | **.3768866** | **-0.98** | **0.329** | **.0478698** | **2.769089** |
| drug_use | **1.066793** | **.0682747** | **1.01** | **0.312** | **.9410299** | **1.209364** |
| smoker | **1.104406** | **.0809695** | **1.35** | **0.176** | **.956584** | **1.275072** |
| psych | **1.044314** | **.083515** | **0.54** | **0.588** | **.8928111** | **1.221526** |
| ac_coag | **1.258423** | **.1234763** | **2.34** | **0.019** | **1.038262** | **1.525269** |
| routine_steroid_use | **.8408428** | **.195626** | **-0.75** | **0.456** | **.5329406** | **1.326633** |
| year_admit  2013 | **.9417604** | **.1018558** | **-0.55** | **0.579** | **.7618667** | **1.164131** |
| 2014 | **.9491223** | **.1159316** | **-0.43** | **0.669** | **.7470527** | **1.20585** |
| 2015 | **.9350392** | **.1246655** | **-0.50** | **0.614** | **.720016** | **1.214276** |
| 2016 | **.8741937** | **.1172446** | **-1.00** | **0.316** | **.6721199** | **1.137021** |
| 2017 | **.756128** | **.0966243** | **-2.19** | **0.029** | **.5886021** | **.9713346** |
| 2018 | **.7758694** | **.1112308** | **-1.77** | **0.077** | **.5858116** | **1.027589** |
| _cons | **.0683852** | **.0132837** | **-13.81** | **0.000** | **.0467323** | **.1000706** |

Note: **_cons** estimates baseline odds.

(90,044 observations deleted)

| isscat | Freq. | Percent | Cum. |
| --- | --- | --- | --- |
| 16-24 | **16,097** | **100.00** | **100.00** |
| Total | **16,097** | **100.00** |  |

# comp_sev

Iteration 0: log pseudolikelihood = **-5547.1178**

Iteration 1: log pseudolikelihood = **-5161.8018**

Iteration 2: log pseudolikelihood = **-5033.1903**

Iteration 3: log pseudolikelihood = **-5032.749**

Iteration 4: log pseudolikelihood = **-5032.7489**

| Logistic regression  Log pseudolikelihood | = | Number of obs Wald chi2(33) Prob > chi2  **-5032.7489** Pseudo R2 | | | | =  =  =  = | **16,097**  **.**  **. 0.0927** | | |
| --- | --- | --- | --- | --- | --- | --- | --- | --- | --- |
| (Std. Err. adjusted for **35** clusters in traumactr) | | | | | | | | | |
| comp_sev | | | Odds Ratio | Robust Std. Err. | z | P>\|z\| | | [95% Conf. | Interval] |
| diabetes | | | **1.127965** | **.0763346** | **1.78** | **0.075** | | **.9878502** | **1.287954** |
| agecat 26-45 | | | **1.257082** | **.122398** | **2.35** | **0.019** | | **1.038687** | **1.521396** |
| 46-65 | | | **1.815161** | **.1679005** | **6.45** | **0.000** | | **1.514189** | **2.175958** |
| 66-75 | | | **2.423069** | **.2939234** | **7.30** | **0.000** | | **1.910351** | **3.073395** |
| >75 | | | **2.135784** | **.2281636** | **7.10** | **0.000** | | **1.732305** | **2.63324** |
| male | | | **1.468158** | **.1171712** | **4.81** | **0.000** | | **1.255567** | **1.716745** |
| aishn | | | **.8699907** | **.1088589** | **-1.11** | **0.266** | | **.68078** | **1.111789** |
| aisfac | | | **1.472633** | **.3844323** | **1.48** | **0.138** | | **.8828512** | **2.456414** |
| aischs | | | **1.343511** | **.1031234** | **3.85** | **0.000** | | **1.155862** | **1.561625** |
| aisabd | | | **1.389239** | **.1287156** | **3.55** | **0.000** | | **1.158541** | **1.665875** |
| aisext | | | **1.409165** | **.1048098** | **4.61** | **0.000** | | **1.218013** | **1.630316** |
| gcsmcat2  1 | | | **3.611004** | **.2107625** | **22.00** | **0.000** | | **3.22067** | **4.048647** |
| 2 | | | **5.635612** | **.7924834** | **12.30** | **0.000** | | **4.278037** | **7.423993** |
| 99 | | | **1.658621** | **.2622094** | **3.20** | **0.001** | | **1.216695** | **2.261063** |
| pulse2 | | |  | | | | | | |
| 1 | | | **1.827355** | **.1854871** | **5.94** | **0.000** | | **1.497686** | **2.229589** |
| 2 | | | **1.198071** | **.164987** | **1.31** | **0.189** | | **.9146676** | **1.569286** |
| 99 | | | **.3705222** | **.1811632** | **-2.03** | **0.042** | | **.1421109** | **.966053** |
| bp2 1 | | | **1.841367** | **.1400229** | **8.03** | **0.000** | | **1.586399** | **2.137313** |
| 2 | | | **1.136149** | **.3285799** | **0.44** | **0.659** | | **.6445602** | **2.002659** |
| 99 | | | **1.597866** | **.5117367** | **1.46** | **0.143** | | **.8529671** | **2.993287** |
| race | | | **1.116668** | **.0814807** | **1.51** | **0.130** | | **.9678632** | **1.288352** |
| blunt_ind | | | **.7116099** | **.0657537** | **-3.68** | **0.000** | | **.593731** | **.8528923** |
| transfer | | | **.8563275** | **.0868634** | **-1.53** | **0.126** | | **.7019344** | **1.04468** |
| congestive_heart_failure | | | **1.110333** | **.1769347** | **0.66** | **0.511** | | **.8124784** | **1.517381** |
| pvd | | | **1.187574** | **.25431** | **0.80** | **0.422** | | **.7805171** | **1.806921** |
| hypertension | | | **.9979485** | **.0822083** | **-0.02** | **0.980** | | **.8491579** | **1.17281** |
| dialysis | | | **1.684268** | **.3813295** | **2.30** | **0.021** | | **1.080671** | **2.624998** |
| documented_history_of_cirrhosis | | | **2.122249** | **.4838786** | **3.30** | **0.001** | | **1.357438** | **3.317972** |
| metastasis | | | **1.482734** | **.6919468** | **0.84** | **0.399** | | **.594064** | **3.700778** |
| active_chemotherapy | | | **1.043489** | **.5008517** | **0.09** | **0.929** | | **.4073142** | **2.673288** |
| obesity | | | **1.851342** | **.1900167** | **6.00** | **0.000** | | **1.513986** | **2.263871** |
| ascites | | | **.4808154** | **.542709** | **-0.65** | **0.516** | | **.0526265** | **4.392906** |
| drug_use | | | **1.016744** | **.0614268** | **0.27** | **0.783** | | **.9032043** | **1.144556** |
| smoker | | | **1.170066** | **.0890516** | **2.06** | **0.039** | | **1.007923** | **1.358294** |
| psych | | | **1.018293** | **.0882614** | **0.21** | **0.834** | | **.8592002** | **1.206845** |
| ac_coag | | | **1.234847** | **.1346118** | **1.94** | **0.053** | | **.9972928** | **1.528985** |
| routine_steroid_use | | | **.8784682** | **.2142581** | **-0.53** | **0.595** | | **.5446509** | **1.416882** |
| year_admit  2013 | | | **.9321107** | **.0983863** | **-0.67** | **0.505** | | **.7579164** | **1.146341** |
| 2014 | | | **.9526857** | **.126684** | **-0.36** | **0.715** | | **.734109** | **1.236342** |
| 2015 | | | **1.00323** | **.1410493** | **0.02** | **0.982** | | **.7615982** | **1.321525** |
| 2016 | | | **.9932677** | **.1338635** | **-0.05** | **0.960** | | **.7626919** | **1.293551** |
| 2017 | | | **.872536** | **.1070318** | **-1.11** | **0.266** | | **.6860703** | **1.109681** |
| 2018 | | | **.9088945** | **.1217542** | **-0.71** | **0.476** | | **.6990169** | **1.181787** |
| _cons | | | **.0376928** | **.0087711** | **-14.09** | **0.000** | | **.0238882** | **.0594748** |

Note: **_cons** estimates baseline odds.

(90,044 observations deleted)

# 16,097 100.00

| isscat | Freq. | Percent | Cum. |
| --- | --- | --- | --- |
| 16-24 | **16,097** | **100.00** | **100.00** |
| Total |  | | |

**comp_infection**

note: ascites != 0 predicts failure perfectly ascites dropped and 8 obs not used

Iteration 0: log pseudolikelihood = **-4489.7105**

Iteration 1: log pseudolikelihood = **-4238.6047**

Iteration 2: log pseudolikelihood = **-4105.4675**

Iteration 3: log pseudolikelihood = **-4104.9989**

Iteration 4: log pseudolikelihood = **-4104.9988**

| Logistic regression  Log pseudolikelihood | Number of obs Wald chi2(33) Prob > chi2  = **-4104.9988** Pseudo R2 | | | | =  =  =  = | **16,089**  **.**  **. 0.0857** | | |
| --- | --- | --- | --- | --- | --- | --- | --- | --- |
| (Std. Err. adjusted for **35** clusters in traumactr) | | | | | | | | |
| comp_infection | | Odds Ratio | Robust Std. Err. | z | P>\|z\| | | [95% Conf. | Interval] |
| diabetes | | **1.254942** | **.0861999** | **3.31** | **0.001** | | **1.096872** | **1.435791** |
| agecat 26-45 | | **1.130057** | **.1010268** | **1.37** | **0.171** | | **.9484254** | **1.346473** |
| 46-65 | | **1.393136** | **.0922428** | **5.01** | **0.000** | | **1.223583** | **1.586184** |
| 66-75 | | **1.801955** | **.2062503** | **5.14** | **0.000** | | **1.439847** | **2.255131** |
| >75 | | **1.61492** | **.1489751** | **5.20** | **0.000** | | **1.347809** | **1.934968** |
| male | | **1.216133** | **.0977224** | **2.44** | **0.015** | | **1.038922** | **1.423572** |
| aishn | | **1.03748** | **.1418739** | **0.27** | **0.788** | | **.7935585** | **1.356376** |
| aisfac | | **2.51605** | **.5607753** | **4.14** | **0.000** | | **1.625564** | **3.894345** |
| aischs | | **1.327861** | **.1284239** | **2.93** | **0.003** | | **1.098573** | **1.605005** |
| aisabd | | **1.550591** | **.1510955** | **4.50** | **0.000** | | **1.281011** | **1.876902** |
| aisext | | **1.424698** | **.1518327** | **3.32** | **0.001** | | **1.156135** | **1.755646** |
| gcsmcat2  1 | | **4.454667** | **.3092027** | **21.52** | **0.000** | | **3.888056** | **5.103851** |
| 2 | | **5.122569** | **.9536588** | **8.78** | **0.000** | | **3.556487** | **7.378267** |
| 99 | | **1.609903** | **.3543304** | **2.16** | **0.031** | | **1.045815** | **2.478245** |
| pulse2 | |  | | | | | | |
| 1 | | **1.425232** | **.2092608** | **2.41** | **0.016** | | **1.068827** | **1.900483** |
| 2 | | **.4552013** | **.2241576** | **-1.60** | **0.110** | | **.1733968** | **1.194995** |
| 99 | | **.3957015** | **.1705404** | **-2.15** | **0.031** | | **.1700258** | **.9209171** |
| bp2 1 | | **1.435749** | **.1626439** | **3.19** | **0.001** | | **1.149882** | **1.792685** |
| 2 | | **.6280669** | **.2484831** | **-1.18** | **0.240** | | **.2892302** | **1.363855** |
| 99 | | **1.525203** | **.5797751** | **1.11** | **0.267** | | **.7240366** | **3.212882** |
| race | | **1.144566** | **.0890859** | **1.73** | **0.083** | | **.9826269** | **1.333193** |
| blunt_ind | | **.7138411** | **.0922769** | **-2.61** | **0.009** | | **.5540748** | **.9196757** |
| transfer | | **.9902811** | **.0989706** | **-0.10** | **0.922** | | **.8141187** | **1.204562** |
| congestive_heart_failure | | **1.503546** | **.3208637** | **1.91** | **0.056** | | **.9896154** | **2.284373** |

| pvd | **1.154578** | **.4178266** | **0.40** | **0.691** | **.5680476** | **2.346724** |
| --- | --- | --- | --- | --- | --- | --- |
| hypertension | **1.080247** | **.0708097** | **1.18** | **0.239** | **.9500074** | **1.228341** |
| dialysis | **.6692521** | **.2202855** | **-1.22** | **0.222** | **.3510873** | **1.275746** |
| documented_history_of_cirrhosis | **1.947638** | **.5126647** | **2.53** | **0.011** | **1.162657** | **3.262607** |
| metastasis | **1.709275** | **.7160691** | **1.28** | **0.201** | **.751998** | **3.885143** |
| active_chemotherapy | **.547846** | **.3225804** | **-1.02** | **0.307** | **.1727657** | **1.737239** |
| obesity | **1.620157** | **.1810214** | **4.32** | **0.000** | **1.301523** | **2.016799** |
| ascites | **1** | (omitted) |  |  |  |  |
| drug_use | **1.153759** | **.112855** | **1.46** | **0.144** | **.9524776** | **1.397576** |
| smoker | **1.153271** | **.0850942** | **1.93** | **0.053** | **.9979881** | **1.332715** |
| psych | **1.211669** | **.1064343** | **2.19** | **0.029** | **1.020031** | **1.43931** |
| ac_coag | **1.085671** | **.1178306** | **0.76** | **0.449** | **.8776373** | **1.343016** |
| routine_steroid_use | **.8438582** | **.2840634** | **-0.50** | **0.614** | **.4362487** | **1.632318** |
| year_admit  2013 | **.9800318** | **.1306704** | **-0.15** | **0.880** | **.7546527** | **1.272721** |
| 2014 | **.905692** | **.1176704** | **-0.76** | **0.446** | **.702085** | **1.168346** |
| 2015 | **.8318806** | **.121378** | **-1.26** | **0.207** | **.6249768** | **1.107282** |
| 2016 | **.6296237** | **.0857683** | **-3.40** | **0.001** | **.4820911** | **.8223053** |
| 2017 | **.5404242** | **.0745521** | **-4.46** | **0.000** | **.4123923** | **.708205** |
| 2018 | **.5939297** | **.1033259** | **-2.99** | **0.003** | **.4223296** | **.8352538** |
| _cons | **.0392674** | **.0085049** | **-14.95** | **0.000** | **.0256845** | **.0600333** |
| Note: **_cons** estimates baseline odds. |  |  |  |  |  |  |

(90,044 observations deleted)

| isscat | Freq. | Percent | Cum. |
| --- | --- | --- | --- |
| 16-24 | **16,097** | **100.00** | **100.00** |
| Total | **16,097** | **100.00** |  |

# comp_cardiac

note: metastasis != 0 predicts failure perfectly metastasis dropped and 68 obs not used

note: active_chemotherapy != 0 predicts failure perfectly active_chemotherapy dropped and 37 obs not used

note: ascites != 0 predicts failure perfectly ascites dropped and 7 obs not used

Iteration 0: log pseudolikelihood = **-1509.5953**

Iteration 1: log pseudolikelihood = **-1359.4229**

Iteration 2: log pseudolikelihood = **-1295.5322**

Iteration 3: log pseudolikelihood = **-1294.6764**

Iteration 4: log pseudolikelihood = **-1294.6744**

Iteration 5: log pseudolikelihood = **-1294.6744**

| Logistic regression |  | Number of obs | = | **15,985** |
| --- | --- | --- | --- | --- |
|  |  | Wald chi2(33) | = | **.** |
|  |  | Prob > chi2 | = | **.** |
| Log pseudolikelihood | = **-1294.6744** | Pseudo R2 | = | **0.1424** |

(Std. Err. adjusted for **35** clusters in traumactr)

| comp_cardiac | Odds Ratio | | Robust Std. Err. | z | P>\|z\| | [95% Conf. | Interval] |
| --- | --- | --- | --- | --- | --- | --- | --- |
| diabetes | **1.331059** | | **.2371865** | **1.60** | **0.109** | **.938682** | **1.887453** |
| agecat 26-45 | **1.099629** | | **.2295328** | **0.45** | **0.649** | **.7304145** | **1.655477** |
| 46-65 | **2.538188** | | **.6375008** | **3.71** | **0.000** | **1.551432** | **4.152549** |
| 66-75 | **3.806533** | | **.9618949** | **5.29** | **0.000** | **2.319715** | **6.246326** |
| >75 | **4.257117** | | **1.011689** | **6.10** | **0.000** | **2.671963** | **6.782669** |
| male | **1.141159** | | **.172747** | **0.87** | **0.383** | **.8481891** | **1.535324** |
| aishn | **.5205232** | | **.1285295** | **-2.64** | **0.008** | **.3208176** | **.8445433** |
| aisfac | **.371776** | | **.3944719** | **-0.93** | **0.351** | **.046464** | **2.974722** |
| aischs | **1.1646** | | **.2293005** | **0.77** | **0.439** | **.7917384** | **1.713056** |
| aisabd | **1.181096** | | **.2661636** | **0.74** | **0.460** | **.7593886** | **1.836987** |
| aisext | **1.25233** | | **.2546474** | **1.11** | **0.268** | **.8406912** | **1.865526** |
| gcsmcat2  1 | **2.547363** | | **.4252568** | **5.60** | **0.000** | **1.836501** | **3.533381** |
| 2 | **6.318612** | | **1.504216** | **7.74** | **0.000** | **3.962631** | **10.07534** |
| 99 | **1.676491** | | **.3309662** | **2.62** | **0.009** | **1.138572** | **2.468551** |
| pulse2 |  | | | | | | |
| 1 | | **1.541024** | **.3303571** | **2.02** | **0.044** | **1.012355** | **2.345772** |
| 2 | | **1.615554** | **.4269143** | **1.82** | **0.069** | **.962475** | **2.711775** |
| 99 | | **.6120498** | **.3621782** | **-0.83** | **0.407** | **.1919072** | **1.95201** |
| bp2 1 | | **2.476504** | **.4452428** | **5.04** | **0.000** | **1.741022** | **3.522685** |
| 2 | | **3.617335** | **1.02857** | **4.52** | **0.000** | **2.07183** | **6.315728** |
| 99 | | **2.01233** | **1.02074** | **1.38** | **0.168** | **.7446174** | **5.438324** |
| race | | **1.570113** | **.2288007** | **3.10** | **0.002** | **1.180027** | **2.089152** |
| blunt_ind | | **.4478466** | **.0949185** | **-3.79** | **0.000** | **.2956121** | **.6784791** |
| transfer | | **.599419** | **.1307026** | **-2.35** | **0.019** | **.3909552** | **.9190392** |
| congestive_heart_failure | | **1.179227** | **.380362** | **0.51** | **0.609** | **.6266722** | **2.218984** |
| pvd | | **1.234002** | **.631451** | **0.41** | **0.681** | **.4526343** | **3.364217** |
| hypertension | | **1.084646** | **.1661705** | **0.53** | **0.596** | **.8033077** | **1.464516** |
| dialysis | | **2.356095** | **.8415899** | **2.40** | **0.016** | **1.169893** | **4.745034** |
| documented_history_of_cirrhosis | | **5.104663** | **2.053793** | **4.05** | **0.000** | **2.320051** | **11.23147** |
| metastasis | | **1** | (omitted) |  |  |  |  |
| active_chemotherapy | | **1** | (omitted) |  |  |  |  |
| obesity | | **1.188031** | **.24481** | **0.84** | **0.403** | **.7932792** | **1.779219** |
| ascites | | **1** | (omitted) |  |  |  |  |
| drug_use | | **1.015595** | **.1465684** | **0.11** | **0.915** | **.7653802** | **1.34761** |
| smoker | | **.6726836** | **.1060403** | **-2.52** | **0.012** | **.493889** | **.9162044** |
| psych | | **.6884675** | **.1627563** | **-1.58** | **0.114** | **.4331679** | **1.094235** |
| ac_coag | | **1.783366** | **.3246083** | **3.18** | **0.001** | **1.248259** | **2.547864** |
| routine_steroid_use | | **.713298** | **.4395017** | **-0.55** | **0.583** | **.213206** | **2.386397** |
| year_admit  2013 | | **1.157609** | **.3025287** | **0.56** | **0.575** | **.693601** | **1.932032** |
| 2014 | | **1.030605** | **.2877144** | **0.11** | **0.914** | **.5962957** | **1.78124** |
| 2015 | | **1.830687** | **.5127203** | **2.16** | **0.031** | **1.057348** | **3.169641** |
| 2016 | | **1.802429** | **.4807936** | **2.21** | **0.027** | **1.068568** | **3.040284** |
| 2017 | | **1.400667** | **.3451244** | **1.37** | **0.171** | **.8641695** | **2.270236** |
| 2018 | | **1.726173** | **.4704697** | **2.00** | **0.045** | **1.011786** | **2.944964** |
| _cons | | **.0065572** | **.0030828** | **-10.69** | **0.000** | **.0026093** | **.016478** |
| Note: **_cons** estimates baseline odds. | |  |  |  |  |  |  |

91 .

92 . **For ISS 25-35

93 . outcomes_iss 2

(98,399 observations deleted)

| isscat | Freq. | Percent | Cum. | |  |
| --- | --- | --- | --- | --- | --- |
| 25-35 | **7,742** | **100.00** | **100.00** | |  |
| Total | **7,742** | **100.00** |  | |  |
| **dead**  Iteration 0: log pseudolikelihood | | | = | **-4465.7136** |  |
| Iteration 1: log pseudolikelihood | | | = | **-3119.5751** |  |
| Iteration 2: log pseudolikelihood | | | = | **-3004.0105** |  |
| Iteration 3: log pseudolikelihood | | | = | **-3001.3317** |  |
| Iteration 4: log pseudolikelihood | | | = | **-3001.3282** |  |
| Iteration 5: log pseudolikelihood | | | = | **-3001.3282** |  |
| Logistic regression  Log pseudolikelihood = **-3001.3282** | | |  |  | Number of obs = **7,742**  Wald chi2(33) = **.**  Prob > chi2 = **.**  Pseudo R2 = **0.3279** |
|  | | |  |  | (Std. Err. adjusted for **35** clusters in traumactr) |

| dead | Odds Ratio | Robust Std. Err. | z | P>\|z\| | [95% Conf. | Interval] |
| --- | --- | --- | --- | --- | --- | --- |
| diabetes | **1.077679** | **.1150885** | **0.70** | **0.484** | **.8741524** | **1.328592** |
| agecat 26-45 | **1.209959** | **.1338787** | **1.72** | **0.085** | **.9740637** | **1.502982** |
| 46-65 | **2.039932** | **.2551532** | **5.70** | **0.000** | **1.596423** | **2.606654** |
| 66-75 | **2.786754** | **.4355399** | **6.56** | **0.000** | **2.051469** | **3.785579** |
| >75 | **4.654538** | **.81796** | **8.75** | **0.000** | **3.298311** | **6.568429** |
| male | **1.140461** | **.1020254** | **1.47** | **0.142** | **.9570447** | **1.359029** |
| aishn | **.8700904** | **.1129507** | **-1.07** | **0.284** | **.6746301** | **1.122181** |
| aisfac | **.3885565** | **.0719549** | **-5.10** | **0.000** | **.2702864** | **.5585782** |
| aischs | **.6230206** | **.0556431** | **-5.30** | **0.000** | **.5229739** | **.7422066** |
| aisabd | **.8214381** | **.1030142** | **-1.57** | **0.117** | **.6424333** | **1.05032** |
| aisext | **.7904224** | **.0661155** | **-2.81** | **0.005** | **.6709032** | **.9312336** |
| gcsmcat2  1 | **4.91419** | **.4983843** | **15.70** | **0.000** | **4.028333** | **5.994854** |
| 2 | **24.19587** | **2.499801** | **30.84** | **0.000** | **19.76056** | **29.6267** |
| 99 | **2.072219** | **.4860474** | **3.11** | **0.002** | **1.308528** | **3.281622** |
| pulse2 |  | | | | | |
| 1 | **1.478463** | **.1681951** | **3.44** | **0.001** | **1.182973** | **1.847762** |
| 2 | **1.974696** | **.4271885** | **3.15** | **0.002** | **1.292286** | **3.017462** |
| 99 | **1.758525** | **.4987047** | **1.99** | **0.047** | **1.008682** | **3.065794** |
| bp2 1 | **1.764768** | **.1885802** | **5.32** | **0.000** | **1.431296** | **2.175933** |
| 2 | **1.469551** | **.4203656** | **1.35** | **0.178** | **.8388772** | **2.574372** |
| 99 | **1.830289** | **.4093038** | **2.70** | **0.007** | **1.180775** | **2.837084** |
| race | **.7913483** | **.0935634** | **-1.98** | **0.048** | **.6276647** | **.9977177** |
| blunt_ind | **.2683872** | **.0337472** | **-10.46** | **0.000** | **.2097641** | **.3433937** |
| transfer | **.5430776** | **.0490367** | **-6.76** | **0.000** | **.4549916** | **.648217** |
| congestive_heart_failure | **1.404426** | **.2596149** | **1.84** | **0.066** | **.9775756** | **2.017659** |
| pvd | **.9297212** | **.3535949** | **-0.19** | **0.848** | **.441185** | **1.959227** |
| hypertension | **.8093687** | **.0693175** | **-2.47** | **0.014** | **.6842994** | **.957297** |
| dialysis | **1.558417** | **.5061315** | **1.37** | **0.172** | **.8245868** | **2.945308** |

| documented_history_of_cirrhosis | **2.874128** | **1.084447** | **2.80** | **0.005** | **1.371944** | **6.0211** |
| --- | --- | --- | --- | --- | --- | --- |
| metastasis | **2.344237** | **1.241599** | **1.61** | **0.108** | **.8301797** | **6.619588** |
| active_chemotherapy | **.8300211** | **.3156801** | **-0.49** | **0.624** | **.3938706** | **1.749141** |
| obesity | **.8897599** | **.1353243** | **-0.77** | **0.442** | **.6604088** | **1.198761** |
| ascites | **.3415278** | **.7188723** | **-0.51** | **0.610** | **.0055177** | **21.13959** |
| drug_use | **.6636408** | **.0572887** | **-4.75** | **0.000** | **.560342** | **.7859827** |
| smoker | **.4815286** | **.0543517** | **-6.47** | **0.000** | **.3859617** | **.6007585** |
| psych | **.9528424** | **.0782614** | **-0.59** | **0.556** | **.8111627** | **1.119268** |
| ac_coag | **1.446947** | **.1378637** | **3.88** | **0.000** | **1.20047** | **1.744032** |
| routine_steroid_use | **1.907417** | **.4635254** | **2.66** | **0.008** | **1.184659** | **3.071128** |
| year_admit  2013 | **1.100808** | **.1211234** | **0.87** | **0.383** | **.8872637** | **1.365748** |
| 2014 | **1.202554** | **.1482271** | **1.50** | **0.135** | **.9444637** | **1.531172** |
| 2015 | **1.091085** | **.1255103** | **0.76** | **0.449** | **.8708492** | **1.367019** |
| 2016 | **1.052162** | **.153662** | **0.35** | **0.728** | **.7902594** | **1.400863** |
| 2017 | **.9932177** | **.127587** | **-0.05** | **0.958** | **.772148** | **1.277581** |
| 2018 | **1.059974** | **.1225809** | **0.50** | **0.615** | **.8450019** | **1.329635** |
| _cons | **.1946397** | **.0314052** | **-10.14** | **0.000** | **.1418697** | **.2670382** |

Note: **_cons** estimates baseline odds.

(98,399 observations deleted)

| isscat | Freq. | Percent | Cum. |
| --- | --- | --- | --- |
| 25-35 | **7,742** | **100.00** | **100.00** |
| Total | **7,742** | **100.00** |  |

# comp_any

Iteration 0: log pseudolikelihood = **-4497.2381**

Iteration 1: log pseudolikelihood = **-4242.095**

Iteration 2: log pseudolikelihood = **-4236.4021**

Iteration 3: log pseudolikelihood = **-4236.3849**

Iteration 4: log pseudolikelihood = **-4236.3849**

Logistic regression Number of obs = **7,742**

Wald chi2(33) = **.**

Prob > chi2 = **.**

Log pseudolikelihood = **-4236.3849** Pseudo R2 = **0.0580**

(Std. Err. adjusted for **35** clusters in traumactr)

| comp_any diabetes agecat | Robust  Odds Ratio Std. Err. z P>\|z\| [95% Conf. Interval]  **1.220044 .120357 2.02 0.044 1.005553 1.480289** | | | | | |
| --- | --- | --- | --- | --- | --- | --- |
| 26-45 | **1.318839** | **.1429126** | **2.55** | **0.011** | **1.066482** | **1.63091** |
| 46-65 | **1.605054** | **.1730634** | **4.39** | **0.000** | **1.299301** | **1.982758** |
| 66-75 | **1.434124** | **.1690928** | **3.06** | **0.002** | **1.138215** | **1.806962** |
| >75 | **.9667422** | **.1224067** | **-0.27** | **0.789** | **.7542815** | **1.239047** |
| male | **1.278923** | **.0776263** | **4.05** | **0.000** | **1.13548** | **1.440487** |
| aishn | **1.117389** | **.0803553** | **1.54** | **0.123** | **.9704907** | **1.286522** |
| aisfac | **.8963265** | **.1490568** | **-0.66** | **0.510** | **.6470138** | **1.241706** |
| aischs | **1.35963** | **.098481** | **4.24** | **0.000** | **1.179686** | **1.567022** |
| aisabd | **1.282753** | **.0825403** | **3.87** | **0.000** | **1.130763** | **1.455173** |
| aisext | **1.544671** | **.118047** | **5.69** | **0.000** | **1.329797** | **1.794265** |
| gcsmcat2  1 | **2.198737** | **.1486189** | **11.66** | **0.000** | **1.92592** | **2.510201** |

| 2 | **1.691957** | **.1472799** | **6.04** | **0.000** | **1.426576** | **2.006707** |
| --- | --- | --- | --- | --- | --- | --- |
| 99 | **1.68232** | **.1919827** | **4.56** | **0.000** | **1.345152** | **2.104001** |
| pulse2 |  |  |  |  |  |  |
| 1 | **1.49234** | **.1153749** | **5.18** | **0.000** | **1.282509** | **1.736503** |
| 2 | **.9918122** | **.1896709** | **-0.04** | **0.966** | **.6817866** | **1.442814** |
| 99 | **.4745516** | **.1334696** | **-2.65** | **0.008** | **.273451** | **.823545** |
| bp2 1 | **1.622411** | **.169673** | **4.63** | **0.000** | **1.321727** | **1.9915** |
| 2 | **1.15873** | **.2925848** | **0.58** | **0.560** | **.7063986** | **1.900705** |
| 99 | **1.168031** | **.2634643** | **0.69** | **0.491** | **.7506805** | **1.817414** |
| race | **1.222018** | **.0973824** | **2.52** | **0.012** | **1.045311** | **1.428597** |
| blunt_ind | **.8768344** | **.0917051** | **-1.26** | **0.209** | **.7143209** | **1.076321** |
| transfer | **1.178038** | **.1266095** | **1.52** | **0.127** | **.9542814** | **1.45426** |
| congestive_heart_failure | **1.132633** | **.1660149** | **0.85** | **0.395** | **.8498163** | **1.509571** |
| pvd | **1.88419** | **.7148202** | **1.67** | **0.095** | **.8957725** | **3.963253** |
| hypertension | **.9698958** | **.0693064** | **-0.43** | **0.669** | **.8431412** | **1.115706** |
| dialysis | **.6238932** | **.2159649** | **-1.36** | **0.173** | **.3165637** | **1.229587** |
| documented_history_of_cirrhosis | **1.293684** | **.3505596** | **0.95** | **0.342** | **.7606268** | **2.200315** |
| metastasis | **.3337022** | **.1465905** | **-2.50** | **0.012** | **.1410713** | **.789368** |
| active_chemotherapy | **.5582747** | **.4322907** | **-0.75** | **0.452** | **.1223867** | **2.546605** |
| obesity | **1.365839** | **.1334527** | **3.19** | **0.001** | **1.127796** | **1.654125** |
| ascites | **1.198023** | **1.079246** | **0.20** | **0.841** | **.2049536** | **7.002853** |
| drug_use | **1.095227** | **.0715788** | **1.39** | **0.164** | **.9635488** | **1.2449** |
| smoker | **1.000363** | **.0635865** | **0.01** | **0.995** | **.8831866** | **1.133086** |
| psych | **1.010847** | **.0876259** | **0.12** | **0.901** | **.8529003** | **1.198043** |
| ac_coag | **1.057169** | **.1243019** | **0.47** | **0.636** | **.8395767** | **1.331156** |
| routine_steroid_use | **1.553659** | **.3291792** | **2.08** | **0.038** | **1.025673** | **2.353438** |
| year_admit  2013 | **.9625224** | **.0990487** | **-0.37** | **0.710** | **.7867154** | **1.177617** |
| 2014 | **.9090915** | **.1247366** | **-0.69** | **0.487** | **.6947269** | **1.1896** |
| 2015 | **1.001881** | **.1567292** | **0.01** | **0.990** | **.7373241** | **1.361363** |
| 2016 | **.8610079** | **.113706** | **-1.13** | **0.257** | **.6646548** | **1.115368** |
| 2017 | **.7538976** | **.1099783** | **-1.94** | **0.053** | **.5664211** | **1.003426** |
| 2018 | **.7658288** | **.1276405** | **-1.60** | **0.109** | **.5524107** | **1.061699** |
| _cons | **.1239278** | **.0259209** | **-9.98** | **0.000** | **.082249** | **.1867269** |

Note: **_cons** estimates baseline odds.

(98,399 observations deleted)

| isscat | Freq. | Percent | Cum. |
| --- | --- | --- | --- |
| 25-35 | **7,742** | **100.00** | **100.00** |
| Total | **7,742** | **100.00** |  |

| **comp_sev**  Iteration 0: log pseudolikelihood | = | **-4288.143** |  | | |
| --- | --- | --- | --- | --- | --- |
| Iteration 1: log pseudolikelihood | = | **-4035.765** |  |  |  |
| Iteration 2: log pseudolikelihood | = | **-4028.2775** |  |  |  |
| Iteration 3: log pseudolikelihood | = | **-4028.2353** |  |  |  |
| Iteration 4: log pseudolikelihood | = | **-4028.2352** |  |  |  |
| Logistic regression  Log pseudolikelihood = **-4028.2352** |  |  | Number of obs Wald chi2(33) Prob > chi2 Pseudo R2 | =  =  =  = | **7,742**  **.**  **. 0.0606** |

(Std. Err. adjusted for **35** clusters in traumactr)

| comp_sev | Odds Ratio | | Robust Std. Err. | z | P>\|z\| | [95% Conf. | Interval] |
| --- | --- | --- | --- | --- | --- | --- | --- |
| diabetes | **1.30362** | | **.1333318** | **2.59** | **0.010** | **1.066821** | **1.59298** |
| agecat 26-45 | **1.310589** | | **.1448372** | **2.45** | **0.014** | **1.055353** | **1.627554** |
| 46-65 | **1.688443** | | **.1982724** | **4.46** | **0.000** | **1.341315** | **2.125408** |
| 66-75 | **1.436394** | | **.1838005** | **2.83** | **0.005** | **1.117774** | **1.845836** |
| >75 | **.9574357** | | **.1397063** | **-0.30** | **0.766** | **.7192911** | **1.274426** |
| male | **1.447994** | | **.0960598** | **5.58** | **0.000** | **1.271447** | **1.649056** |
| aishn | **1.108363** | | **.0843189** | **1.35** | **0.176** | **.9548313** | **1.286581** |
| aisfac | **.9470979** | | **.1627509** | **-0.32** | **0.752** | **.6762744** | **1.326376** |
| aischs | **1.373127** | | **.10341** | **4.21** | **0.000** | **1.184695** | **1.591529** |
| aisabd | **1.189079** | | **.0852208** | **2.42** | **0.016** | **1.03325** | **1.368409** |
| aisext | **1.489802** | | **.1198559** | **4.96** | **0.000** | **1.272473** | **1.744249** |
| gcsmcat2  1 | **2.242535** | | **.1338467** | **13.53** | **0.000** | **1.994963** | **2.52083** |
| 2 | **1.797547** | | **.1583635** | **6.66** | **0.000** | **1.51248** | **2.136343** |
| 99 | **1.781314** | | **.2025295** | **5.08** | **0.000** | **1.425482** | **2.22597** |
| pulse2 |  | | | | | | |
| 1 | | **1.518348** | **.1134256** | **5.59** | **0.000** | **1.311547** | **1.757757** |
| 2 | | **1.097358** | **.2089878** | **0.49** | **0.626** | **.7555103** | **1.593884** |
| 99 | | **.4122009** | **.0994106** | **-3.67** | **0.000** | **.2569357** | **.6612923** |
| bp2 1 | | **1.628328** | **.1676211** | **4.74** | **0.000** | **1.330818** | **1.992348** |
| 2 | | **1.253476** | **.3198858** | **0.89** | **0.376** | **.7601343** | **2.067006** |
| 99 | | **1.320788** | **.2765626** | **1.33** | **0.184** | **.8761901** | **1.990985** |
| race | | **1.184582** | **.0875178** | **2.29** | **0.022** | **1.024891** | **1.369155** |
| blunt_ind | | **.8837669** | **.086655** | **-1.26** | **0.208** | **.7292489** | **1.071025** |
| transfer | | **1.113999** | **.1069235** | **1.12** | **0.261** | **.9229644** | **1.344573** |
| congestive_heart_failure | | **1.193045** | **.1681695** | **1.25** | **0.210** | **.9050511** | **1.572682** |
| pvd | | **2.124614** | **.7951175** | **2.01** | **0.044** | **1.020295** | **4.424198** |
| hypertension | | **.9308305** | **.0739865** | **-0.90** | **0.367** | **.7965507** | **1.087747** |
| dialysis | | **.5899024** | **.2309147** | **-1.35** | **0.178** | **.2738931** | **1.270514** |
| documented_history_of_cirrhosis | | **1.153082** | **.3518316** | **0.47** | **0.641** | **.6340725** | **2.096917** |
| metastasis | | **.2312374** | **.1353344** | **-2.50** | **0.012** | **.0734315** | **.7281714** |
| active_chemotherapy | | **.6253514** | **.4908292** | **-0.60** | **0.550** | **.1342853** | **2.912191** |
| obesity | | **1.377709** | **.1225639** | **3.60** | **0.000** | **1.157265** | **1.640145** |
| ascites | | **1.51581** | **1.416314** | **0.45** | **0.656** | **.2428371** | **9.461817** |
| drug_use | | **1.042497** | **.0767863** | **0.57** | **0.572** | **.902357** | **1.2044** |
| smoker | | **.9562327** | **.0582463** | **-0.73** | **0.463** | **.8486234** | **1.077487** |
| psych | | **.9928236** | **.1038936** | **-0.07** | **0.945** | **.8087206** | **1.218837** |
| ac_coag | | **1.061445** | **.1159667** | **0.55** | **0.585** | **.8568417** | **1.314905** |
| routine_steroid_use | | **1.695971** | **.3541571** | **2.53** | **0.011** | **1.126337** | **2.553692** |
| year_admit  2013 | | **.8976245** | **.1046225** | **-0.93** | **0.354** | **.7143039** | **1.127993** |
| 2014 | | **.8687971** | **.1154513** | **-1.06** | **0.290** | **.6695843** | **1.127279** |
| 2015 | | **1.007462** | **.1632979** | **0.05** | **0.963** | **.7332613** | **1.384199** |
| 2016 | | **.8683465** | **.1374928** | **-0.89** | **0.373** | **.6366708** | **1.184326** |
| 2017 | | **.8424916** | **.1275288** | **-1.13** | **0.258** | **.6262076** | **1.133477** |
| 2018 | | **.8396308** | **.1477741** | **-0.99** | **0.321** | **.5946724** | **1.185493** |
| _cons | | **.0981186** | **.0203077** | **-11.22** | **0.000** | **.0654** | **.147206** |
| Note: **_cons** estimates baseline odds. | |  |  |  |  |  |  |

(98,399 observations deleted)

# 7,742 100.00

| isscat | Freq. | Percent | Cum. |
| --- | --- | --- | --- |
| 25-35 | **7,742** | **100.00** | **100.00** |
| Total |  | | |

**comp_infection**

Iteration 0: log pseudolikelihood = **-3452.0678**

Iteration 1: log pseudolikelihood = **-3274.4609**

Iteration 2: log pseudolikelihood = **-3265.4034**

Iteration 3: log pseudolikelihood = **-3265.3627**

Iteration 4: log pseudolikelihood = **-3265.3627**

Logistic regression Number of obs = **7,742**

Wald chi2(33) = **.**

Prob > chi2 = **.**

Log pseudolikelihood = **-3265.3627** Pseudo R2 = **0.0541**

(Std. Err. adjusted for **35** clusters in traumactr)

comp_infection

diabetes

Robust

Odds Ratio Std. Err. z P>|z| [95% Conf. Interval]

# 1.133555 .1323382 1.07 0.283 .9017127 1.425008

agecat

| 26-45 | **1.163523** | **.1201689** | **1.47** | **0.143** | **.950304** | **1.424581** |
| --- | --- | --- | --- | --- | --- | --- |
| 46-65 | **1.240084** | **.1842951** | **1.45** | **0.148** | **.9267228** | **1.659405** |
| 66-75 | **1.009217** | **.1495113** | **0.06** | **0.951** | **.7548879** | **1.349231** |
| >75 | **.6357331** | **.089856** | **-3.20** | **0.001** | **.4819078** | **.8386595** |
| male | **1.276227** | **.077266** | **4.03** | **0.000** | **1.133429** | **1.437017** |
| aishn | **1.364338** | **.1160884** | **3.65** | **0.000** | **1.154769** | **1.61194** |
| aisfac | **.8728315** | **.1556099** | **-0.76** | **0.446** | **.6154273** | **1.237896** |
| aischs | **1.346301** | **.1117627** | **3.58** | **0.000** | **1.144142** | **1.584179** |
| aisabd | **1.277708** | **.0933512** | **3.35** | **0.001** | **1.107239** | **1.474421** |
| aisext | **1.297104** | **.1267858** | **2.66** | **0.008** | **1.070962** | **1.570998** |
| gcsmcat2  1 | **2.010483** | **.2015562** | **6.97** | **0.000** | **1.65183** | **2.447009** |
| 2 | **1.411608** | **.1394907** | **3.49** | **0.000** | **1.163057** | **1.713275** |
| 99 | **1.277383** | **.1683606** | **1.86** | **0.063** | **.9865789** | **1.653905** |
| pulse2 |  | | | | | |
| 1 | **1.275689** | **.1152764** | **2.69** | **0.007** | **1.068629** | **1.52287** |
| 2 | **.7419769** | **.1580677** | **-1.40** | **0.161** | **.488713** | **1.126489** |
| 99 | **.4058228** | **.1576819** | **-2.32** | **0.020** | **.1894974** | **.8690999** |
| bp2 1 | **1.43503** | **.1580443** | **3.28** | **0.001** | **1.15642** | **1.780764** |
| 2 | **.6943334** | **.1840998** | **-1.38** | **0.169** | **.412929** | **1.167511** |
| 99 | **1.156874** | **.3083754** | **0.55** | **0.585** | **.6861052** | **1.950661** |
| race | **1.296365** | **.094436** | **3.56** | **0.000** | **1.12388** | **1.495321** |
| blunt_ind | **1.315678** | **.1877243** | **1.92** | **0.055** | **.9947136** | **1.740208** |
| transfer | **1.341913** | **.1706476** | **2.31** | **0.021** | **1.045874** | **1.721748** |
| congestive_heart_failure | **.9644299** | **.2325941** | **-0.15** | **0.881** | **.6011519** | **1.547238** |
| pvd | **1.286916** | **.7176521** | **0.45** | **0.651** | **.4313955** | **3.839061** |
| hypertension | **1.046597** | **.0773176** | **0.62** | **0.538** | **.9055169** | **1.209657** |
| dialysis | **.4916985** | **.1668169** | **-2.09** | **0.036** | **.2528801** | **.9560555** |
| documented_history_of_cirrhosis | **1.147232** | **.2966284** | **0.53** | **0.595** | **.6911377** | **1.904312** |
| metastasis | **.4604974** | **.2774054** | **-1.29** | **0.198** | **.1414035** | **1.499665** |
| active_chemotherapy | **.8843755** | **.8819557** | **-0.12** | **0.902** | **.1252461** | **6.244666** |
| obesity | **1.261204** | **.1659651** | **1.76** | **0.078** | **.9744814** | **1.63229** |

| ascites | **2.398855** | **1.972666** | **1.06** | **0.287** | **.4786631** | **12.02204** |
| --- | --- | --- | --- | --- | --- | --- |
| drug_use | **1.030816** | **.0969816** | **0.32** | **0.747** | **.8572312** | **1.23955** |
| smoker | **1.219411** | **.1024579** | **2.36** | **0.018** | **1.034261** | **1.437706** |
| psych | **1.022572** | **.0804772** | **0.28** | **0.777** | **.8764024** | **1.19312** |
| ac_coag | **1.089468** | **.1218755** | **0.77** | **0.444** | **.8749697** | **1.35655** |
| routine_steroid_use | **1.038433** | **.3421245** | **0.11** | **0.909** | **.5444266** | **1.980693** |
| year_admit  2013 | **.9096419** | **.0970396** | **-0.89** | **0.375** | **.738015** | **1.121181** |
| 2014 | **.8423752** | **.1502324** | **-0.96** | **0.336** | **.5938805** | **1.194846** |
| 2015 | **.8974408** | **.168365** | **-0.58** | **0.564** | **.6213204** | **1.296272** |
| 2016 | **.6138503** | **.1004216** | **-2.98** | **0.003** | **.4454632** | **.8458885** |
| 2017 | **.5632582** | **.0863627** | **-3.74** | **0.000** | **.4170569** | **.7607111** |
| 2018 | **.5469956** | **.0967829** | **-3.41** | **0.001** | **.3867017** | **.7737338** |
| _cons | **.0646207** | **.0156466** | **-11.31** | **0.000** | **.0402041** | **.1038659** |

Note: **_cons** estimates baseline odds.

(98,399 observations deleted)

| isscat | Freq. | Percent | Cum. |
| --- | --- | --- | --- |
| 25-35 | **7,742** | **100.00** | **100.00** |
| Total | **7,742** | **100.00** |  |

# comp_cardiac

note: metastasis != 0 predicts failure perfectly metastasis dropped and 31 obs not used

note: active_chemotherapy != 0 predicts failure perfectly active_chemotherapy dropped and 23 obs not used

note: ascites != 0 predicts failure perfectly ascites dropped and 5 obs not used

Iteration 0: log pseudolikelihood = **-1536.5085**

Iteration 1: log pseudolikelihood = **-1446.2706**

Iteration 2: log pseudolikelihood = **-1366.4937**

Iteration 3: log pseudolikelihood = **-1365.9688**

Iteration 4: log pseudolikelihood = **-1365.9683**

Iteration 5: log pseudolikelihood = **-1365.9683**

Logistic regression Number of obs = **7,683**

Wald chi2(33) = **.**

Prob > chi2 = **.**

Log pseudolikelihood = **-1365.9683** Pseudo R2 = **0.1110**

(Std. Err. adjusted for **35** clusters in traumactr)

comp_cardiac

diabetes

Robust

Odds Ratio Std. Err. z P>|z| [95% Conf. Interval]

# 1.657692 .2743072 3.05 0.002 1.198535 2.29275

| agecat  26-45 | **1.764905** | **.2819082** | **3.56** | **0.000** | **1.290503** | **2.413703** |
| --- | --- | --- | --- | --- | --- | --- |
| 46-65 | **2.476489** | **.3838363** | **5.85** | **0.000** | **1.827709** | **3.355568** |
| 66-75 | **1.905321** | **.5255519** | **2.34** | **0.019** | **1.10963** | **3.271586** |
| >75 | **2.060083** | **.5123634** | **2.91** | **0.004** | **1.265268** | **3.354186** |
| male | **1.207506** | **.150396** | **1.51** | **0.130** | **.9459568** | **1.541371** |
| aishn | **.5982296** | **.0919253** | **-3.34** | **0.001** | **.44266** | **.8084728** |
| aisfac | **.4610862** | **.2040409** | **-1.75** | **0.080** | **.1936897** | **1.097634** |

| aischs | **1.124352** | **.1811449** | **0.73** | **0.467** | **.8199078** | **1.541842** |
| --- | --- | --- | --- | --- | --- | --- |
| aisabd | **.8718125** | **.1302522** | **-0.92** | **0.359** | **.6505044** | **1.168412** |
| aisext | **1.244845** | **.187332** | **1.46** | **0.146** | **.9268755** | **1.671897** |
| gcsmcat2  1 | **2.593674** | **.3370742** | **7.33** | **0.000** | **2.01045** | **3.346091** |
| 2 | **3.57238** | **.4560707** | **9.97** | **0.000** | **2.781559** | **4.588038** |
| 99 | **2.34926** | **.6625001** | **3.03** | **0.002** | **1.351728** | **4.082941** |
| pulse2 |  |  |  |  |  |  |
| 1 | **1.842136** | **.235979** | **4.77** | **0.000** | **1.433118** | **2.367888** |
| 2 | **1.414457** | **.4645636** | **1.06** | **0.291** | **.7430561** | **2.692514** |
| 99 | **.9863498** | **.4652801** | **-0.03** | **0.977** | **.3912938** | **2.486331** |
| bp2 1 | **1.769116** | **.2936755** | **3.44** | **0.001** | **1.277779** | **2.449384** |
| 2 | **1.932398** | **.5927722** | **2.15** | **0.032** | **1.05922** | **3.525386** |
| 99 | **.9875824** | **.5382713** | **-0.02** | **0.982** | **.3393399** | **2.874165** |
| race | **1.082537** | **.1580252** | **0.54** | **0.587** | **.8131811** | **1.441114** |
| blunt_ind | **.4007784** | **.057971** | **-6.32** | **0.000** | **.3018433** | **.5321416** |
| transfer | **.908303** | **.1293411** | **-0.68** | **0.499** | **.6871014** | **1.200717** |
| congestive_heart_failure | **.644382** | **.2908491** | **-0.97** | **0.330** | **.2660381** | **1.560784** |
| pvd | **2.214982** | **1.119303** | **1.57** | **0.116** | **.8226783** | **5.963624** |
| hypertension | **.7378709** | **.1068135** | **-2.10** | **0.036** | **.5555988** | **.9799401** |
| dialysis | **.9956254** | **.6663177** | **-0.01** | **0.995** | **.2681843** | **3.696227** |
| documented_history_of_cirrhosis | **1.728565** | **.83409** | **1.13** | **0.257** | **.6713555** | **4.450602** |
| metastasis | **1** | (omitted) |  |  |  |  |
| active_chemotherapy | **1** | (omitted) |  |  |  |  |
| obesity | **.7457219** | **.159216** | **-1.37** | **0.169** | **.4907275** | **1.133218** |
| ascites | **1** | (omitted) |  |  |  |  |
| drug_use | **.8815835** | **.1651753** | **-0.67** | **0.501** | **.6106334** | **1.272759** |
| smoker | **.6163002** | **.1053804** | **-2.83** | **0.005** | **.4408049** | **.8616644** |
| psych | **1.016317** | **.1680432** | **0.10** | **0.922** | **.7349998** | **1.405308** |
| ac_coag | **1.279186** | **.1907343** | **1.65** | **0.099** | **.9550243** | **1.713377** |
| routine_steroid_use | **1.115014** | **.8096095** | **0.15** | **0.881** | **.2686731** | **4.627391** |
| year_admit  2013 | **.7827675** | **.2138294** | **-0.90** | **0.370** | **.4582571** | **1.337077** |
| 2014 | **.8390432** | **.2243782** | **-0.66** | **0.512** | **.4967693** | **1.417144** |
| 2015 | **1.274691** | **.3072426** | **1.01** | **0.314** | **.7947624** | **2.044432** |
| 2016 | **.9300349** | **.2407218** | **-0.28** | **0.779** | **.5599921** | **1.544602** |
| 2017 | **1.206361** | **.2409668** | **0.94** | **0.348** | **.8155535** | **1.784441** |
| 2018 | **1.267079** | **.3002658** | **1.00** | **0.318** | **.7963247** | **2.016123** |
| _cons | **.029995** | **.0108345** | **-9.71** | **0.000** | **.014777** | **.0608851** |
| Note: **_cons** estimates baseline odds. |  |  |  |  |  |  |

94 .

1. . **For ISS >35
2. . outcomes_iss 3

(104,128 observations deleted)

| isscat | Freq. | Percent | Cum. |
| --- | --- | --- | --- |
| >35 | **2,013** | **100.00** | **100.00** |
| Total | **2,013** | **100.00** |  |

# dead

note: metastasis != 0 predicts success perfectly metastasis dropped and 2 obs not used

note: active_chemotherapy != 0 predicts success perfectly active_chemotherapy dropped and 1 obs not used

note: ascites != 0 predicts success perfectly ascites dropped and 2 obs not used

Iteration 0: log pseudolikelihood = **-1370.533**

Iteration 1: log pseudolikelihood = **-1001.3405**

Iteration 2: log pseudolikelihood = **-996.44591**

Iteration 3: log pseudolikelihood = **-996.43727**

Iteration 4: log pseudolikelihood = **-996.43727**

| Logistic regression  Log pseudolikelihood | = | Number of obs Wald chi2(33) Prob > chi2  **-996.43727** Pseudo R2 | | | | =  =  =  = | **2,008**  **.**  **. 0.2730** | | |
| --- | --- | --- | --- | --- | --- | --- | --- | --- | --- |
| (Std. Err. adjusted for **35** clusters in traumactr) | | | | | | | | | |
| dead | | | Odds Ratio | Robust Std. Err. | z | P>\|z\| | | [95% Conf. | Interval] |
| diabetes | | | **1.005414** | **.3026553** | **0.02** | **0.986** | | **.5573288** | **1.813754** |
| agecat 26-45 | | | **1.082738** | **.1137732** | **0.76** | **0.449** | | **.8812113** | **1.330354** |
| 46-65 | | | **1.702081** | **.3035928** | **2.98** | **0.003** | | **1.199928** | **2.414379** |
| 66-75 | | | **4.931241** | **1.368564** | **5.75** | **0.000** | | **2.862351** | **8.495512** |
| >75 | | | **11.74599** | **2.258016** | **12.81** | **0.000** | | **8.058557** | **17.12073** |
| male | | | **1.378259** | **.1629523** | **2.71** | **0.007** | | **1.093183** | **1.737676** |
| aishn | | | **1.218393** | **.2224443** | **1.08** | **0.279** | | **.8518865** | **1.74258** |
| aisfac | | | **.7980659** | **.1790585** | **-1.01** | **0.315** | | **.5141128** | **1.238851** |
| aischs | | | **1.412091** | **.2876995** | **1.69** | **0.090** | | **.9471937** | **2.105167** |
| aisabd | | | **.9276237** | **.1050648** | **-0.66** | **0.507** | | **.7429555** | **1.158193** |
| aisext | | | **.9460873** | **.1102069** | **-0.48** | **0.634** | | **.7529693** | **1.188735** |
| gcsmcat2  1 | | | **2.799911** | **.4169037** | **6.91** | **0.000** | | **2.091227** | **3.748756** |
| 2 | | | **11.11961** | **1.364108** | **19.63** | **0.000** | | **8.743146** | **14.14202** |
| 99 | | | **3.977337** | **1.359741** | **4.04** | **0.000** | | **2.035121** | **7.773105** |
| pulse2 | | |  | | | | | | |
| 1 | | | **1.689914** | **.2018536** | **4.39** | **0.000** | | **1.337186** | **2.135685** |
| 2 | | | **2.500542** | **.7736514** | **2.96** | **0.003** | | **1.363572** | **4.585539** |
| 99 | | | **3.703935** | **1.305596** | **3.71** | **0.000** | | **1.856198** | **7.390988** |
| bp2 1 | | | **1.384318** | **.2220987** | **2.03** | **0.043** | | **1.010811** | **1.895841** |
| 2 | | | **3.168445** | **1.060002** | **3.45** | **0.001** | | **1.644664** | **6.104011** |
| 99 | | | **1.722657** | **.6040811** | **1.55** | **0.121** | | **.8663823** | **3.425218** |
| race | | | **1.045576** | **.1192249** | **0.39** | **0.696** | | **.8361708** | **1.307424** |
| blunt_ind | | | **.5572371** | **.1057955** | **-3.08** | **0.002** | | **.3840903** | **.8084381** |
| transfer | | | **.4368167** | **.0854451** | **-4.23** | **0.000** | | **.2977124** | **.6409168** |
| congestive_heart_failure | | | **1.206159** | **.8826191** | **0.26** | **0.798** | | **.2874279** | **5.061508** |
| pvd | | | **1.052926** | **.8795016** | **0.06** | **0.951** | | **.2048309** | **5.412531** |
| hypertension | | | **.6312065** | **.1302719** | **-2.23** | **0.026** | | **.4212073** | **.9459041** |
| dialysis | | | **.6613219** | **.6370899** | **-0.43** | **0.668** | | **.1000924** | **4.369431** |
| documented_history_of_cirrhosis | | | **3.575237** | **4.048545** | **1.13** | **0.261** | | **.3885231** | **32.89977** |
| metastasis | | | **1** | (omitted) |  |  | |  |  |
| active_chemotherapy | | | **1** | (omitted) |  |  | |  |  |
| obesity | | | **1.083998** | **.3133553** | **0.28** | **0.780** | | **.6151319** | **1.910242** |
| ascites | | | **1** | (omitted) |  |  | |  |  |
| drug_use | | | **.4528682** | **.0601818** | **-5.96** | **0.000** | | **.3490241** | **.5876087** |
| smoker | | | **.6293119** | **.0654486** | **-4.45** | **0.000** | | **.5132639** | **.771598** |
| psych | | | **1.07193** | **.1475389** | **0.50** | **0.614** | | **.8184806** | **1.403863** |

|  | | ac_coag |  | **.7251681** | **.2205508** | **-1.06** | **0.291** | **.3995365** | **1.316197** |
| --- | --- | --- | --- | --- | --- | --- | --- | --- | --- |
|  |  | routine_steroid_use |  | **.4043702** | **.4667384** | **-0.78** | **0.433** | **.042101** | **3.883882** |
|  |  | year_admit  2013 |  | **.818211** | **.1868783** | **-0.88** | **0.380** | **.5229402** | **1.280202** |
|  |  | 2014 |  | **.6822154** | **.1109327** | **-2.35** | **0.019** | **.4960331** | **.93828** |
|  |  | 2015 |  | **.863079** | **.1852314** | **-0.69** | **0.493** | **.5667197** | **1.314416** |
|  |  | 2016 |  | **.7827298** | **.1157116** | **-1.66** | **0.098** | **.5858388** | **1.045793** |
|  |  | 2017 |  | **.9044655** | **.1464559** | **-0.62** | **0.535** | **.6585077** | **1.24229** |
|  |  | 2018 |  | **.729157** | **.1267648** | **-1.82** | **0.069** | **.5186074** | **1.025188** |
|  |  | _cons |  | **.1254583** | **.0444903** | **-5.85** | **0.000** | **.0626102** | **.2513935** |
| Note: | **_cons** | estimates baseline | odds. |  |  |  |  |  |  |

(104,128 observations deleted)

| isscat | Freq. | Percent | Cum. |
| --- | --- | --- | --- |
| >35 | **2,013** | **100.00** | **100.00** |
| Total | **2,013** | **100.00** |  |

# comp_any

note: metastasis != 0 predicts failure perfectly metastasis dropped and 2 obs not used

note: active_chemotherapy != 0 predicts success perfectly active_chemotherapy dropped and 1 obs not used

note: ascites != 0 predicts failure perfectly ascites dropped and 2 obs not used

Iteration 0: log pseudolikelihood = **-1373.3605**

Iteration 1: log pseudolikelihood = **-1316.6174**

Iteration 2: log pseudolikelihood = **-1316.2766**

Iteration 3: log pseudolikelihood = **-1316.2765**

| Logistic regression  Log pseudolikelihood | Number of obs Wald chi2(33) Prob > chi2  = **-1316.2765** Pseudo R2 | | | | =  =  =  = | **2,008**  **.**  **. 0.0416** | | |
| --- | --- | --- | --- | --- | --- | --- | --- | --- |
|  | |  | (Std. Err. | adjusted | | for **35** | clusters in | traumactr) |
| comp_any | | Odds Ratio | Robust Std. Err. | z | | P>\|z\| | [95% Conf. | Interval] |
| diabetes | | **1.148785** | **.2282376** | **0.70** | | **0.485** | **.7782588** | **1.695716** |
| agecat 26-45 | | **1.077799** | **.1539198** | **0.52** | | **0.600** | **.8146633** | **1.425927** |
| 46-65 | | **1.142453** | **.1717533** | **0.89** | | **0.376** | **.8508854** | **1.53393** |
| 66-75 | | **1.149246** | **.2977807** | **0.54** | | **0.591** | **.6916053** | **1.90971** |
| >75 | | **.8794795** | **.1563725** | **-0.72** | | **0.470** | **.620699** | **1.24615** |
| male | | **1.130162** | **.1184612** | **1.17** | | **0.243** | **.9202788** | **1.387911** |
| aishn | | **1.153701** | **.120764** | **1.37** | | **0.172** | **.9397093** | **1.416423** |
| aisfac | | **1.09521** | **.24772** | **0.40** | | **0.688** | **.7030211** | **1.706186** |
| aischs | | **1.260178** | **.116479** | **2.50** | | **0.012** | **1.051368** | **1.510459** |
| aisabd | | **1.190515** | **.1079558** | **1.92** | | **0.054** | **.9966624** | **1.422073** |
| aisext | | **1.468293** | **.1327852** | **4.25** | | **0.000** | **1.2298** | **1.753038** |
| gcsmcat2  1 | | **1.518649** | **.1678453** | **3.78** | | **0.000** | **1.22287** | **1.885969** |

| 2 | **1.086358** | | **.1936434** | **0.46** | **0.642** | **.7660306** | **1.540635** |
| --- | --- | --- | --- | --- | --- | --- | --- |
| 99 | **1.307261** | | **.4152599** | **0.84** | **0.399** | **.7014113** | **2.436419** |
| pulse2 |  | |  |  |  |  |  |
| 1 |  | **1.154672** | **.1160755** | **1.43** | **0.153** | **.9481786** | **1.406136** |
| 2 |  | **1.216008** | **.3368448** | **0.71** | **0.480** | **.706555** | **2.092794** |
| 99 |  | **.2463087** | **.0673739** | **-5.12** | **0.000** | **.1440943** | **.4210297** |
| bp2 1 |  | **1.343912** | **.1659822** | **2.39** | **0.017** | **1.054974** | **1.711985** |
| 2 |  | **.7913238** | **.1903975** | **-0.97** | **0.331** | **.4937984** | **1.268115** |
| 99 |  | **1.550407** | **.4151733** | **1.64** | **0.102** | **.917293** | **2.620496** |
| race |  | **1.350145** | **.1964188** | **2.06** | **0.039** | **1.015192** | **1.795614** |
| blunt_ind |  | **1.252248** | **.3222044** | **0.87** | **0.382** | **.7562676** | **2.073505** |
| transfer |  | **1.308619** | **.2005769** | **1.75** | **0.079** | **.9690509** | **1.767177** |
| congestive_heart_failure |  | **1.006904** | **.5544006** | **0.01** | **0.990** | **.3422295** | **2.962504** |
| pvd |  | **6.09828** | **5.066325** | **2.18** | **0.030** | **1.196869** | **31.07192** |
| hypertension |  | **1.020783** | **.1100187** | **0.19** | **0.849** | **.8264029** | **1.260883** |
| dialysis |  | **1.113471** | **.9315062** | **0.13** | **0.898** | **.2160635** | **5.738206** |
| documented_history_of_cirrhosis |  | **1.612342** | **.9071214** | **0.85** | **0.396** | **.5352564** | **4.856826** |
| metastasis |  | **1** | (omitted) |  |  |  |  |
| active_chemotherapy |  | **1** | (omitted) |  |  |  |  |
| obesity |  | **1.944212** | **.3254548** | **3.97** | **0.000** | **1.40041** | **2.699181** |
| ascites |  | **1** | (omitted) |  |  |  |  |
| drug_use |  | **.9928251** | **.1233864** | **-0.06** | **0.954** | **.7781926** | **1.266655** |
| smoker |  | **1.163223** | **.1083414** | **1.62** | **0.105** | **.9691323** | **1.396185** |
| psych |  | **.9543731** | **.1208183** | **-0.37** | **0.712** | **.7446648** | **1.223138** |
| ac_coag |  | **1.6606** | **.5984867** | **1.41** | **0.159** | **.819386** | **3.365436** |
| routine_steroid_use |  | **3.116437** | **2.538876** | **1.40** | **0.163** | **.6312592** | **15.38541** |
| year_admit  2013 |  | **.9186139** | **.1780745** | **-0.44** | **0.661** | **.6282413** | **1.343197** |
| 2014 |  | **.7378457** | **.1286778** | **-1.74** | **0.081** | **.5242264** | **1.038514** |
| 2015 |  | **.8263339** | **.1402056** | **-1.12** | **0.261** | **.5925577** | **1.15234** |
| 2016 |  | **.8734095** | **.1671369** | **-0.71** | **0.479** | **.600248** | **1.270882** |
| 2017 |  | **.9482** | **.1450368** | **-0.35** | **0.728** | **.7025869** | **1.279676** |
| 2018 |  | **.9504955** | **.1675032** | **-0.29** | **0.773** | **.6728918** | **1.342626** |
| _cons |  | **.2309603** | **.0690455** | **-4.90** | **0.000** | **.1285497** | **.4149575** |
| Note: **_cons** estimates baseline | odds. |  |  |  |  |  |  |
| (104,128 observations deleted) |  |  |  |  |  |  |  |

| isscat | Freq. | Percent | Cum. |
| --- | --- | --- | --- |
| >35 | **2,013** | **100.00** | **100.00** |
| Total | **2,013** | **100.00** |  |

# comp_sev

note: metastasis != 0 predicts failure perfectly metastasis dropped and 2 obs not used

note: active_chemotherapy != 0 predicts success perfectly active_chemotherapy dropped and 1 obs not used

note: ascites != 0 predicts failure perfectly ascites dropped and 2 obs not used

Iteration 0: log pseudolikelihood = **-1357.9271**

Iteration 1: log pseudolikelihood = **-1300.8248**

Iteration 2: log pseudolikelihood = **-1300.3759**

Iteration 3: log pseudolikelihood = **-1300.3758**

Iteration 4: log pseudolikelihood = **-1300.3758**

Logistic regression Number of obs = **2,008**

Wald chi2(33) = **.**

Prob > chi2 = **.**

Log pseudolikelihood = **-1300.3758** Pseudo R2 = **0.0424**

(Std. Err. adjusted for **35** clusters in traumactr)

| comp_sev diabetes agecat | Robust  Odds Ratio Std. Err. z P>\|z\| [95% Conf. Interval]  **1.138307 .2379357 0.62 0.535 .7556771 1.714678** | | | | | |
| --- | --- | --- | --- | --- | --- | --- |
| 26-45 | **1.16637** | **.1664523** | **1.08** | **0.281** | **.8817827** | **1.542805** |
| 46-65 | **1.196879** | **.1784567** | **1.21** | **0.228** | **.8935827** | **1.603119** |
| 66-75 | **1.28717** | **.3420987** | **0.95** | **0.342** | **.7645524** | **2.167029** |
| >75 | **.9307689** | **.1704673** | **-0.39** | **0.695** | **.6500504** | **1.332713** |
| male | **1.23129** | **.1266178** | **2.02** | **0.043** | **1.006534** | **1.506233** |
| aishn | **1.178581** | **.123884** | **1.56** | **0.118** | **.9591515** | **1.44821** |
| aisfac | **1.131054** | **.2834315** | **0.49** | **0.623** | **.6921181** | **1.848359** |
| aischs | **1.282888** | **.1245136** | **2.57** | **0.010** | **1.060653** | **1.551687** |
| aisabd | **1.140857** | **.1063063** | **1.41** | **0.157** | **.9504198** | **1.369453** |
| aisext | **1.403099** | **.1157773** | **4.10** | **0.000** | **1.193578** | **1.649398** |
| gcsmcat2  1 | **1.583484** | **.1587955** | **4.58** | **0.000** | **1.300928** | **1.92741** |
| 2 | **1.132493** | **.2123577** | **0.66** | **0.507** | **.784194** | **1.635487** |
| 99 | **1.340005** | **.4070678** | **0.96** | **0.335** | **.7388017** | **2.430441** |
| pulse2 |  | | | | | |
| 1 | **1.177595** | **.1075202** | **1.79** | **0.073** | **.9846394** | **1.408364** |
| 2 | **1.222238** | **.3215179** | **0.76** | **0.446** | **.7298631** | **2.046775** |
| 99 | **.2575912** | **.0859378** | **-4.07** | **0.000** | **.1339529** | **.4953471** |
| bp2 1 | **1.315485** | **.1604644** | **2.25** | **0.025** | **1.03575** | **1.670769** |
| 2 | **.8286574** | **.2018105** | **-0.77** | **0.440** | **.5141314** | **1.335598** |
| 99 | **1.550471** | **.4571795** | **1.49** | **0.137** | **.8699096** | **2.763459** |
| race | **1.325551** | **.2061445** | **1.81** | **0.070** | **.9772842** | **1.797927** |
| blunt_ind | **1.276439** | **.3119707** | **1.00** | **0.318** | **.7906063** | **2.060818** |
| transfer | **1.26639** | **.1791827** | **1.67** | **0.095** | **.9596881** | **1.671109** |
| congestive_heart_failure | **.8832652** | **.4320476** | **-0.25** | **0.800** | **.3386316** | **2.303853** |
| pvd | **6.540103** | **5.279787** | **2.33** | **0.020** | **1.344047** | **31.82399** |
| hypertension | **.9681747** | **.1081738** | **-0.29** | **0.772** | **.7777667** | **1.205197** |
| dialysis | **1.272164** | **1.07692** | **0.28** | **0.776** | **.2420913** | **6.685091** |
| documented_history_of_cirrhosis | **1.821525** | **1.019332** | **1.07** | **0.284** | **.6082746** | **5.454696** |
| metastasis | **1** | (omitted) |  |  |  |  |
| active_chemotherapy | **1** | (omitted) |  |  |  |  |
| obesity | **2.080479** | **.3312002** | **4.60** | **0.000** | **1.52285** | **2.842299** |
| ascites | **1** | (omitted) |  |  |  |  |
| drug_use | **1.037012** | **.1199468** | **0.31** | **0.753** | **.826664** | **1.300885** |
| smoker | **1.065079** | **.1025439** | **0.65** | **0.513** | **.8819208** | **1.286275** |
| psych | **1.059578** | **.1371938** | **0.45** | **0.655** | **.8220904** | **1.365671** |
| ac_coag | **1.738725** | **.6185554** | **1.55** | **0.120** | **.8657928** | **3.491787** |
| routine_steroid_use | **2.222844** | **1.481848** | **1.20** | **0.231** | **.6018104** | **8.210284** |
| year_admit  2013 | **.8443785** | **.144959** | **-0.99** | **0.324** | **.6031243** | **1.182136** |
| 2014 | **.6643085** | **.1396509** | **-1.95** | **0.052** | **.4399772** | **1.00302** |

| 2015 | **.7408022** | **.1223614** | **-1.82** | **0.069** | **.5359271** | **1.023997** |
| --- | --- | --- | --- | --- | --- | --- |
| 2016 | **.8345932** | **.1580115** | **-0.96** | **0.340** | **.5758626** | **1.209569** |
| 2017 | **.8892761** | **.1404185** | **-0.74** | **0.457** | **.6525746** | **1.211834** |
| 2018 | **.9409379** | **.1687046** | **-0.34** | **0.734** | **.6621335** | **1.337139** |
| _cons | **.1909046** | **.0541283** | **-5.84** | **0.000** | **.1095141** | **.3327841** |

Note: **_cons** estimates baseline odds.

(104,128 observations deleted)

# 2,013 100.00

| isscat | Freq. | Percent | Cum. |
| --- | --- | --- | --- |
| >35 | **2,013** | **100.00** | **100.00** |
| Total |  | | |

**comp_infection**

note: metastasis != 0 predicts failure perfectly metastasis dropped and 2 obs not used

note: active_chemotherapy != 0 predicts failure perfectly active_chemotherapy dropped and 1 obs not used

note: ascites != 0 predicts failure perfectly ascites dropped and 2 obs not used

Iteration 0: log pseudolikelihood = **-1147.4644**

Iteration 1: log pseudolikelihood = **-1098.298**

Iteration 2: log pseudolikelihood = **-1096.1609**

Iteration 3: log pseudolikelihood = **-1096.1483**

Iteration 4: log pseudolikelihood = **-1096.1483**

| Logistic regression  Log pseudolikelihood | = | Number of obs Wald chi2(33) Prob > chi2  **-1096.1483** Pseudo R2 | | | | =  =  =  = | **2,008**  **.**  **. 0.0447** | | |
| --- | --- | --- | --- | --- | --- | --- | --- | --- | --- |
|  | | |  | (Std. Err. | adjusted | | for **35** | clusters in | traumactr) |
| comp_infection | | | Odds Ratio | Robust Std. Err. | z | | P>\|z\| | [95% Conf. | Interval] |
| diabetes | | | **1.254773** | **.247168** | **1.15** | | **0.249** | **.8528907** | **1.846021** |
| agecat 26-45 | | | **.9281349** | **.1474357** | **-0.47** | | **0.639** | **.6798239** | **1.267143** |
| 46-65 | | | **1.007554** | **.1692093** | **0.04** | | **0.964** | **.7249646** | **1.400295** |
| 66-75 | | | **.7435011** | **.168464** | **-1.31** | | **0.191** | **.4768861** | **1.159174** |
| >75 | | | **.4966108** | **.1177051** | **-2.95** | | **0.003** | **.3120809** | **.7902513** |
| male | | | **1.021921** | **.1083018** | **0.20** | | **0.838** | **.830248** | **1.257843** |
| aishn | | | **1.272459** | **.1401886** | **2.19** | | **0.029** | **1.025335** | **1.579144** |
| aisfac | | | **1.134391** | **.2973051** | **0.48** | | **0.630** | **.6786985** | **1.896045** |
| aischs | | | **.996334** | **.127371** | **-0.03** | | **0.977** | **.7755104** | **1.280036** |
| aisabd | | | **1.084576** | **.1141158** | **0.77** | | **0.440** | **.8824686** | **1.332972** |
| aisext | | | **1.15351** | **.1283088** | **1.28** | | **0.199** | **.9275547** | **1.43451** |
| gcsmcat2  1 | | | **1.423061** | **.1801361** | **2.79** | | **0.005** | **1.110389** | **1.823776** |
| 2 | | | **.9080812** | **.1459011** | **-0.60** | | **0.548** | **.6627694** | **1.244191** |
| 99 | | | **1.293592** | **.4618682** | **0.72** | | **0.471** | **.6425124** | **2.604432** |
| pulse2  1 | | | **.9608994 .1221541 -0.31 0.754 .7489782 1.232783** | | | | | | |

| 2 |  | **.7042681** | **.167936** | **-1.47** | **0.141** | **.4413317** | **1.123857** |
| --- | --- | --- | --- | --- | --- | --- | --- |
| 99 |  | **.2694769** | **.0893818** | **-3.95** | **0.000** | **.1406661** | **.5162422** |
| bp2 1 |  | **1.033998** | **.1312236** | **0.26** | **0.792** | **.8062959** | **1.326003** |
| 2 |  | **.605713** | **.2162065** | **-1.40** | **0.160** | **.3009084** | **1.219269** |
| 99 |  | **1.009475** | **.2434952** | **0.04** | **0.969** | **.6291841** | **1.619622** |
| race |  | **1.292576** | **.1892097** | **1.75** | **0.080** | **.9701873** | **1.722093** |
| blunt_ind |  | **1.354451** | **.332822** | **1.23** | **0.217** | **.836762** | **2.192424** |
| transfer |  | **1.186234** | **.2342528** | **0.86** | **0.387** | **.8055239** | **1.746877** |
| congestive_heart_failure |  | **.8577037** | **.5409628** | **-0.24** | **0.808** | **.2491583** | **2.952563** |
| pvd |  | **1.322764** | **1.158408** | **0.32** | **0.749** | **.2377082** | **7.360721** |
| hypertension |  | **1.158102** | **.1713282** | **0.99** | **0.321** | **.8666045** | **1.547649** |
| dialysis |  | **1.044738** | **.7862599** | **0.06** | **0.954** | **.2390018** | **4.566817** |
| documented_history_of_cirrhosis |  | **.1345115** | **.1619229** | **-1.67** | **0.096** | **.0127085** | **1.423715** |
| metastasis |  | **1** | (omitted) |  |  |  |  |
| active_chemotherapy |  | **1** | (omitted) |  |  |  |  |
| obesity |  | **2.128613** | **.5608124** | **2.87** | **0.004** | **1.270094** | **3.567446** |
| ascites |  | **1** | (omitted) |  |  |  |  |
| drug_use |  | **1.090085** | **.1731099** | **0.54** | **0.587** | **.7985207** | **1.488109** |
| smoker |  | **1.278684** | **.1829032** | **1.72** | **0.086** | **.9660671** | **1.692464** |
| psych |  | **1.251423** | **.1799442** | **1.56** | **0.119** | **.9440794** | **1.658822** |
| ac_coag |  | **1.336592** | **.478168** | **0.81** | **0.417** | **.6629493** | **2.694745** |
| routine_steroid_use |  | **2.280802** | **1.510119** | **1.25** | **0.213** | **.6230271** | **8.349646** |
| year_admit  2013 |  | **1.142967** | **.2449145** | **0.62** | **0.533** | **.750998** | **1.739518** |
| 2014 |  | **.9654641** | **.1812793** | **-0.19** | **0.852** | **.6682073** | **1.394958** |
| 2015 |  | **.8584998** | **.1732205** | **-0.76** | **0.450** | **.5780858** | **1.274935** |
| 2016 |  | **.8543324** | **.1652054** | **-0.81** | **0.416** | **.5848263** | **1.248035** |
| 2017 |  | **.8672653** | **.1402325** | **-0.88** | **0.378** | **.6317088** | **1.190658** |
| 2018 |  | **.6907548** | **.092879** | **-2.75** | **0.006** | **.530727** | **.899035** |
| _cons |  | **.1859474** | **.065248** | **-4.79** | **0.000** | **.0934776** | **.3698899** |
| Note: **_cons** estimates baseline | odds. |  |  |  |  |  |  |
| (104,128 observations deleted) |  |  |  |  |  |  |  |

| isscat | Freq. | Percent | Cum. |
| --- | --- | --- | --- |
| >35 | **2,013** | **100.00** | **100.00** |
| Total | **2,013** | **100.00** |  |

# comp_cardiac

note: metastasis != 0 predicts failure perfectly metastasis dropped and 2 obs not used

note: active_chemotherapy != 0 predicts success perfectly active_chemotherapy dropped and 1 obs not used

note: ascites != 0 predicts failure perfectly ascites dropped and 2 obs not used

Iteration 0: log pseudolikelihood = **-754.60629**

Iteration 1: log pseudolikelihood = **-698.01634**

Iteration 2: log pseudolikelihood = **-689.32139**

Iteration 3: log pseudolikelihood = **-689.15344**

Iteration 4: log pseudolikelihood = **-689.15286**

Iteration 5: log pseudolikelihood = **-689.15286**

| Logistic regression  Log pseudolikelihood | Number of obs Wald chi2(33) Prob > chi2  = **-689.15286** Pseudo R2 | | | | =  =  =  = | **2,008**  **.**  **. 0.0867** | | |
| --- | --- | --- | --- | --- | --- | --- | --- | --- |
| (Std. Err. adjusted for **35** clusters in traumactr) | | | | | | | | |
| comp_cardiac | | Odds Ratio | Robust Std. Err. | z | P>\|z\| | | [95% Conf. | Interval] |
| diabetes | | **1.066125** | **.3098631** | **0.22** | **0.826** | | **.6031303** | **1.884538** |
| agecat 26-45 | | **1.113228** | **.1829474** | **0.65** | **0.514** | | **.8066735** | **1.536279** |
| 46-65 | | **1.54363** | **.3230211** | **2.07** | **0.038** | | **1.024283** | **2.326303** |
| 66-75 | | **1.920459** | **.681422** | **1.84** | **0.066** | | **.9580309** | **3.849732** |
| >75 | | **2.373874** | **.6833533** | **3.00** | **0.003** | | **1.350291** | **4.173382** |
| male | | **1.01211** | **.2033965** | **0.06** | **0.952** | | **.6826025** | **1.500678** |
| aishn | | **.71802** | **.1121363** | **-2.12** | **0.034** | | **.5286895** | **.9751521** |
| aisfac | | **.7952629** | **.3417133** | **-0.53** | **0.594** | | **.3425794** | **1.846121** |
| aischs | | **1.150926** | **.1964517** | **0.82** | **0.410** | | **.8236744** | **1.608196** |
| aisabd | | **1.000276** | **.1177066** | **0.00** | **0.998** | | **.794247** | **1.25975** |
| aisext | | **1.407377** | **.2397332** | **2.01** | **0.045** | | **1.007898** | **1.96519** |
| gcsmcat2  1 | | **1.170332** | **.338502** | **0.54** | **0.587** | | **.6639131** | **2.063037** |
| 2 | | **1.998457** | **.4370924** | **3.17** | **0.002** | | **1.30174** | **3.068071** |
| 99 | | **1.538236** | **.6361867** | **1.04** | **0.298** | | **.6838824** | **3.459908** |
| pulse2 | |  | | | | | | |
| 1 | | **1.406506** | **.2554658** | **1.88** | **0.060** | | **.9852278** | **2.007921** |
| 2 | | **2.395103** | **.8349797** | **2.51** | **0.012** | | **1.209424** | **4.743181** |
| 99 | | **.3718883** | **.1555658** | **-2.36** | **0.018** | | **.1638112** | **.8442699** |
| bp2 1 | | **1.481224** | **.3013912** | **1.93** | **0.054** | | **.9940838** | **2.207082** |
| 2 | | **1.37261** | **.4420457** | **0.98** | **0.325** | | **.7301633** | **2.580326** |
| 99 | | **1.58292** | **.5987194** | **1.21** | **0.225** | | **.7542284** | **3.322119** |
| race | | **1.797047** | **.3224795** | **3.27** | **0.001** | | **1.264188** | **2.554508** |
| blunt_ind | | **.9392769** | **.2543457** | **-0.23** | **0.817** | | **.5524561** | **1.596943** |
| transfer | | **.8960857** | **.1620387** | **-0.61** | **0.544** | | **.6286765** | **1.277238** |
| congestive_heart_failure | | **1.474142** | **1.035706** | **0.55** | **0.581** | | **.3719635** | **5.842228** |
| pvd | | **1.281637** | **1.536359** | **0.21** | **0.836** | | **.1222897** | **13.43199** |
| hypertension | | **.7146348** | **.1637552** | **-1.47** | **0.143** | | **.456074** | **1.119781** |
| dialysis | | **.6659844** | **.7212774** | **-0.38** | **0.707** | | **.0797245** | **5.563347** |
| documented_history_of_cirrhosis | | **4.106315** | **2.48916** | **2.33** | **0.020** | | **1.251619** | **13.47201** |
| metastasis | | **1** | (omitted) |  |  | |  |  |
| active_chemotherapy | | **1** | (omitted) |  |  | |  |  |
| obesity | | **1.509877** | **.41821** | **1.49** | **0.137** | | **.8773505** | **2.598422** |
| ascites | | **1** | (omitted) |  |  | |  |  |
| drug_use | | **.727666** | **.1012329** | **-2.29** | **0.022** | | **.554004** | **.9557652** |
| smoker | | **.7956198** | **.1273993** | **-1.43** | **0.153** | | **.5813079** | **1.088942** |
| psych | | **.214136** | **.0698698** | **-4.72** | **0.000** | | **.1129675** | **.4059062** |
| ac_coag | | **1.633578** | **.4965499** | **1.61** | **0.106** | | **.9003362** | **2.963979** |
| routine_steroid_use | | **4.215569** | **2.875183** | **2.11** | **0.035** | | **1.107397** | **16.04756** |
| year_admit  2013 | | **.8717031** | **.2156804** | **-0.55** | **0.579** | | **.536736** | **1.415717** |
| 2014 | | **.730737** | **.1557136** | **-1.47** | **0.141** | | **.4812576** | **1.109544** |
| 2015 | | **.9300951** | **.1958664** | **-0.34** | **0.731** | | **.6155665** | **1.405335** |
| 2016 | | **.8865292** | **.2919031** | **-0.37** | **0.715** | | **.4649671** | **1.690301** |
| 2017 | | **1.082327** | **.3030984** | **0.28** | **0.778** | | **.6251518** | **1.873837** |
| 2018 | | **1.506443** | **.5034144** | **1.23** | **0.220** | | **.7825341** | **2.900029** |
| _cons | | **.0581565** | **.0209596** | **-7.89** | **0.000** | | **.0286963** | **.1178615** |

Note: **_cons** estimates baseline odds.

97 .

98 .

1. . **Table 5 Data
2. . **Report OR for Diabetes

101 .

102 . // logit acute_renal_failure diabetes i.agecat male i.isscat aishn aisfac aischs aisabd aisext i

- if dialysis==1, or vce(cluster traumactr)

103 .

1. . gen any_nvsup_days=(nvsup_days>0)
2. . gen any_icu_days=(icudays>0)

106 .

1. . logit any_nvsup_days i.diabetes i.agecat male i.isscat aishn aisfac aischs aisabd aisext i.gcsmc
   - e(cluster traumactr)

Iteration 0: log pseudolikelihood = **-41092.51**

Iteration 1: log pseudolikelihood = **-28316.214**

Iteration 2: log pseudolikelihood = **-25808.002**

Iteration 3: log pseudolikelihood = **-25307.068**

Iteration 4: log pseudolikelihood = **-25301.34**

Iteration 5: log pseudolikelihood = **-25301.338**

| Logistic regression  Log pseudolikelihood | = | Number of obs Wald chi2(33) Prob > chi2  **-25301.338** Pseudo R2 | | | | =  =  =  = | **106,141**  **.**  **. 0.3843** | | |
| --- | --- | --- | --- | --- | --- | --- | --- | --- | --- |
| (Std. Err. adjusted for **35** clusters in traumactr) | | | | | | | | | |
| any_nvsup_days | | | Odds Ratio | Robust Std. Err. | z | P>\|z\| | | [95% Conf. | Interval] |
| 1.diabetes | | | **1.127489** | **.0362383** | **3.73** | **0.000** | | **1.058654** | **1.200799** |
| agecat 26-45 | | | **1.029779** | **.0386911** | **0.78** | **0.435** | | **.9566709** | **1.108475** |
| 46-65 | | | **1.162194** | **.0382861** | **4.56** | **0.000** | | **1.089526** | **1.239709** |
| 66-75 | | | **1.183979** | **.060588** | **3.30** | **0.001** | | **1.07099** | **1.308889** |
| >75 | | | **.7881256** | **.0446012** | **-4.21** | **0.000** | | **.7053825** | **.8805746** |
| male | | | **1.43296** | **.0396803** | **12.99** | **0.000** | | **1.357261** | **1.512881** |
| isscat 16-24 | | | **2.553104** | **.098916** | **24.19** | **0.000** | | **2.366411** | **2.754527** |
| 25-35 | | | **6.083357** | **.3828148** | **28.69** | **0.000** | | **5.377479** | **6.881893** |
| >35 | | | **7.247472** | **1.031161** | **13.92** | **0.000** | | **5.483763** | **9.578433** |
| aishn | | | **1.705445** | **.0898862** | **10.13** | **0.000** | | **1.538065** | **1.89104** |
| aisfac | | | **4.769226** | **.8167769** | **9.12** | **0.000** | | **3.409347** | **6.671516** |
| aischs | | | **1.465183** | **.0881823** | **6.35** | **0.000** | | **1.302154** | **1.648624** |
| aisabd | | | **1.549896** | **.0825684** | **8.23** | **0.000** | | **1.396227** | **1.720478** |
| aisext | | | **1.45112** | **.0782745** | **6.90** | **0.000** | | **1.305536** | **1.612938** |
| gcsmcat2  1 | | | **13.91726** | **.848173** | **43.21** | **0.000** | | **12.35032** | **15.68301** |
| 2 | | | **28.91575** | **5.541536** | **17.56** | **0.000** | | **19.86125** | **42.09808** |
| 99 | | | **2.279214** | **.2827591** | **6.64** | **0.000** | | **1.787249** | **2.906599** |
| pulse2 | | |  | | | | | | |
| 1 | | | **2.658573** | **.1337479** | **19.44** | **0.000** | | **2.408941** | **2.934073** |
| 2 | | | **.9367847** | **.1521788** | **-0.40** | **0.688** | | **.6813399** | **1.288** |

| 99 | **.3321751** | **.1129411** | **-3.24** | **0.001** | **.1705904** | **.6468142** |
| --- | --- | --- | --- | --- | --- | --- |
| bp2 1 | **2.366717** | **.1436414** | **14.19** | **0.000** | **2.101285** | **2.665678** |
| 2 | **.8865816** | **.2390921** | **-0.45** | **0.655** | **.5225981** | **1.504075** |
| 99 | **.8382097** | **.1525947** | **-0.97** | **0.332** | **.5866686** | **1.197602** |
| race | **.9322112** | **.0600394** | **-1.09** | **0.276** | **.8216603** | **1.057636** |
| blunt_ind | **.4431537** | **.0328433** | **-10.98** | **0.000** | **.3832389** | **.5124355** |
| transfer | **1.170204** | **.1034591** | **1.78** | **0.075** | **.9840246** | **1.391609** |
| congestive_heart_failure | **1.408657** | **.1088617** | **4.43** | **0.000** | **1.210665** | **1.639028** |
| pvd | **1.156027** | **.1650635** | **1.02** | **0.310** | **.8738337** | **1.52935** |
| hypertension | **1.073811** | **.0317703** | **2.41** | **0.016** | **1.013314** | **1.137921** |
| dialysis | **1.654603** | **.184026** | **4.53** | **0.000** | **1.330524** | **2.057619** |
| documented_history_of_cirrhosis | **1.816372** | **.1700683** | **6.37** | **0.000** | **1.511841** | **2.182245** |
| metastasis | **1.157995** | **.2014577** | **0.84** | **0.399** | **.8234217** | **1.628513** |
| active_chemotherapy | **.5325956** | **.1022601** | **-3.28** | **0.001** | **.3655645** | **.7759453** |
| obesity | **1.291377** | **.0705365** | **4.68** | **0.000** | **1.160271** | **1.437298** |
| ascites | **2.098785** | **.7615229** | **2.04** | **0.041** | **1.030667** | **4.273836** |
| drug_use | **1.351508** | **.0688803** | **5.91** | **0.000** | **1.223029** | **1.493484** |
| smoker | **.9677304** | **.0334769** | **-0.95** | **0.343** | **.9042919** | **1.035619** |
| psych | **1.160393** | **.0516303** | **3.34** | **0.001** | **1.063486** | **1.26613** |
| ac_coag | **1.318899** | **.0595508** | **6.13** | **0.000** | **1.207197** | **1.440937** |
| routine_steroid_use | **1.104764** | **.1147658** | **0.96** | **0.338** | **.901248** | **1.354236** |
| year_admit  2013 | **.8701442** | **.0497585** | **-2.43** | **0.015** | **.7778861** | **.9733443** |
| 2014 | **.9285576** | **.0674543** | **-1.02** | **0.308** | **.8053302** | **1.070641** |
| 2015 | **.8894884** | **.0744661** | **-1.40** | **0.162** | **.7548828** | **1.048096** |
| 2016 | **.8263438** | **.0731773** | **-2.15** | **0.031** | **.6946759** | **.982968** |
| 2017 | **.8117938** | **.058676** | **-2.88** | **0.004** | **.7045655** | **.9353412** |
| 2018 | **.7490654** | **.0600381** | **-3.60** | **0.000** | **.6401701** | **.8764841** |
| _cons | **.0450696** | **.0047705** | **-29.28** | **0.000** | **.0366258** | **.05546** |

Note: **_cons** estimates baseline odds.

1. . margins diabetes

Predictive margins Number of obs = **106,141**

Model VCE : **Robust**

Expression : **Pr(any_nvsup_days), predict()**

|  | Margin | Delta-method Std. Err. | z | P>\|z\| | [95% Conf. | Interval] |
| --- | --- | --- | --- | --- | --- | --- |
| diabetes  0 | **.1293448** | **.0035644** | **36.29** | **0.000** | **.1223588** | **.1363309** |
| 1 | **.1374515** | **.0042345** | **32.46** | **0.000** | **.129152** | **.145751** |

109 .

1. . logit any_icu_days i.diabetes i.agecat male i.isscat aishn aisfac aischs aisabd aisext i.gcsmcat
   - cluster traumactr)

Iteration 0: log pseudolikelihood = **-70017.819**

Iteration 1: log pseudolikelihood = **-55597.922**

Iteration 2: log pseudolikelihood = **-55476.847**

Iteration 3: log pseudolikelihood = **-55476.619**

Iteration 4: log pseudolikelihood = **-55476.619**

| Logistic regression  Log pseudolikelihood | Number of obs Wald chi2(33) Prob > chi2  = **-55476.619** Pseudo R2 | | | | =  =  =  = | **106,141**  **.**  **. 0.2077** | | |
| --- | --- | --- | --- | --- | --- | --- | --- | --- |
| (Std. Err. adjusted for **35** clusters in traumactr) | | | | | | | | |
| any_icu_days | | Odds Ratio | Robust Std. Err. | z | P>\|z\| | | [95% Conf. | Interval] |
| 1.diabetes | | **1.038588** | **.032631** | **1.21** | **0.228** | | **.9765619** | **1.104554** |
| agecat 26-45 | | **.9336448** | **.0314777** | **-2.04** | **0.042** | | **.8739439** | **.997424** |
| 46-65 | | **1.029045** | **.0649082** | **0.45** | **0.650** | | **.9093765** | **1.164461** |
| 66-75 | | **1.199404** | **.096579** | **2.26** | **0.024** | | **1.024294** | **1.404451** |
| >75 | | **1.116585** | **.1253164** | **0.98** | **0.326** | | **.8961074** | **1.39131** |
| male | | **1.177714** | **.0239335** | **8.05** | **0.000** | | **1.131727** | **1.225569** |
| isscat 16-24 | | **2.534425** | **.186753** | **12.62** | **0.000** | | **2.193599** | **2.928205** |
| 25-35 | | **4.058072** | **.4297036** | **13.23** | **0.000** | | **3.297518** | **4.994043** |
| >35 | | **2.202316** | **.3869576** | **4.49** | **0.000** | | **1.5607** | **3.107706** |
| aishn | | **4.018758** | **.54486** | **10.26** | **0.000** | | **3.080964** | **5.242001** |
| aisfac | | **2.299584** | **.4403099** | **4.35** | **0.000** | | **1.580034** | **3.346817** |
| aischs | | **1.654873** | **.1240808** | **6.72** | **0.000** | | **1.428705** | **1.916845** |
| aisabd | | **2.334899** | **.2506106** | **7.90** | **0.000** | | **1.891936** | **2.881574** |
| aisext | | **.8994523** | **.1078139** | **-0.88** | **0.377** | | **.7111281** | **1.137649** |
| gcsmcat2  1 | | **4.1196** | **.3151654** | **18.51** | **0.000** | | **3.545968** | **4.786029** |
| 2 | | **1.980677** | **.2793529** | **4.85** | **0.000** | | **1.502315** | **2.611358** |
| 99 | | **.9894103** | **.1571123** | **-0.07** | **0.947** | | **.7247878** | **1.350647** |
| pulse2 | |  | | | | | | |
| 1 | | **1.826067** | **.1002307** | **10.97** | **0.000** | | **1.639816** | **2.033472** |
| 2 | | **.9221839** | **.0659443** | **-1.13** | **0.257** | | **.8015841** | **1.060928** |
| 99 | | **.7079665** | **.1074961** | **-2.27** | **0.023** | | **.5257366** | **.9533607** |
| bp2 1 | | **2.158814** | **.1360061** | **12.22** | **0.000** | | **1.908047** | **2.442537** |
| 2 | | **.7800486** | **.1375703** | **-1.41** | **0.159** | | **.5520809** | **1.10215** |
| 99 | | **.9113208** | **.1117133** | **-0.76** | **0.449** | | **.7166839** | **1.158817** |
| race | | **.9670664** | **.1706531** | **-0.19** | **0.849** | | **.6843043** | **1.366669** |
| blunt_ind | | **.748969** | **.0516686** | **-4.19** | **0.000** | | **.6542483** | **.8574033** |
| transfer | | **1.597546** | **.215387** | **3.47** | **0.001** | | **1.226567** | **2.080729** |
| congestive_heart_failure | | **1.41964** | **.0809317** | **6.15** | **0.000** | | **1.269558** | **1.587465** |
| pvd | | **1.056454** | **.1123895** | **0.52** | **0.606** | | **.8576232** | **1.301381** |
| hypertension | | **1.034742** | **.0526754** | **0.67** | **0.502** | | **.9364836** | **1.14331** |
| dialysis | | **1.42639** | **.106529** | **4.76** | **0.000** | | **1.23216** | **1.651238** |
| documented_history_of_cirrhosis | | **1.587159** | **.1818285** | **4.03** | **0.000** | | **1.267958** | **1.986717** |
| metastasis | | **1.22174** | **.1396133** | **1.75** | **0.080** | | **.9765814** | **1.528443** |
| active_chemotherapy | | **1.482631** | **.187589** | **3.11** | **0.002** | | **1.157006** | **1.899901** |
| obesity | | **1.011059** | **.1140105** | **0.10** | **0.922** | | **.8105728** | **1.261133** |
| ascites | | **2.191465** | **.7678885** | **2.24** | **0.025** | | **1.102742** | **4.355071** |
| drug_use | | **1.259602** | **.0764147** | **3.80** | **0.000** | | **1.118394** | **1.41864** |
| smoker | | **.9403791** | **.0350489** | **-1.65** | **0.099** | | **.8741335** | **1.011645** |
| psych | | **1.080454** | **.0508964** | **1.64** | **0.100** | | **.9851659** | **1.18496** |
| ac_coag | | **1.348234** | **.07885** | **5.11** | **0.000** | | **1.202219** | **1.511983** |
| routine_steroid_use | | **1.185041** | **.1018276** | **1.98** | **0.048** | | **1.001364** | **1.40241** |
| year_admit  2013 | | **.9191598** | **.0738484** | **-1.05** | **0.294** | | **.7852403** | **1.075919** |
| 2014 | | **.917979** | **.0865038** | **-0.91** | **0.364** | | **.7631705** | **1.10419** |

| 2015 | **.9231107** | **.1004804** | **-0.74** | **0.462** | **.7457627** | **1.142633** |
| --- | --- | --- | --- | --- | --- | --- |
| 2016 | **.8535231** | **.089976** | **-1.50** | **0.133** | **.6941991** | **1.049413** |
| 2017 | **.8239514** | **.0847151** | **-1.88** | **0.060** | **.6735732** | **1.007902** |
| 2018 | **.8144748** | **.1036666** | **-1.61** | **0.107** | **.6346532** | **1.045247** |
| _cons | **.2452891** | **.051051** | **-6.75** | **0.000** | **.1631253** | **.3688376** |

Note: **_cons** estimates baseline odds.

1. . margins diabetes

Predictive margins Number of obs = **106,141**

Model VCE : **Robust**

Expression : **Pr(any_icu_days), predict()**

|  | Margin | Delta-method Std. Err. | z | P>\|z\| | [95% Conf. | Interval] |
| --- | --- | --- | --- | --- | --- | --- |
| diabetes  0 | **.3704483** | **.024474** | **15.14** | **0.000** | **.3224801** | **.4184165** |
| 1 | **.3769949** | **.0217988** | **17.29** | **0.000** | **.3342701** | **.4197198** |

112 .

1. . regress nvsup_days i.diabetes i.agecat male i.isscat aishn aisfac aischs aisabd aisext i.gcsmcat
   - _days>0, vce(cluster traumactr)

| Linear | regression | Number of obs F(33, 34)  Prob > F R-squared | | | =  =  =  = | **13,841**  **.**  **. 0.0644** | | |
| --- | --- | --- | --- | --- | --- | --- | --- | --- |
|  |  | Root MSE | | | = | **7.9603** | | |
| (Std. Err. adjusted for **35** clusters in traumactr) | | | | | | | | |
| nvsup_days | | Coef. | Robust Std. Err. | t | P>\|t\| | | [95% Conf. | Interval] |
| 1.diabetes | | **.9515499** | **.2989334** | **3.18** | **0.003** | | **.3440441** | **1.559056** |
| agecat 26-45 | | **.5152685** | **.1553624** | **3.32** | **0.002** | | **.1995342** | **.8310028** |
| 46-65 | | **1.166363** | **.2522378** | **4.62** | **0.000** | | **.6537545** | **1.678972** |
| 66-75 | | **1.105154** | **.3749629** | **2.95** | **0.006** | | **.3431375** | **1.86717** |
| >75 | | **.0092865** | **.32764** | **0.03** | **0.978** | | **-.656558** | **.6751311** |
| male | | **.5357596** | **.1685933** | **3.18** | **0.003** | | **.1931368** | **.8783824** |
| isscat 16-24 | | **1.044171** | **.2097745** | **4.98** | **0.000** | | **.6178583** | **1.470484** |
| 25-35 | | **1.177644** | **.2777548** | **4.24** | **0.000** | | **.6131784** | **1.74211** |
| >35 | | **1.773328** | **.509142** | **3.48** | **0.001** | | **.7386269** | **2.808029** |
| aishn | | **2.016433** | **.297685** | **6.77** | **0.000** | | **1.411464** | **2.621402** |
| aisfac | | **.4462886** | **.4679922** | **0.95** | **0.347** | | **-.5047859** | **1.397363** |
| aischs | | **1.866118** | **.2739653** | **6.81** | **0.000** | | **1.309354** | **2.422883** |
| aisabd | | **.5842928** | **.2675046** | **2.18** | **0.036** | | **.040658** | **1.127928** |
| aisext | | **.8112418** | **.1994533** | **4.07** | **0.000** | | **.4059038** | **1.21658** |
| gcsmcat2  1 | | **.1202226** | **.2443829** | **0.49** | **0.626** | | **-.3764231** | **.6168683** |
| 2 | | **-.387845** | **.2805585** | **-1.38** | **0.176** | | **-.9580084** | **.1823184** |
| 99 | | **-.1949677** | **.3554188** | **-0.55** | **0.587** | | **-.9172656** | **.5273303** |
| pulse2 | |  | | | | | | |

| 1 | **.2800281** | **.1322089** | **2.12** | **0.042** | **.0113473** | **.5487088** |
| --- | --- | --- | --- | --- | --- | --- |
| 2 | **.1335269** | **.4338877** | **0.31** | **0.760** | **-.7482389** | **1.015293** |
| 99 | **-1.26464** | **.8134997** | **-1.55** | **0.129** | **-2.91787** | **.3885904** |
| bp2 1 | **.5251825** | **.2682534** | **1.96** | **0.059** | **-.019974** | **1.070339** |
| 2 | **-1.230561** | **.5312201** | **-2.32** | **0.027** | **-2.31013** | **-.150992** |
| 99 | **1.17144** | **.793861** | **1.48** | **0.149** | **-.4418794** | **2.78476** |
| race | **.9366816** | **.2511409** | **3.73** | **0.001** | **.4263019** | **1.447061** |
| blunt_ind | **1.434285** | **.2266339** | **6.33** | **0.000** | **.9737099** | **1.894861** |
| transfer | **.5244311** | **.3123333** | **1.68** | **0.102** | **-.1103066** | **1.159169** |
| congestive_heart_failure | **.2069351** | **.4474842** | **0.46** | **0.647** | **-.7024622** | **1.116332** |
| pvd | **.0584887** | **.6090383** | **0.10** | **0.924** | **-1.179226** | **1.296203** |
| hypertension | **.5627853** | **.2271874** | **2.48** | **0.018** | **.1010849** | **1.024486** |
| dialysis | **-.9296353** | **.4550076** | **-2.04** | **0.049** | **-1.854322** | **-.0049486** |
| documented_history_of_cirrhosis | **-.5606515** | **.3697223** | **-1.52** | **0.139** | **-1.312018** | **.1907146** |
| metastasis | **-.0862817** | **.8623241** | **-0.10** | **0.921** | **-1.838735** | **1.666172** |
| active_chemotherapy | **-.4698514** | **.886006** | **-0.53** | **0.599** | **-2.270432** | **1.33073** |
| obesity | **1.46506** | **.4554996** | **3.22** | **0.003** | **.539373** | **2.390746** |
| ascites | **-.4426666** | **1.39354** | **-0.32** | **0.753** | **-3.27468** | **2.389346** |
| drug_use | **-.1269676** | **.1610089** | **-0.79** | **0.436** | **-.454177** | **.2002418** |
| smoker | **.3490641** | **.1453737** | **2.40** | **0.022** | **.0536292** | **.6444989** |
| psych | **-.1613837** | **.2064694** | **-0.78** | **0.440** | **-.58098** | **.2582127** |
| ac_coag | **-.4180962** | **.1747053** | **-2.39** | **0.022** | **-.7731402** | **-.0630523** |
| routine_steroid_use | **.380613** | **.5366106** | **0.71** | **0.483** | **-.7099108** | **1.471137** |
| year_admit  2013 | **-.5929329** | **.4822518** | **-1.23** | **0.227** | **-1.572986** | **.3871207** |
| 2014 | **-.6908942** | **.420162** | **-1.64** | **0.109** | **-1.544766** | **.1629777** |
| 2015 | **-.6722199** | **.49842** | **-1.35** | **0.186** | **-1.685131** | **.3406913** |
| 2016 | **-.7346825** | **.396362** | **-1.85** | **0.072** | **-1.540187** | **.070822** |
| 2017 | **-.9509214** | **.4339163** | **-2.19** | **0.035** | **-1.832745** | **-.0690973** |
| 2018 | **-1.255744** | **.5055114** | **-2.48** | **0.018** | **-2.283067** | **-.2284213** |
| _cons | **1.564198** | **.5347568** | **2.93** | **0.006** | **.4774415** | **2.650954** |

1. . margins diabetes

Predictive margins Number of obs = **13,841**

Model VCE : **Robust**

Expression : **Linear prediction, predict()**

|  | Margin | Delta-method Std. Err. | t | P>\|t\| | [95% Conf. | Interval] |
| --- | --- | --- | --- | --- | --- | --- |
| diabetes  0 | **6.549407** | **.2715053** | **24.12** | **0.000** | **5.997642** | **7.101172** |
| 1 | **7.500957** | **.3973637** | **18.88** | **0.000** | **6.693417** | **8.308497** |

115 .

116 . regress icudays i.diabetes i.agecat male i.isscat aishn aisfac aischs aisabd aisext i.gcsmcat2 i

- 0, vce(cluster traumactr)

Linear regression Number of obs = **39,415**

F(33, 34) = **.**

Prob > F = **.**

R-squared = **0.1323**

Root MSE = **6.6218**

(Std. Err. adjusted for **35** clusters in traumactr)

icudays

Robust Coef. Std. Err.

t

P>|t|

[95% Conf. Interval]

1.diabetes

**.5365277 .1219613**

**4.40 0.000**

**.2886725**

**.7843828**

agecat

| 26-45 | **.4563915** | **.0903572** | **5.05** | **0.000** | **.2727636** | **.6400195** |
| --- | --- | --- | --- | --- | --- | --- |
| 46-65 | **.9807032** | **.1505001** | **6.52** | **0.000** | **.6748502** | **1.286556** |
| 66-75 | **.9061314** | **.1902965** | **4.76** | **0.000** | **.5194023** | **1.29286** |
| >75 | **.3045546** | **.1817163** | **1.68** | **0.103** | **-.0647373** | **.6738465** |
| male | **.5398223** | **.0714232** | **7.56** | **0.000** | **.3946728** | **.6849718** |
| isscat 16-24 | **1.178108** | **.1246335** | **9.45** | **0.000** | **.9248219** | **1.431393** |
| 25-35 | **2.617405** | **.1926422** | **13.59** | **0.000** | **2.225909** | **3.008902** |
| >35 | **4.090713** | **.4276868** | **9.56** | **0.000** | **3.221549** | **4.959877** |
| aishn | **.506559** | **.1264057** | **4.01** | **0.000** | **.2496717** | **.7634463** |
| aisfac | **1.556689** | **.3709177** | **4.20** | **0.000** | **.8028935** | **2.310484** |
| aischs | **1.484898** | **.169475** | **8.76** | **0.000** | **1.140483** | **1.829312** |
| aisabd | **.7579959** | **.2028699** | **3.74** | **0.001** | **.3457146** | **1.170277** |
| aisext | **1.54088** | **.215244** | **7.16** | **0.000** | **1.103451** | **1.978308** |
| gcsmcat2  1 | **2.8205** | **.267423** | **10.55** | **0.000** | **2.277031** | **3.363969** |
| 2 | **2.913629** | **.3204166** | **9.09** | **0.000** | **2.262464** | **3.564794** |
| 99 | **.5850143** | **.1815836** | **3.22** | **0.003** | **.2159921** | **.9540366** |
| pulse2 |  | | | | | |
| 1 | **1.087504** | **.1140772** | **9.53** | **0.000** | **.8556712** | **1.319337** |
| 2 | **.3332929** | **.3381917** | **0.99** | **0.331** | **-.3539953** | **1.020581** |
| 99 | **-1.244696** | **.6162027** | **-2.02** | **0.051** | **-2.49697** | **.0075789** |
| bp2 1 | **1.162062** | **.2084513** | **5.57** | **0.000** | **.7384381** | **1.585686** |
| 2 | **-.7508028** | **.418744** | **-1.79** | **0.082** | **-1.601793** | **.1001875** |
| 99 | **1.965757** | **.6402886** | **3.07** | **0.004** | **.6645337** | **3.26698** |
| race | **.4263216** | **.1980976** | **2.15** | **0.039** | **.0237388** | **.8289044** |
| blunt_ind | **-.0008611** | **.1523761** | **-0.01** | **0.996** | **-.3105267** | **.3088045** |
| transfer | **.2983372** | **.2088279** | **1.43** | **0.162** | **-.1260522** | **.7227266** |
| congestive_heart_failure | **.4662961** | **.2181166** | **2.14** | **0.040** | **.0230298** | **.9095624** |
| pvd | **.0999689** | **.2920823** | **0.34** | **0.734** | **-.4936137** | **.6935515** |
| hypertension | **.2993221** | **.1118387** | **2.68** | **0.011** | **.0720385** | **.5266057** |
| dialysis | **.3048485** | **.2379651** | **1.28** | **0.209** | **-.1787547** | **.7884518** |
| documented_history_of_cirrhosis | **.3329339** | **.361144** | **0.92** | **0.363** | **-.4009992** | **1.066867** |
| metastasis | **-.1536264** | **.3919783** | **-0.39** | **0.698** | **-.9502222** | **.6429694** |
| active_chemotherapy | **-.7187821** | **.2291226** | **-3.14** | **0.004** | **-1.184415** | **-.2531489** |
| obesity | **1.018434** | **.2280819** | **4.47** | **0.000** | **.5549157** | **1.481952** |
| ascites | **-1.493266** | **1.070588** | **-1.39** | **0.172** | **-3.668963** | **.6824302** |
| drug_use | **.021392** | **.10193** | **0.21** | **0.835** | **-.1857546** | **.2285386** |
| smoker | **.1486738** | **.0903713** | **1.65** | **0.109** | **-.0349827** | **.3323303** |
| psych | **.0230051** | **.1303003** | **0.18** | **0.861** | **-.241797** | **.2878072** |
| ac_coag | **-.059361** | **.0991847** | **-0.60** | **0.553** | **-.2609287** | **.1422066** |
| routine_steroid_use | **.1477751** | **.2251853** | **0.66** | **0.516** | **-.3098565** | **.6054067** |

|  | year_admit | |  | | | | | |
| --- | --- | --- | --- | --- | --- | --- | --- | --- |
|  |  | 2013 | **-.2065653** | **.2541584** | **-0.81** | **0.422** | **-.7230773** | **.3099468** |
|  |  | 2014 | **-.2349076** | **.1891446** | **-1.24** | **0.223** | **-.6192957** | **.1494806** |
|  |  | 2015 | **-.2542788** | **.1984868** | **-1.28** | **0.209** | **-.6576525** | **.1490949** |
|  |  | 2016 | **-.4486002** | **.2567558** | **-1.75** | **0.090** | **-.9703907** | **.0731903** |
|  |  | 2017 | **-.5350909** | **.2361159** | **-2.27** | **0.030** | **-1.014936** | **-.0552457** |
|  |  | 2018 | **-.6407841** | **.2698608** | **-2.37** | **0.023** | **-1.189207** | **-.0923609** |
|  |  | _cons | **1.593103** | **.2573705** | **6.19** | **0.000** | **1.070063** | **2.116143** |
| 117 | . margins diabetes |  |  |  |  |  |  |  |

Predictive margins Number of obs = **39,415**

Model VCE : **Robust**

Expression : **Linear prediction, predict()**

|  | Margin | Delta-method Std. Err. | t | P>\|t\| | [95% Conf. | Interval] |
| --- | --- | --- | --- | --- | --- | --- |
| diabetes  0 | **5.245321** | **.2099131** | **24.99** | **0.000** | **4.818727** | **5.671916** |
| 1 | **5.781849** | **.2748994** | **21.03** | **0.000** | **5.223186** | **6.340512** |

118 .

1. . regress hospdays i.diabetes i.agecat male i.isscat aishn aisfac aischs aisabd aisext i.gcsmcat2
   - er traumactr)

| Linear | regression | Number of obs F(33, 34)  Prob > F R-squared | | | =  =  =  = | **105,818**  **.**  **. 0.1532** | | | |
| --- | --- | --- | --- | --- | --- | --- | --- | --- | --- |
|  |  | Root MSE | | | = | **6.4235** | | | |
|  | |  | (Std. Err. | adjusted | | for | **35** | clusters in | traumactr) |
| hospdays | | Coef. | Robust Std. Err. | t | | P>\|t\| |  | [95% Conf. | Interval] |
| 1.diabetes | | **.3497445** | **.0525505** | **6.66** | | **0.000** |  | **.2429491** | **.45654** |
| agecat 26-45 | | **.460897** | **.0876118** | **5.26** | | **0.000** |  | **.2828484** | **.6389456** |
| 46-65 | | **1.128717** | **.1341574** | **8.41** | | **0.000** |  | **.8560762** | **1.401358** |
| 66-75 | | **1.148028** | **.1307754** | **8.78** | | **0.000** |  | **.8822604** | **1.413796** |
| >75 | | **.837949** | **.1344292** | **6.23** | | **0.000** |  | **.5647561** | **1.111142** |
| male | | **.3276083** | **.0594361** | **5.51** | | **0.000** |  | **.2068197** | **.4483969** |
| isscat 16-24 | | **2.233613** | **.1657443** | **13.48** | | **0.000** |  | **1.89678** | **2.570446** |
| 25-35 | | **3.884153** | **.2227158** | **17.44** | | **0.000** |  | **3.43154** | **4.336766** |
| >35 | | **4.816078** | **.5170971** | **9.31** | | **0.000** |  | **3.76521** | **5.866946** |
| aishn | | **.6579725** | **.1341389** | **4.91** | | **0.000** |  | **.3853695** | **.9305755** |
| aisfac | | **3.049795** | **.4425276** | **6.89** | | **0.000** |  | **2.15047** | **3.949119** |
| aischs | | **1.365576** | **.1388211** | **9.84** | | **0.000** |  | **1.083457** | **1.647694** |
| aisabd | | **2.228193** | **.3105314** | **7.18** | | **0.000** |  | **1.597117** | **2.859269** |
| aisext | | **2.625678** | **.1881106** | **13.96** | | **0.000** |  | **2.243392** | **3.007965** |
| gcsmcat2  1 | | **3.347383** | **.2735982** | **12.23** | | **0.000** |  | **2.791365** | **3.903402** |
| 2 | | **1.861777** | **.4007758** | **4.65** | | **0.000** |  | **1.047303** | **2.676251** |

# 99 .4267033 .1700749 2.51 0.017 .0810695 .7723372

pulse2

| 1 | **1.508527** | **.1528386** | **9.87** | **0.000** | **1.197921** | **1.819132** |
| --- | --- | --- | --- | --- | --- | --- |
| 2 | **.0735288** | **.2777495** | **0.26** | **0.793** | **-.490926** | **.6379836** |
| 99 | **-.6491293** | **.461231** | **-1.41** | **0.168** | **-1.586463** | **.2882048** |
| bp2 1 | **1.70643** | **.2739065** | **6.23** | **0.000** | **1.149785** | **2.263075** |
| 2 | **-1.07101** | **.4572815** | **-2.34** | **0.025** | **-2.000318** | **-.1417021** |
| 99 | **.5777825** | **.4278924** | **1.35** | **0.186** | **-.2917996** | **1.447365** |
| race | **.3450812** | **.1435572** | **2.40** | **0.022** | **.0533378** | **.6368245** |
| blunt_ind | **-.7461916** | **.1161264** | **-6.43** | **0.000** | **-.9821889** | **-.5101943** |
| transfer | **.595048** | **.2756409** | **2.16** | **0.038** | **.0348783** | **1.155218** |
| congestive_heart_failure | **.7345677** | **.1265898** | **5.80** | **0.000** | **.4773064** | **.9918291** |
| pvd | **.4110022** | **.1904997** | **2.16** | **0.038** | **.0238603** | **.7981441** |
| hypertension | **.3041049** | **.0680927** | **4.47** | **0.000** | **.165724** | **.4424859** |
| dialysis | **.9204058** | **.1918783** | **4.80** | **0.000** | **.5304621** | **1.310349** |
| documented_history_of_cirrhosis | **1.684873** | **.3769986** | **4.47** | **0.000** | **.9187199** | **2.451027** |
| metastasis | **.1876977** | **.324882** | **0.58** | **0.567** | **-.472542** | **.8479374** |
| active_chemotherapy | **-.0748698** | **.2454596** | **-0.31** | **0.762** | **-.5737037** | **.4239641** |
| obesity | **.5419949** | **.170754** | **3.17** | **0.003** | **.194981** | **.8890089** |
| ascites | **-.8135971** | **1.098448** | **-0.74** | **0.464** | **-3.045912** | **1.418718** |
| drug_use | **.3514** | **.1259086** | **2.79** | **0.009** | **.0955229** | **.6072771** |
| smoker | **.0728655** | **.0783353** | **0.93** | **0.359** | **-.086331** | **.232062** |
| psych | **.3725802** | **.0980522** | **3.80** | **0.001** | **.1733142** | **.5718462** |
| ac_coag | **.2194395** | **.0547424** | **4.01** | **0.000** | **.1081895** | **.3306894** |
| routine_steroid_use | **.266393** | **.1811095** | **1.47** | **0.151** | **-.1016658** | **.6344519** |
| year_admit  2013 | **-.2721113** | **.1460198** | **-1.86** | **0.071** | **-.5688592** | **.0246366** |
| 2014 | **-.2879923** | **.1433538** | **-2.01** | **0.053** | **-.5793222** | **.0033376** |
| 2015 | **-.4537587** | **.1629552** | **-2.78** | **0.009** | **-.7849236** | **-.1225938** |
| 2016 | **-.5079044** | **.1921212** | **-2.64** | **0.012** | **-.8983418** | **-.1174671** |
| 2017 | **-.739432** | **.1871199** | **-3.95** | **0.000** | **-1.119706** | **-.3591586** |
| 2018 | **-.859787** | **.1826206** | **-4.71** | **0.000** | **-1.230917** | **-.4886573** |
| _cons | **2.768023** | **.160445** | **17.25** | **0.000** | **2.44196** | **3.094086** |

1. . margins diabetes

Predictive margins Number of obs = **105,818**

Model VCE : **Robust**

Expression : **Linear prediction, predict()**

|  | Margin | Delta-method Std. Err. | t | P>\|t\| | [95% Conf. | Interval] |
| --- | --- | --- | --- | --- | --- | --- |
| diabetes  0 | **5.342942** | **.1723982** | **30.99** | **0.000** | **4.992586** | **5.693297** |
| 1 | **5.692686** | **.1825553** | **31.18** | **0.000** | **5.321689** | **6.063683** |

121 .

122 .

123 . **Table 6 Data: Odds ratio of complication given DM, age ≥65

124 .

1. . foreach X of varlist comp_any comp_sev_any dead comp_infection comp_ssi urinary_tract_infection
   - omp_vte{ 2.
2. . preserve
3. keep if age >=65
4. sum age 5.
5. . di in red "`X'"
6. di
7. logit `X' diabetes i.agecat male i.isscat aishn aisfac aischs aisabd aisext i.gcsmcat
   - cluster traumactr)
8. di
9. di
10. 10.
11. . restore

11. }

(65,021 observations deleted)

| Variable | Obs Mean | Std. Dev. | Min | Max |
| --- | --- | --- | --- | --- |
| age | **41,120 80.18646** | **8.783708** | **65** | **108.55** |
| **comp_any** |  |  |  |  |
| Iteration 0: | log pseudolikelihood = | **-11036.445** |  |  |
| Iteration 1: | log pseudolikelihood = | **-10533.054** |  |  |
| Iteration 2: | log pseudolikelihood = | **-10152.682** |  |  |
| Iteration 3: | log pseudolikelihood = | **-9983.4234** |  |  |
| Iteration 4: | log pseudolikelihood = | **-9968.6744** |  |  |
| Iteration 5: | log pseudolikelihood = | **-9968.6665** |  |  |
| Iteration 6: | log pseudolikelihood = | **-9968.6665** |  |  |

Logistic regression Number of obs = **41,120**

Wald chi2(33) = **.**

Prob > chi2 = **.**

Log pseudolikelihood = **-9968.6665** Pseudo R2 = **0.0968**

(Std. Err. adjusted for **35** clusters in traumactr)

| comp_any diabetes agecat | Robust  Odds Ratio Std. Err. z P>\|z\| [95% Conf. Interval]  **1.256918 .0573959 5.01 0.000 1.149311 1.374599** | | | | | |
| --- | --- | --- | --- | --- | --- | --- |
| 66-75 | **.8387626** | **.1302925** | **-1.13** | **0.258** | **.6186062** | **1.137271** |
| >75 | **.8244945** | **.1233491** | **-1.29** | **0.197** | **.6149547** | **1.105433** |
| male | **1.496766** | **.0806858** | **7.48** | **0.000** | **1.346693** | **1.663564** |
| isscat 16-24 | **1.976837** | **.1218231** | **11.06** | **0.000** | **1.751924** | **2.230624** |
| 25-35 | **2.806342** | **.2294114** | **12.62** | **0.000** | **2.390876** | **3.294004** |
| >35 | **3.806081** | **.7036493** | **7.23** | **0.000** | **2.649184** | **5.468195** |
| aishn | **1.228108** | **.0894556** | **2.82** | **0.005** | **1.064718** | **1.41657** |
| aisfac | **2.436504** | **.8928296** | **2.43** | **0.015** | **1.188102** | **4.996667** |
| aischs | **1.851864** | **.1326627** | **8.60** | **0.000** | **1.609279** | **2.131017** |
| aisabd | **1.833154** | **.1815382** | **6.12** | **0.000** | **1.509747** | **2.22584** |
| aisext | **2.010449** | **.1220025** | **11.51** | **0.000** | **1.785001** | **2.26437** |
| gcsmcat2  1 | **2.189461** | **.1697313** | **10.11** | **0.000** | **1.880833** | **2.548731** |

| 2 | **1.474013** | **.1429376** | **4.00** | **0.000** | **1.218875** | **1.782559** |
| --- | --- | --- | --- | --- | --- | --- |
| 99 | **.9905603** | **.0753156** | **-0.12** | **0.901** | **.8534168** | **1.149743** |
| pulse2 |  |  |  |  |  |  |
| 1 | **1.603205** | **.188418** | **4.02** | **0.000** | **1.273359** | **2.018494** |
| 2 | **1.150572** | **.1938991** | **0.83** | **0.405** | **.8269248** | **1.600891** |
| 99 | **.7331588** | **.1577426** | **-1.44** | **0.149** | **.4809035** | **1.117733** |
| bp2 1 | **2.234546** | **.2039134** | **8.81** | **0.000** | **1.868585** | **2.672179** |
| 2 | **1.828489** | **.487451** | **2.26** | **0.024** | **1.08436** | **3.083269** |
| 99 | **1.194321** | **.2547874** | **0.83** | **0.405** | **.7861981** | **1.814303** |
| race | **1.059843** | **.0794795** | **0.78** | **0.438** | **.9149729** | **1.22765** |
| blunt_ind | **.73831** | **.1100145** | **-2.04** | **0.042** | **.5513184** | **.9887238** |
| transfer | **1.126236** | **.1044977** | **1.28** | **0.200** | **.9389677** | **1.350853** |
| congestive_heart_failure | **1.443042** | **.1146298** | **4.62** | **0.000** | **1.234988** | **1.686146** |
| pvd | **1.157696** | **.1503765** | **1.13** | **0.260** | **.8974898** | **1.493343** |
| hypertension | **1.006704** | **.0479282** | **0.14** | **0.888** | **.9170164** | **1.105164** |
| dialysis | **1.464738** | **.1734883** | **3.22** | **0.001** | **1.161291** | **1.847477** |
| documented_history_of_cirrhosis | **1.651775** | **.3285286** | **2.52** | **0.012** | **1.11854** | **2.439214** |
| metastasis | **1.051487** | **.2317976** | **0.23** | **0.820** | **.682588** | **1.619754** |
| active_chemotherapy | **1.012866** | **.2509831** | **0.05** | **0.959** | **.623202** | **1.646173** |
| obesity | **1.18384** | **.0919701** | **2.17** | **0.030** | **1.016635** | **1.378546** |
| ascites | **1.103909** | **1.033149** | **0.11** | **0.916** | **.1763168** | **6.911511** |
| drug_use | **1.284461** | **.2055495** | **1.56** | **0.118** | **.9386525** | **1.757668** |
| smoker | **1.237668** | **.0836232** | **3.16** | **0.002** | **1.084158** | **1.412914** |
| psych | **1.103763** | **.0702577** | **1.55** | **0.121** | **.9743041** | **1.250424** |
| ac_coag | **1.187328** | **.0707218** | **2.88** | **0.004** | **1.056501** | **1.334356** |
| routine_steroid_use | **1.259054** | **.1485755** | **1.95** | **0.051** | **.9990741** | **1.586686** |
| year_admit  2013 | **.9282492** | **.0976193** | **-0.71** | **0.479** | **.7553496** | **1.140726** |
| 2014 | **.9048403** | **.0664752** | **-1.36** | **0.173** | **.783497** | **1.044976** |
| 2015 | **.8385187** | **.080795** | **-1.83** | **0.068** | **.6942178** | **1.012814** |
| 2016 | **.7432696** | **.0979051** | **-2.25** | **0.024** | **.5741485** | **.9622069** |
| 2017 | **.5692917** | **.065808** | **-4.87** | **0.000** | **.453878** | **.7140533** |
| 2018 | **.5287136** | **.066222** | **-5.09** | **0.000** | **.4136247** | **.6758253** |
| _cons | **.0538296** | **.0146201** | **-10.76** | **0.000** | **.0316108** | **.0916656** |

Note: **_cons** estimates baseline odds.

(65,021 observations deleted)

Variable

age

# comp_sev_any

Obs Mean Std. Dev. Min Max

# 41,120 80.18646 8.783708 65 108.55

Iteration 0: log pseudolikelihood = **-9708.0416**

Iteration 1: log pseudolikelihood = **-9455.923**

Iteration 2: log pseudolikelihood = **-9164.3881**

Iteration 3: log pseudolikelihood = **-8680.9601**

Iteration 4: log pseudolikelihood = **-8671.4679**

Iteration 5: log pseudolikelihood = **-8671.4491**

Iteration 6: log pseudolikelihood = **-8671.4491**

| Logistic regression |  | Number of obs | = | **41,120** |
| --- | --- | --- | --- | --- |
|  |  | Wald chi2(33) | = | **.** |
|  |  | Prob > chi2 | = | **.** |
| Log pseudolikelihood | = **-8671.4491** | Pseudo R2 | = | **0.1068** |

(Std. Err. adjusted for **35** clusters in traumactr)

| comp_sev_any | Odds Ratio | Robust Std. Err. | z | P>\|z\| | [95% Conf. | Interval] |
| --- | --- | --- | --- | --- | --- | --- |
| diabetes | **1.257459** | **.0629827** | **4.57** | **0.000** | **1.13988** | **1.387165** |
| agecat 66-75 | **.8791761** | **.1322791** | **-0.86** | **0.392** | **.6546449** | **1.180717** |
| >75 | **.8324337** | **.1238557** | **-1.23** | **0.218** | **.6218728** | **1.114289** |
| male | **1.767121** | **.0894529** | **11.25** | **0.000** | **1.600213** | **1.951438** |
| isscat 16-24 | **2.058002** | **.1347491** | **11.02** | **0.000** | **1.810142** | **2.3398** |
| 25-35 | **3.03334** | **.2577272** | **13.06** | **0.000** | **2.568023** | **3.582971** |
| >35 | **4.079842** | **.7565147** | **7.58** | **0.000** | **2.83666** | **5.867857** |
| aishn | **1.198956** | **.0895986** | **2.43** | **0.015** | **1.035601** | **1.388078** |
| aisfac | **2.653241** | **1.019291** | **2.54** | **0.011** | **1.249603** | **5.633538** |
| aischs | **1.803047** | **.1375546** | **7.73** | **0.000** | **1.552633** | **2.093849** |
| aisabd | **2.031921** | **.1979476** | **7.28** | **0.000** | **1.678741** | **2.459405** |
| aisext | **2.095952** | **.1245248** | **12.46** | **0.000** | **1.865562** | **2.354794** |
| gcsmcat2  1 | **2.188135** | **.1848652** | **9.27** | **0.000** | **1.854215** | **2.58219** |
| 2 | **1.713083** | **.1874645** | **4.92** | **0.000** | **1.38239** | **2.122884** |
| 99 | **1.087634** | **.0963902** | **0.95** | **0.343** | **.9142104** | **1.293956** |
| pulse2 |  | | | | | |
| 1 | **1.637966** | **.1976307** | **4.09** | **0.000** | **1.293011** | **2.07495** |
| 2 | **1.240188** | **.2145881** | **1.24** | **0.213** | **.8834974** | **1.740884** |
| 99 | **.6042076** | **.1612796** | **-1.89** | **0.059** | **.3580775** | **1.019519** |
| bp2 1 | **2.309858** | **.2022887** | **9.56** | **0.000** | **1.94554** | **2.742397** |
| 2 | **2.054251** | **.5830076** | **2.54** | **0.011** | **1.177817** | **3.582854** |
| 99 | **1.239339** | **.2991354** | **0.89** | **0.374** | **.7722153** | **1.989033** |
| race | **1.097306** | **.0806042** | **1.26** | **0.206** | **.9501701** | **1.267225** |
| blunt_ind | **.9739824** | **.1358936** | **-0.19** | **0.850** | **.7409491** | **1.280306** |
| transfer | **1.02758** | **.0957881** | **0.29** | **0.770** | **.8559908** | **1.233566** |
| congestive_heart_failure | **1.435641** | **.1145124** | **4.53** | **0.000** | **1.227865** | **1.678576** |
| pvd | **1.154984** | **.1520627** | **1.09** | **0.274** | **.892295** | **1.495007** |
| hypertension | **1.016377** | **.0531274** | **0.31** | **0.756** | **.9174058** | **1.126026** |
| dialysis | **1.556543** | **.1874923** | **3.67** | **0.000** | **1.229221** | **1.971024** |
| documented_history_of_cirrhosis | **1.45105** | **.3688556** | **1.46** | **0.143** | **.8816729** | **2.388125** |
| metastasis | **1.009124** | **.2313797** | **0.04** | **0.968** | **.6438347** | **1.581665** |
| active_chemotherapy | **1.201506** | **.2877104** | **0.77** | **0.443** | **.7514478** | **1.921114** |
| obesity | **1.329874** | **.1249044** | **3.04** | **0.002** | **1.106277** | **1.598663** |
| ascites | **.7863018** | **.9886412** | **-0.19** | **0.848** | **.0668879** | **9.243381** |
| drug_use | **1.248697** | **.2086238** | **1.33** | **0.184** | **.9000035** | **1.732487** |
| smoker | **1.316037** | **.084015** | **4.30** | **0.000** | **1.161256** | **1.491449** |
| psych | **1.08777** | **.0635929** | **1.44** | **0.150** | **.9700064** | **1.219832** |
| ac_coag | **1.143983** | **.0756116** | **2.04** | **0.042** | **1.004985** | **1.302206** |
| routine_steroid_use | **1.342967** | **.1714941** | **2.31** | **0.021** | **1.045608** | **1.724893** |
| year_admit  2013 | **.9821173** | **.1099703** | **-0.16** | **0.872** | **.7885914** | **1.223136** |
| 2014 | **.9353911** | **.0840763** | **-0.74** | **0.457** | **.7843037** | **1.115584** |
| 2015 | **.871442** | **.0776428** | **-1.54** | **0.122** | **.7318112** | **1.037715** |
| 2016 | **.8935241** | **.1161788** | **-0.87** | **0.387** | **.6925169** | **1.152875** |
| 2017 | **.7316347** | **.0856296** | **-2.67** | **0.008** | **.5816621** | **.9202755** |
| 2018 | **.6848757** | **.0852235** | **-3.04** | **0.002** | **.5366502** | **.8740418** |
| _cons | **.024942** | **.0069311** | **-13.28** | **0.000** | **.0144675** | **.0430002** |

Note: **_cons** estimates baseline odds.

(65,021 observations deleted)

| Variable | Obs Mean | Std. Dev. | Min | Max |
| --- | --- | --- | --- | --- |
| age | **41,120 80.18646** | **8.783708** | **65** | **108.55** |
| **dead** |  |  |  |  |
| Iteration 0: | log pseudolikelihood = | **-8909.0274** |  |  |
| Iteration 1: | log pseudolikelihood = | **-6887.6227** |  |  |
| Iteration 2: | log pseudolikelihood = | **-6460.6632** |  |  |
| Iteration 3: | log pseudolikelihood = | **-6300.206** |  |  |
| Iteration 4: | log pseudolikelihood = | **-6298.2653** |  |  |
| Iteration 5: | log pseudolikelihood = | **-6298.2642** |  |  |
| Iteration 6: | log pseudolikelihood = | **-6298.2642** |  |  |

Logistic regression Number of obs = **41,120**

Wald chi2(33) = **.**

Prob > chi2 = **.**

Log pseudolikelihood = **-6298.2642** Pseudo R2 = **0.2930**

(Std. Err. adjusted for **35** clusters in traumactr)

| dead diabetes agecat | Robust  Odds Ratio Std. Err. z P>\|z\| [95% Conf. Interval]  **1.082492 .073455 1.17 0.243 .9476862 1.236474** | | | | | |
| --- | --- | --- | --- | --- | --- | --- |
| 66-75 | **1.242579** | **.2672373** | **1.01** | **0.313** | **.81519** | **1.89404** |
| >75 | **2.625694** | **.5737381** | **4.42** | **0.000** | **1.710995** | **4.029391** |
| male | **1.530513** | **.1101768** | **5.91** | **0.000** | **1.329113** | **1.762432** |
| isscat 16-24 | **2.198329** | **.2288953** | **7.57** | **0.000** | **1.792518** | **2.696012** |
| 25-35 | **6.841613** | **.645386** | **20.39** | **0.000** | **5.68673** | **8.231035** |
| >35 | **14.64537** | **2.741227** | **14.34** | **0.000** | **10.14794** | **21.136** |
| aishn | **1.293143** | **.1054587** | **3.15** | **0.002** | **1.10212** | **1.517273** |
| aisfac | **.4181204** | **.2008085** | **-1.82** | **0.069** | **.163117** | **1.071775** |
| aischs | **1.255662** | **.1083138** | **2.64** | **0.008** | **1.060346** | **1.486954** |
| aisabd | **1.542842** | **.2110882** | **3.17** | **0.002** | **1.179946** | **2.017349** |
| aisext | **1.165046** | **.0777326** | **2.29** | **0.022** | **1.022234** | **1.32781** |
| gcsmcat2  1 | **4.838662** | **.4113026** | **18.55** | **0.000** | **4.096097** | **5.715843** |
| 2 | **18.34267** | **2.170984** | **24.58** | **0.000** | **14.5451** | **23.13174** |
| 99 | **1.210488** | **.1081209** | **2.14** | **0.032** | **1.016087** | **1.442082** |
| pulse2 |  | | | | | |
| 1 | **2.375256** | **.3261133** | **6.30** | **0.000** | **1.814863** | **3.108687** |
| 2 | **1.67121** | **.3460063** | **2.48** | **0.013** | **1.113778** | **2.507629** |
| 99 | **1.286379** | **.3082885** | **1.05** | **0.293** | **.8042174** | **2.057617** |
| bp2 1 | **3.033747** | **.4544175** | **7.41** | **0.000** | **2.261934** | **4.068916** |
| 2 | **2.891726** | **.7711149** | **3.98** | **0.000** | **1.714642** | **4.876864** |
| 99 | **1.27192** | **.3364613** | **0.91** | **0.363** | **.7573409** | **2.136134** |
| race | **.7885979** | **.0788541** | **-2.38** | **0.018** | **.6482487** | **.9593334** |
| blunt_ind | **.5797571** | **.1459974** | **-2.16** | **0.030** | **.3539094** | **.9497297** |
| transfer | **.7761243** | **.0645901** | **-3.05** | **0.002** | **.6593154** | **.913628** |
| congestive_heart_failure | **1.740977** | **.1513512** | **6.38** | **0.000** | **1.46823** | **2.064391** |

| pvd | **1.471143** | **.2629244** | **2.16** | **0.031** | **1.036399** | **2.08825** |
| --- | --- | --- | --- | --- | --- | --- |
| hypertension | **.8153668** | **.0466285** | **-3.57** | **0.000** | **.7289122** | **.9120756** |
| dialysis | **2.435098** | **.4135624** | **5.24** | **0.000** | **1.745636** | **3.396874** |
| documented_history_of_cirrhosis | **2.424628** | **.7046189** | **3.05** | **0.002** | **1.371761** | **4.285604** |
| metastasis | **2.044276** | **.4475784** | **3.27** | **0.001** | **1.330993** | **3.139811** |
| active_chemotherapy | **1.026635** | **.2891716** | **0.09** | **0.926** | **.5910973** | **1.783091** |
| obesity | **1.041111** | **.17319** | **0.24** | **0.809** | **.7514479** | **1.442432** |
| ascites | **2.912211** | **1.159937** | **2.68** | **0.007** | **1.3341** | **6.357076** |
| drug_use | **.5054782** | **.0949431** | **-3.63** | **0.000** | **.3498023** | **.730436** |
| smoker | **.8544617** | **.0922053** | **-1.46** | **0.145** | **.6915746** | **1.055714** |
| psych | **.9402461** | **.0732328** | **-0.79** | **0.429** | **.8071312** | **1.095315** |
| ac_coag | **1.269847** | **.0965522** | **3.14** | **0.002** | **1.094034** | **1.473914** |
| routine_steroid_use | **1.558815** | **.2438759** | **2.84** | **0.005** | **1.147162** | **2.118189** |
| year_admit  2013 | **.9638177** | **.154897** | **-0.23** | **0.819** | **.7033907** | **1.320666** |
| 2014 | **1.007264** | **.1301638** | **0.06** | **0.955** | **.7818915** | **1.297597** |
| 2015 | **.9932883** | **.1357205** | **-0.05** | **0.961** | **.7599223** | **1.298319** |
| 2016 | **1.051221** | **.1406421** | **0.37** | **0.709** | **.8087469** | **1.366393** |
| 2017 | **.9272118** | **.1099322** | **-0.64** | **0.524** | **.7349515** | **1.169767** |
| 2018 | **.7931195** | **.1039667** | **-1.77** | **0.077** | **.6134204** | **1.025461** |
| _cons | **.0126127** | **.0044754** | **-12.32** | **0.000** | **.0062917** | **.0252839** |
| Note: **_cons** estimates baseline odds. |  |  |  |  |  |  |

(65,021 observations deleted)

| Variable | Obs Mean | | Std. Dev. | Min | Max |  |
| --- | --- | --- | --- | --- | --- | --- |
| age | **41,120 80.18646** | | **8.783708** | **65** | **108.55** |  |
| **comp_infection** |  | |  |  |  |  |
| Iteration 0: | log pseudolikelihood = | | **-6877.8719** |  |  |  |
| Iteration 1: | log pseudolikelihood = | | **-6821.0836** |  |  |  |
| Iteration 2: | log pseudolikelihood = | | **-6210.0744** |  |  |  |
| Iteration 3: | log pseudolikelihood = | | **-6190.9954** |  |  |  |
| Iteration 4: | log pseudolikelihood = | | **-6190.4522** |  |  |  |
| Iteration 5: | log pseudolikelihood = | | **-6190.4517** |  |  |  |
| Logistic regression  Log pseudolikelihood | | = **-6190.4517** | Number of obs Wald chi2(33) Prob > chi2 Pseudo R2 | | =  =  =  = | **41,120**  **.**  **. 0.0999** |

|  | | (Std. Err. | adjusted | for | **35** | clusters in | traumactr) |
| --- | --- | --- | --- | --- | --- | --- | --- |
| comp_infection | Odds Ratio | Robust Std. Err. | z | P>\|z\| |  | [95% Conf. | Interval] |
| diabetes | **1.276063** | **.0815541** | **3.81** | **0.000** |  | **1.125825** | **1.446348** |
| agecat 66-75 | **.7837358** | **.1429604** | **-1.34** | **0.182** |  | **.5481545** | **1.120563** |
| >75 | **.7361811** | **.1365068** | **-1.65** | **0.099** |  | **.5118587** | **1.058813** |
| male | **1.354304** | **.1069825** | **3.84** | **0.000** |  | **1.160048** | **1.581089** |
| isscat 16-24 | **2.033127** | **.1391752** | **10.37** | **0.000** |  | **1.777855** | **2.32505** |
| 25-35 | **2.545229** | **.2593068** | **9.17** | **0.000** |  | **2.084523** | **3.107756** |
| >35 | **2.677834** | **.6431237** | **4.10** | **0.000** |  | **1.672455** | **4.287588** |
| aishn | **1.433454** | **.1198279** | **4.31** | **0.000** |  | **1.216826** | **1.688647** |
| aisfac | **2.356225** | **1.040437** | **1.94** | **0.052** |  | **.9916355** | **5.598625** |

| aischs | **2.141731** | **.1674324** | **9.74** | **0.000** | **1.837474** | **2.496368** |
| --- | --- | --- | --- | --- | --- | --- |
| aisabd | **1.706979** | **.2295622** | **3.98** | **0.000** | **1.311459** | **2.221783** |
| aisext | **1.536055** | **.1190748** | **5.54** | **0.000** | **1.319538** | **1.788101** |
| gcsmcat2  1 | **2.209474** | **.2027727** | **8.64** | **0.000** | **1.84574** | **2.644888** |
| 2 | **1.165169** | **.1779524** | **1.00** | **0.317** | **.8637495** | **1.571774** |
| 99 | **.9026158** | **.1214496** | **-0.76** | **0.446** | **.69338** | **1.174991** |
| pulse2 |  |  |  |  |  |  |
| 1 | **1.280586** | **.1841174** | **1.72** | **0.085** | **.9661102** | **1.697427** |
| 2 | **.5735599** | **.1749264** | **-1.82** | **0.068** | **.3154832** | **1.042753** |
| 99 | **.7762574** | **.1793686** | **-1.10** | **0.273** | **.4935373** | **1.220932** |
| bp2 1 | **1.952924** | **.2334803** | **5.60** | **0.000** | **1.544972** | **2.468596** |
| 2 | **1.098919** | **.4313176** | **0.24** | **0.810** | **.5091849** | **2.37168** |
| 99 | **1.139198** | **.2266467** | **0.66** | **0.512** | **.7713481** | **1.682474** |
| race | **1.068311** | **.1008897** | **0.70** | **0.484** | **.8877924** | **1.285535** |
| blunt_ind | **.5440337** | **.0999532** | **-3.31** | **0.001** | **.3795228** | **.7798547** |
| transfer | **1.184581** | **.1320993** | **1.52** | **0.129** | **.9520118** | **1.473964** |
| congestive_heart_failure | **1.432421** | **.1609327** | **3.20** | **0.001** | **1.149313** | **1.785268** |
| pvd | **1.093728** | **.1956473** | **0.50** | **0.616** | **.7702747** | **1.553007** |
| hypertension | **1.016064** | **.046715** | **0.35** | **0.729** | **.928508** | **1.111876** |
| dialysis | **.9529725** | **.1834705** | **-0.25** | **0.802** | **.6534363** | **1.389817** |
| documented_history_of_cirrhosis | **1.524988** | **.3339545** | **1.93** | **0.054** | **.9928038** | **2.342446** |
| metastasis | **1.023284** | **.2521882** | **0.09** | **0.926** | **.6312734** | **1.658726** |
| active_chemotherapy | **.7750575** | **.2789168** | **-0.71** | **0.479** | **.3828384** | **1.569106** |
| obesity | **1.04277** | **.1236115** | **0.35** | **0.724** | **.8265822** | **1.315502** |
| ascites | **2.591186** | **2.123709** | **1.16** | **0.245** | **.5198318** | **12.91619** |
| drug_use | **1.546053** | **.2714575** | **2.48** | **0.013** | **1.095896** | **2.181118** |
| smoker | **1.300342** | **.1282787** | **2.66** | **0.008** | **1.071733** | **1.577716** |
| psych | **1.264169** | **.1102258** | **2.69** | **0.007** | **1.065582** | **1.499766** |
| ac_coag | **1.232729** | **.0783063** | **3.29** | **0.001** | **1.088422** | **1.39617** |
| routine_steroid_use | **1.096136** | **.1776609** | **0.57** | **0.571** | **.7978149** | **1.506005** |
| year_admit  2013 | **.8301905** | **.1096197** | **-1.41** | **0.159** | **.6408904** | **1.075404** |
| 2014 | **.7940041** | **.0744712** | **-2.46** | **0.014** | **.6606734** | **.9542422** |
| 2015 | **.7207482** | **.1027837** | **-2.30** | **0.022** | **.5449998** | **.9531709** |
| 2016 | **.4945175** | **.0882432** | **-3.95** | **0.000** | **.3485706** | **.7015726** |
| 2017 | **.3743498** | **.0491135** | **-7.49** | **0.000** | **.2894694** | **.4841195** |
| 2018 | **.3297086** | **.0498672** | **-7.34** | **0.000** | **.2451258** | **.4434773** |
| _cons | **.0522649** | **.0152705** | **-10.10** | **0.000** | **.0294788** | **.0926636** |

Note: **_cons** estimates baseline odds.

(65,021 observations deleted)

Variable

age

Obs Mean Std. Dev. Min Max

# 41,120 80.18646 8.783708 65 108.55

**comp_ssi**

note: aisfac != 0 predicts failure perfectly aisfac dropped and 80 obs not used

note: 2.pulse2 != 0 predicts failure perfectly 2.pulse2 dropped and 443 obs not used

note: congestive_heart_failure != 0 predicts failure perfectly congestive_heart_failure dropped and 2833 obs not used

note: metastasis != 0 predicts failure perfectly metastasis dropped and 292 obs not used

note: active_chemotherapy != 0 predicts failure perfectly active_chemotherapy dropped and 180 obs not used

note: ascites != 0 predicts failure perfectly ascites dropped and 10 obs not used

note: routine_steroid_use != 0 predicts failure perfectly routine_steroid_use dropped and 880 obs not used

Iteration 0: log pseudolikelihood = **-386.06248**

Iteration 1: log pseudolikelihood = **-352.39958**

Iteration 2: log pseudolikelihood = **-337.38665**

Iteration 3: log pseudolikelihood = **-335.77745**

Iteration 4: log pseudolikelihood = **-335.77498**

Iteration 5: log pseudolikelihood = **-335.77498**

Logistic regression Number of obs = **36,402**

Wald chi2(33) = **.**

Prob > chi2 = **.**

Log pseudolikelihood = **-335.77498** Pseudo R2 = **0.1303**

(Std. Err. adjusted for **35** clusters in traumactr)

| comp_ssi diabetes agecat | Robust  Odds Ratio Std. Err. z P>\|z\| [95% Conf. Interval]  **1.670738 .5876721 1.46 0.145 .8385007 3.328997** | | | | | |
| --- | --- | --- | --- | --- | --- | --- |
| 66-75 | **.3676244** | **.1771862** | **-2.08** | **0.038** | **.1429372** | **.9455037** |
| >75 | **.2149841** | **.1337734** | **-2.47** | **0.013** | **.0634963** | **.7278881** |
| male | **1.267221** | **.447054** | **0.67** | **0.502** | **.6346918** | **2.530124** |
| isscat 16-24 | **2.952107** | **1.266897** | **2.52** | **0.012** | **1.273031** | **6.845816** |
| 25-35 | **4.226738** | **2.317941** | **2.63** | **0.009** | **1.442802** | **12.38237** |
| >35 | **2.799961** | **1.81725** | **1.59** | **0.113** | **.784692** | **9.990902** |
| aishn | **.7714445** | **.4176824** | **-0.48** | **0.632** | **.2669559** | **2.229307** |
| aisfac | **1** | (omitted) |  |  |  |  |
| aischs | **2.082654** | **.6107051** | **2.50** | **0.012** | **1.172238** | **3.700142** |
| aisabd | **1.554856** | **.7644104** | **0.90** | **0.369** | **.5932203** | **4.075346** |
| aisext | **1.498964** | **.545609** | **1.11** | **0.266** | **.7344496** | **3.05929** |
| gcsmcat2  1 | **.7557868** | **.4123053** | **-0.51** | **0.608** | **.2594432** | **2.201691** |
| 2 | **.2677663** | **.199803** | **-1.77** | **0.077** | **.0620301** | **1.155871** |
| 99 | **.8883684** | **.453087** | **-0.23** | **0.816** | **.3269354** | **2.413928** |
| pulse2 |  | | | | | |
| 1 | **1.867558** | **.7982051** | **1.46** | **0.144** | **.8081011** | **4.316011** |
| 2 | **1** | (empty) |  |  |  |  |

99

| **.6791085** | **.5488877** | **-0.48** | **0.632** | **.1393021** | **3.310706** |
| --- | --- | --- | --- | --- | --- |
| **2.527311** | **1.097096** | **2.14** | **0.033** | **1.079333** | **5.917823** |
| **7.130378** | **5.556495** | **2.52** | **0.012** | **1.54809** | **32.84196** |
| **2.550072** | **1.703358** | **1.40** | **0.161** | **.6886201** | **9.443329** |
| **1.020432** | **.4489219** | **0.05** | **0.963** | **.4308366** | **2.416885** |
| **.1093815** | **.0688725** | **-3.51** | **0.000** | **.0318406** | **.3757566** |
| **1.617629** | **.6673389** | **1.17** | **0.244** | **.720648** | **3.631072** |
| **1** | (omitted) |  |  |  |  |

bp2 1

2

99

race blunt_ind transfer

congestive_heart_failure

| pvd | **1.653728** | **1.838525** | **0.45** | **0.651** | **.1871306** | **14.61448** |
| --- | --- | --- | --- | --- | --- | --- |
| hypertension | **.469206** | **.140916** | **-2.52** | **0.012** | **.2604494** | **.8452862** |
| dialysis | **2.506801** | **1.853036** | **1.24** | **0.214** | **.5887202** | **10.67409** |
| documented_history_of_cirrhosis | **1.868796** | **1.58025** | **0.74** | **0.460** | **.3562771** | **9.802481** |
| metastasis | **1** | (omitted) |  |  |  |  |
| active_chemotherapy | **1** | (omitted) |  |  |  |  |
| obesity | **1.740649** | **.9092702** | **1.06** | **0.289** | **.6252667** | **4.845705** |
| ascites | **1** | (omitted) |  |  |  |  |
| drug_use | **1.868866** | **1.004436** | **1.16** | **0.245** | **.651769** | **5.358742** |
| smoker | **.6903931** | **.2671028** | **-0.96** | **0.338** | **.3234292** | **1.473715** |
| psych | **1.322717** | **.4401838** | **0.84** | **0.401** | **.6889653** | **2.53943** |
| ac_coag | **1.1513** | **.3800461** | **0.43** | **0.670** | **.6028449** | **2.198728** |
| routine_steroid_use | **1** | (omitted) |  |  |  |  |
| year_admit  2013 | **1.810994** | **.8540205** | **1.26** | **0.208** | **.7186396** | **4.56376** |
| 2014 | **1.683084** | **.8981009** | **0.98** | **0.329** | **.591427** | **4.789725** |
| 2015 | **1.3597** | **.6004636** | **0.70** | **0.487** | **.5721898** | **3.231069** |
| 2016 | **1.305732** | **.7618787** | **0.46** | **0.648** | **.4160911** | **4.097508** |
| 2017 | **1.139863** | **.6888954** | **0.22** | **0.829** | **.3486703** | **3.72641** |
| 2018 | **1.032181** | **.4393501** | **0.07** | **0.941** | **.448167** | **2.377233** |
| _cons | **.0135021** | **.0119051** | **-4.88** | **0.000** | **.0023982** | **.0760189** |
| Note: **_cons** estimates baseline odds. |  |  |  |  |  |  |

(65,021 observations deleted)

Variable

age

Obs Mean Std. Dev. Min Max

# 41,120 80.18646 8.783708 65 108.55

**urinary_tract_infection**

Iteration 0: log pseudolikelihood = **-3072.8495**

Iteration 1: log pseudolikelihood = **-2960.0688**

Iteration 2: log pseudolikelihood = **-2777.3355**

Iteration 3: log pseudolikelihood = **-2776.1753**

Iteration 4: log pseudolikelihood = **-2776.1735**

Iteration 5: log pseudolikelihood = **-2776.1735**

| Logistic regression  Log pseudolikelihood | Number of obs Wald chi2(33) Prob > chi2  = **-2776.1735** Pseudo R2 | | | | =  =  =  = | **41,120**  **.**  **. 0.0965** | | | |
| --- | --- | --- | --- | --- | --- | --- | --- | --- | --- |
|  | |  | (Std. Err. | adjusted | | for | **35** | clusters in | traumactr) |
| urinary_tract_infection | | Odds Ratio | Robust Std. Err. | z | | P>\|z\| |  | [95% Conf. | Interval] |
| diabetes | | **1.150572** | **.0883151** | **1.83** | | **0.068** |  | **.9898688** | **1.337365** |
| agecat 66-75 | | **1.163517** | **.5154182** | **0.34** | | **0.732** |  | **.4883205** | **2.772302** |
| >75 | | **1.366069** | **.5786958** | **0.74** | | **0.462** |  | **.5955053** | **3.133716** |
| male | | **.6142002** | **.0670575** | **-4.46** | | **0.000** |  | **.4958804** | **.7607517** |
| isscat 16-24 | | **1.633319** | **.1911647** | **4.19** | | **0.000** |  | **1.298511** | **2.054452** |
| 25-35 | | **1.830986** | **.3197416** | **3.46** | | **0.001** |  | **1.300293** | **2.578272** |
| >35 | | **1.538264** | **.4767884** | **1.39** | | **0.165** |  | **.8379134** | **2.823988** |
| aishn | | **1.511022** | **.1464858** | **4.26** | | **0.000** |  | **1.249543** | **1.827219** |
| aisfac | | **.6624377** | **.7101337** | **-0.38** | | **0.701** |  | **.081032** | **5.415439** |

| aischs | **1.986051** | **.2296736** | **5.93** | **0.000** | **1.583269** | **2.491302** |
| --- | --- | --- | --- | --- | --- | --- |
| aisabd | **1.441754** | **.4348597** | **1.21** | **0.225** | **.7982763** | **2.60393** |
| aisext | **1.537308** | **.178439** | **3.70** | **0.000** | **1.224503** | **1.93002** |
| gcsmcat2  1 | **1.84911** | **.2666751** | **4.26** | **0.000** | **1.393811** | **2.453134** |
| 2 | **.8258322** | **.1747416** | **-0.90** | **0.366** | **.5454843** | **1.250263** |
| 99 | **.7354219** | **.1001494** | **-2.26** | **0.024** | **.5631451** | **.9604015** |
| pulse2 |  |  |  |  |  |  |
| 1 | **.9078379** | **.1752707** | **-0.50** | **0.617** | **.6218305** | **1.325393** |
| 2 | **.6914182** | **.3027675** | **-0.84** | **0.399** | **.2930928** | **1.631084** |
| 99 | **.8163942** | **.2729571** | **-0.61** | **0.544** | **.4239407** | **1.572153** |
| bp2 1 | **1.484859** | **.3643071** | **1.61** | **0.107** | **.9180043** | **2.401739** |
| 2 | **1.208072** | **.6535563** | **0.35** | **0.727** | **.4184081** | **3.488071** |
| 99 | **1.528711** | **.4808603** | **1.35** | **0.177** | **.8252346** | **2.831869** |
| race | **1.133959** | **.2104869** | **0.68** | **0.498** | **.7881268** | **1.631543** |
| blunt_ind | **.4579938** | **.1634749** | **-2.19** | **0.029** | **.2275277** | **.9219024** |
| transfer | **1.38205** | **.1733999** | **2.58** | **0.010** | **1.080755** | **1.767341** |
| congestive_heart_failure | **1.477661** | **.2348217** | **2.46** | **0.014** | **1.082198** | **2.017637** |
| pvd | **1.262052** | **.4126216** | **0.71** | **0.477** | **.6649384** | **2.395374** |
| hypertension | **.8912598** | **.0805414** | **-1.27** | **0.203** | **.7465912** | **1.063961** |
| dialysis | **1.050254** | **.3211094** | **0.16** | **0.873** | **.5768248** | **1.912249** |
| documented_history_of_cirrhosis | **1.430025** | **.6580503** | **0.78** | **0.437** | **.5802949** | **3.524021** |
| metastasis | **1.272509** | **.5775136** | **0.53** | **0.595** | **.5228205** | **3.097198** |
| active_chemotherapy | **.5940496** | **.3905196** | **-0.79** | **0.428** | **.1637779** | **2.154717** |
| obesity | **1.0402** | **.2146018** | **0.19** | **0.848** | **.6942354** | **1.558571** |
| ascites | **1.878442** | **1.746623** | **0.68** | **0.498** | **.3036191** | **11.62162** |
| drug_use | **1.36915** | **.4917371** | **0.87** | **0.382** | **.6772323** | **2.767989** |
| smoker | **1.302276** | **.1573135** | **2.19** | **0.029** | **1.02773** | **1.650165** |
| psych | **1.074462** | **.2230876** | **0.35** | **0.729** | **.7152506** | **1.614075** |
| ac_coag | **1.404677** | **.1514051** | **3.15** | **0.002** | **1.137179** | **1.7351** |
| routine_steroid_use | **1.054396** | **.3023203** | **0.18** | **0.853** | **.6010968** | **1.849539** |
| year_admit  2013 | **.775551** | **.1628259** | **-1.21** | **0.226** | **.5139274** | **1.170359** |
| 2014 | **.6801546** | **.1132346** | **-2.32** | **0.021** | **.490791** | **.9425811** |
| 2015 | **.6371372** | **.1429132** | **-2.01** | **0.044** | **.4104914** | **.9889216** |
| 2016 | **.2898229** | **.0883002** | **-4.07** | **0.000** | **.1595135** | **.5265844** |
| 2017 | **.1255138** | **.0289359** | **-9.00** | **0.000** | **.0798833** | **.1972092** |
| 2018 | **.1080882** | **.0305834** | **-7.86** | **0.000** | **.0620772** | **.188202** |
| _cons | **.0312248** | **.0164013** | **-6.60** | **0.000** | **.0111531** | **.0874188** |

Note: **_cons** estimates baseline odds.

(65,021 observations deleted)

Variable

age

Obs Mean Std. Dev. Min Max

# 41,120 80.18646 8.783708 65 108.55

**cdiff**

note: 2.bp2 != 0 predicts failure perfectly 2.bp2 dropped and 129 obs not used

note: active_chemotherapy != 0 predicts failure perfectly active_chemotherapy dropped and 265 obs not used

Iteration 0: log pseudolikelihood = **-939.64219**

Iteration 1: log pseudolikelihood = **-938.89213**

Iteration 2: log pseudolikelihood = **-900.13474**

Iteration 3: log pseudolikelihood = **-894.36339** (backed up) Iteration 4: log pseudolikelihood = **-890.62854**

Iteration 5: log pseudolikelihood = **-890.43597**

Iteration 6: log pseudolikelihood = **-890.43527**

Iteration 7: log pseudolikelihood = **-890.43527**

Logistic regression Number of obs = **40,726**

Wald chi2(33) = **.**

Prob > chi2 = **.**

Log pseudolikelihood = **-890.43527** Pseudo R2 = **0.0524**

(Std. Err. adjusted for **35** clusters in traumactr)

| cdiff diabetes agecat | Robust  Odds Ratio Std. Err. z P>\|z\| [95% Conf. Interval]  **1.150996 .2572455 0.63 0.529 .7427311 1.783675** | | | | | |
| --- | --- | --- | --- | --- | --- | --- |
| 66-75 | **1.137095** | **.9174291** | **0.16** | **0.873** | **.233901** | **5.527919** |
| >75 | **1.124285** | **.8815396** | **0.15** | **0.881** | **.2418011** | **5.227503** |
| male | **1.606015** | **.3807113** | **2.00** | **0.046** | **1.009182** | **2.555817** |
| isscat 16-24 | **2.272916** | **.5306316** | **3.52** | **0.000** | **1.438346** | **3.59173** |
| 25-35 | **2.382205** | **.8054997** | **2.57** | **0.010** | **1.227893** | **4.621656** |
| >35 | **2.874653** | **1.551496** | **1.96** | **0.050** | **.99811** | **8.279276** |
| aishn | **.8145419** | **.1430474** | **-1.17** | **0.243** | **.5773352** | **1.149209** |
| aisfac | **2.47506** | **2.385474** | **0.94** | **0.347** | **.3742776** | **16.36732** |
| aischs | **1.269561** | **.2168313** | **1.40** | **0.162** | **.9083955** | **1.774321** |
| aisabd | **2.342768** | **.8671372** | **2.30** | **0.021** | **1.134151** | **4.839356** |
| aisext | **1.224975** | **.2348008** | **1.06** | **0.290** | **.8413385** | **1.783545** |
| gcsmcat2  1 | **1.800536** | **.6522132** | **1.62** | **0.104** | **.8852558** | **3.66214** |
| 2 | **.4350256** | **.2688724** | **-1.35** | **0.178** | **.1295449** | **1.460862** |
| 99 | **.920335** | **.2065626** | **-0.37** | **0.711** | **.5927886** | **1.428868** |
| pulse2 |  | | | | | |
| 1 | **1.417244** | **.4262267** | **1.16** | **0.246** | **.7860521** | **2.555276** |
| 2 | **.5348446** | **.5386588** | **-0.62** | **0.534** | **.0742943** | **3.850344** |
| 99 | **1.417311** | **.3188032** | **1.55** | **0.121** | **.912011** | **2.202574** |
| bp2 1 | **1.434293** | **.5691316** | **0.91** | **0.363** | **.6589902** | **3.121743** |
| 2 | **1** | (empty) |  |  |  |  |
| 99 | **.8247646** | **.2128** | **-0.75** | **0.455** | **.4974037** | **1.367575** |
| race | **.7576432** | **.2271377** | **-0.93** | **0.355** | **.4209967** | **1.363486** |
| blunt_ind | **.4146763** | **.1959407** | **-1.86** | **0.062** | **.1642494** | **1.046923** |
| transfer | **1.354361** | **.2754253** | **1.49** | **0.136** | **.9091434** | **2.017606** |
| congestive_heart_failure | **.7818732** | **.2871609** | **-0.67** | **0.503** | **.3806387** | **1.606052** |
| pvd | **.7978127** | **.5676489** | **-0.32** | **0.751** | **.1978179** | **3.217632** |
| hypertension | **1.280415** | **.2224057** | **1.42** | **0.155** | **.9109587** | **1.799712** |
| dialysis | **1.791215** | **.8068666** | **1.29** | **0.196** | **.7408281** | **4.330898** |
| documented_history_of_cirrhosis | **2.351159** | **1.476819** | **1.36** | **0.173** | **.6864711** | **8.052708** |
| metastasis | **1.025868** | **1.082396** | **0.02** | **0.981** | **.1297139** | **8.113277** |
| active_chemotherapy | **1** | (omitted) |  |  |  |  |
| obesity | **.9770043** | **.3855478** | **-0.06** | **0.953** | **.4508097** | **2.117384** |
| ascites | **7.285123** | **6.440257** | **2.25** | **0.025** | **1.288102** | **41.20249** |
| drug_use | **.729527** | **.4387575** | **-0.52** | **0.600** | **.2244427** | **2.37125** |
| smoker | **1.018903** | **.3158865** | **0.06** | **0.952** | **.5549303** | **1.870801** |

| psych | **1.63657** | **.3820652** | **2.11** | **0.035** | **1.035661** | **2.586138** |
| --- | --- | --- | --- | --- | --- | --- |
| ac_coag | **.8748426** | **.1458202** | **-0.80** | **0.422** | **.6310304** | **1.212857** |
| routine_steroid_use | **1.210547** | **.6262562** | **0.37** | **0.712** | **.4391638** | **3.336848** |
| year_admit  2013 | **2.473794** | **.7789534** | **2.88** | **0.004** | **1.334551** | **4.585552** |
| 2014 | **1.790749** | **.6445764** | **1.62** | **0.106** | **.8843956** | **3.62596** |
| 2015 | **1.197558** | **.4625635** | **0.47** | **0.641** | **.5617139** | **2.553159** |
| 2016 | **2.142916** | **.7664881** | **2.13** | **0.033** | **1.063025** | **4.319832** |
| 2017 | **.9070326** | **.3947454** | **-0.22** | **0.823** | **.3865231** | **2.128484** |
| 2018 | **.9796459** | **.3712512** | **-0.05** | **0.957** | **.4661158** | **2.058944** |
| _cons | **.0020332** | **.001703** | **-7.40** | **0.000** | **.0003938** | **.0104985** |

Note: **_cons** estimates baseline odds.

(65,021 observations deleted)

| Variable | Obs Mean | Std. Dev. | Min | Max |
| --- | --- | --- | --- | --- |
| age  **systemic_sepsis** | **41,120 80.18646** | **8.783708** | **65** | **108.55** |
| note: metastasis | != 0 predicts failure | perfectly |  |  |

metastasis dropped and 325 obs not used note: ascites != 0 predicts failure perfectly

ascites dropped and 15 obs not used

Iteration 0: log pseudolikelihood = **-1155.7404**

Iteration 1: log pseudolikelihood = **-1067.4651**

Iteration 2: log pseudolikelihood = **-1029.3317**

Iteration 3: log pseudolikelihood = **-1028.8167**

Iteration 4: log pseudolikelihood = **-1028.8147**

Iteration 5: log pseudolikelihood = **-1028.8147**

| Logistic regression  Log pseudolikelihood | Number of obs Wald chi2(33) Prob > chi2  = **-1028.8147** Pseudo R2 | | | | =  =  =  = | **40,780**  **.**  **. 0.1098** | | | |
| --- | --- | --- | --- | --- | --- | --- | --- | --- | --- |
|  | |  | (Std. Err. | adjusted | | for | **35** | clusters in | traumactr) |
| systemic_sepsis | | Odds Ratio | Robust Std. Err. | z | | P>\|z\| |  | [95% Conf. | Interval] |
| diabetes | | **1.686292** | **.2740191** | **3.22** | | **0.001** |  | **1.226349** | **2.318736** |
| agecat 66-75 | | **1.315013** | **.6879636** | **0.52** | | **0.601** |  | **.4716443** | **3.666448** |
| >75 | | **1.171668** | **.652253** | **0.28** | | **0.776** |  | **.3935058** | **3.488654** |
| male | | **1.779015** | **.2531329** | **4.05** | | **0.000** |  | **1.346057** | **2.351232** |
| isscat 16-24 | | **2.380602** | **.4656528** | **4.43** | | **0.000** |  | **1.622517** | **3.492885** |
| 25-35 | | **2.305926** | **.9025753** | **2.13** | | **0.033** |  | **1.07071** | **4.966139** |
| >35 | | **.6991781** | **.4695005** | **-0.53** | | **0.594** |  | **.1875008** | **2.607189** |
| aishn | | **1.589158** | **.3828599** | **1.92** | | **0.055** |  | **.9910498** | **2.548229** |
| aisfac | | **6.257237** | **3.41755** | **3.36** | | **0.001** |  | **2.145246** | **18.25106** |
| aischs | | **2.850417** | **.5008325** | **5.96** | | **0.000** |  | **2.019985** | **4.022247** |
| aisabd | | **2.955411** | **.8036028** | **3.99** | | **0.000** |  | **1.734477** | **5.035785** |
| aisext | | **2.213279** | **.4070753** | **4.32** | | **0.000** |  | **1.543404** | **3.173896** |

| gcsmcat2 |  | | | | | |
| --- | --- | --- | --- | --- | --- | --- |
| 1 | **1.505941** | **.4512358** | **1.37** | **0.172** | **.8370594** | **2.709315** |
| 2 | **1.634408** | **.7607795** | **1.06** | **0.291** | **.6563657** | **4.069821** |
| 99 | **.9287013** | **.2862577** | **-0.24** | **0.810** | **.5075823** | **1.699204** |
| pulse2 |  |  |  |  |  |  |
| 1 | **1.463541** | **.6228566** | **0.89** | **0.371** | **.6355486** | **3.370241** |
| 2 | **.3195581** | **.3350012** | **-1.09** | **0.276** | **.0409461** | **2.493945** |
| 99 | **1.045369** | **.5090837** | **0.09** | **0.927** | **.4024792** | **2.715163** |
| bp2 1 | **1.866016** | **.6442366** | **1.81** | **0.071** | **.948507** | **3.67105** |
| 2 | **1.531187** | **1.349703** | **0.48** | **0.629** | **.2720914** | **8.616708** |
| 99 | **1.177684** | **.5632832** | **0.34** | **0.732** | **.4612125** | **3.007158** |
| race | **1.489314** | **.3306365** | **1.79** | **0.073** | **.9638616** | **2.30122** |
| blunt_ind | **.4322582** | **.1714204** | **-2.11** | **0.034** | **.1986931** | **.9403809** |
| transfer | **1.048892** | **.1531415** | **0.33** | **0.744** | **.7878669** | **1.396397** |
| congestive_heart_failure | **1.991684** | **.3856663** | **3.56** | **0.000** | **1.362685** | **2.911023** |
| pvd | **.2205262** | **.231918** | **-1.44** | **0.151** | **.0280729** | **1.732338** |
| hypertension | **1.132209** | **.191604** | **0.73** | **0.463** | **.8126015** | **1.577523** |
| dialysis | **1.638008** | **.6511836** | **1.24** | **0.214** | **.7514921** | **3.570326** |
| documented_history_of_cirrhosis | **1.326166** | **.9572868** | **0.39** | **0.696** | **.322227** | **5.458003** |
| metastasis | **1** | (omitted) |  |  |  |  |
| active_chemotherapy | **2.989427** | **1.353966** | **2.42** | **0.016** | **1.230448** | **7.262944** |
| obesity | **1.270815** | **.3336446** | **0.91** | **0.361** | **.7596344** | **2.125984** |
| ascites | **1** | (omitted) |  |  |  |  |
| drug_use | **2.679744** | **.9089203** | **2.91** | **0.004** | **1.378419** | **5.209611** |
| smoker | **1.163819** | **.2310438** | **0.76** | **0.445** | **.7886845** | **1.717386** |
| psych | **1.236522** | **.3447728** | **0.76** | **0.446** | **.7159221** | **2.135688** |
| ac_coag | **1.500821** | **.2613981** | **2.33** | **0.020** | **1.06678** | **2.111459** |
| routine_steroid_use | **1.924209** | **.6990181** | **1.80** | **0.072** | **.944129** | **3.921687** |
| year_admit  2013 | **.8802337** | **.3976984** | **-0.28** | **0.778** | **.3630919** | **2.133926** |
| 2014 | **.9802167** | **.3487628** | **-0.06** | **0.955** | **.4880487** | **1.968707** |
| 2015 | **.7239893** | **.2775933** | **-0.84** | **0.400** | **.3414781** | **1.534975** |
| 2016 | **.8127996** | **.2943999** | **-0.57** | **0.567** | **.3996448** | **1.653076** |
| 2017 | **.8202027** | **.2433267** | **-0.67** | **0.504** | **.4585621** | **1.467048** |
| 2018 | **.6595233** | **.3324337** | **-0.83** | **0.409** | **.2455731** | **1.771249** |
| _cons | **.0013217** | **.0009731** | **-9.00** | **0.000** | **.0003122** | **.0055954** |

Note: **_cons** estimates baseline odds.

(65,021 observations deleted)

| Variable | Obs Mean | | Std. Dev. | Min | Max |  |
| --- | --- | --- | --- | --- | --- | --- |
| age | **41,120 80.18646** | | **8.783708** | **65** | **108.55** |  |
| **pneumonia** |  | |  |  |  |  |
| Iteration 0: | log pseudolikelihood = | | **-4416.8434** |  |  |  |
| Iteration 1: | log pseudolikelihood = | | **-3979.2438** |  |  |  |
| Iteration 2: | log pseudolikelihood = | | **-3830.4502** |  |  |  |
| Iteration 3: | log pseudolikelihood = | | **-3828.4899** |  |  |  |
| Iteration 4: | log pseudolikelihood = | | **-3828.4866** |  |  |  |
| Iteration 5: | log pseudolikelihood = | | **-3828.4866** |  |  |  |
| Logistic regression  Log pseudolikelihood | | = **-3828.4866** | Number of obs Wald chi2(33) Prob > chi2 Pseudo R2 | | =  =  =  = | **41,120**  **.**  **. 0.1332** |

(Std. Err. adjusted for **35** clusters in traumactr)

| pneumonia | Odds Ratio | Robust Std. Err. | z | P>\|z\| | [95% Conf. | Interval] |
| --- | --- | --- | --- | --- | --- | --- |
| diabetes | **1.256382** | **.1048365** | **2.74** | **0.006** | **1.066829** | **1.479615** |
| agecat 66-75 | **.774537** | **.1849675** | **-1.07** | **0.285** | **.4850275** | **1.236853** |
| >75 | **.6575154** | **.1643974** | **-1.68** | **0.094** | **.4027926** | **1.073323** |
| male | **2.184281** | **.169341** | **10.08** | **0.000** | **1.876365** | **2.542727** |
| isscat 16-24 | **2.358669** | **.2054912** | **9.85** | **0.000** | **1.988424** | **2.797855** |
| 25-35 | **3.262586** | **.3978027** | **9.70** | **0.000** | **2.569071** | **4.143313** |
| >35 | **3.572517** | **1.013077** | **4.49** | **0.000** | **2.049249** | **6.228075** |
| aishn | **1.464496** | **.1652308** | **3.38** | **0.001** | **1.173956** | **1.82694** |
| aisfac | **2.310086** | **1.031824** | **1.87** | **0.061** | **.9625647** | **5.54404** |
| aischs | **2.286174** | **.2271337** | **8.32** | **0.000** | **1.881662** | **2.777647** |
| aisabd | **1.880769** | **.2137318** | **5.56** | **0.000** | **1.505235** | **2.349993** |
| aisext | **1.352071** | **.1406746** | **2.90** | **0.004** | **1.102649** | **1.657913** |
| gcsmcat2  1 | **2.582057** | **.2778927** | **8.81** | **0.000** | **2.091008** | **3.188423** |
| 2 | **1.673314** | **.2699738** | **3.19** | **0.001** | **1.219674** | **2.295678** |
| 99 | **1.114306** | **.2039306** | **0.59** | **0.554** | **.7784397** | **1.595086** |
| pulse2 |  | | | | | |
| 1 | **1.208464** | **.222652** | **1.03** | **0.304** | **.8421799** | **1.734052** |
| 2 | **.5841466** | **.2515053** | **-1.25** | **0.212** | **.2512092** | **1.358339** |
| 99 | **.43261** | **.1735622** | **-2.09** | **0.037** | **.1970591** | **.9497226** |
| bp2 1 | **1.820294** | **.235883** | **4.62** | **0.000** | **1.412012** | **2.346629** |
| 2 | **.8744426** | **.3970895** | **-0.30** | **0.768** | **.3590837** | **2.129447** |
| 99 | **1.167139** | **.3565376** | **0.51** | **0.613** | **.6413539** | **2.123966** |
| race | **1.125169** | **.1346705** | **0.99** | **0.324** | **.889894** | **1.422648** |
| blunt_ind | **.8561264** | **.1338566** | **-0.99** | **0.320** | **.6301607** | **1.16312** |
| transfer | **1.020627** | **.1466148** | **0.14** | **0.887** | **.7701773** | **1.35252** |
| congestive_heart_failure | **1.497468** | **.2025736** | **2.98** | **0.003** | **1.148707** | **1.952117** |
| pvd | **1.244515** | **.2837567** | **0.96** | **0.337** | **.7960148** | **1.945714** |
| hypertension | **1.016736** | **.0754068** | **0.22** | **0.823** | **.8791807** | **1.175812** |
| dialysis | **.8138922** | **.1836364** | **-0.91** | **0.361** | **.523013** | **1.266547** |
| documented_history_of_cirrhosis | **1.071915** | **.4001324** | **0.19** | **0.852** | **.5157244** | **2.227939** |
| metastasis | **.9557422** | **.3101459** | **-0.14** | **0.889** | **.5059634** | **1.805354** |
| active_chemotherapy | **.6624657** | **.3038948** | **-0.90** | **0.369** | **.269581** | **1.627937** |
| obesity | **1.089872** | **.1725927** | **0.54** | **0.587** | **.7990587** | **1.486525** |
| ascites | **1.282035** | **1.954946** | **0.16** | **0.871** | **.0645537** | **25.46116** |
| drug_use | **1.298399** | **.2950087** | **1.15** | **0.250** | **.8317776** | **2.026793** |
| smoker | **1.571716** | **.1791254** | **3.97** | **0.000** | **1.257084** | **1.965096** |
| psych | **1.299472** | **.1132661** | **3.01** | **0.003** | **1.095402** | **1.54156** |
| ac_coag | **1.111944** | **.0807669** | **1.46** | **0.144** | **.9643957** | **1.282067** |
| routine_steroid_use | **1.050087** | **.2492956** | **0.21** | **0.837** | **.6593955** | **1.672263** |
| year_admit  2013 | **.8546044** | **.0955014** | **-1.41** | **0.160** | **.6865054** | **1.063864** |
| 2014 | **.7701167** | **.0940645** | **-2.14** | **0.032** | **.6061612** | **.9784192** |
| 2015 | **.7541259** | **.0930742** | **-2.29** | **0.022** | **.5920912** | **.9605038** |
| 2016 | **.5487356** | **.0956671** | **-3.44** | **0.001** | **.3899095** | **.772258** |
| 2017 | **.5291466** | **.0766467** | **-4.39** | **0.000** | **.3983636** | **.7028656** |
| 2018 | **.4889573** | **.0776415** | **-4.51** | **0.000** | **.358186** | **.6674723** |
| _cons | **.0113531** | **.0038884** | **-13.08** | **0.000** | **.0058021** | **.0222151** |

Note: **_cons** estimates baseline odds.

(65,021 observations deleted)

| Variable | Obs | Mean | Std. Dev. | Min | Max |
| --- | --- | --- | --- | --- | --- |
| age | **41,120** | **80.18646** | **8.783708** | **65** | **108.55** |
| **comp_cardiac** |  |  |  |  |  |

note: ascites != 0 predicts failure perfectly ascites dropped and 16 obs not used

Iteration 0: log pseudolikelihood = **-3406.2074**

Iteration 1: log pseudolikelihood = **-3297.5792**

Iteration 2: log pseudolikelihood = **-3106.84**

Iteration 3: log pseudolikelihood = **-3103.6201**

Iteration 4: log pseudolikelihood = **-3103.6112**

Iteration 5: log pseudolikelihood = **-3103.6112**

Logistic regression Number of obs = **41,104**

Wald chi2(33) = **.**

Prob > chi2 = **.**

Log pseudolikelihood = **-3103.6112** Pseudo R2 = **0.0888**

(Std. Err. adjusted for **35** clusters in traumactr)

comp_cardiac

diabetes

Robust

Odds Ratio Std. Err. z P>|z| [95% Conf. Interval]

# 1.254586 .1078348 2.64 0.008 1.060077 1.484784

agecat

| 66-75 | **1.002925** | **.411379** | **0.01** | **0.994** | **.4488726** | **2.240854** |
| --- | --- | --- | --- | --- | --- | --- |
| >75 | **1.310915** | **.5260275** | **0.67** | **0.500** | **.5970556** | **2.878286** |
| male | **1.600345** | **.1302378** | **5.78** | **0.000** | **1.364401** | **1.877091** |
| isscat 16-24 | **2.26681** | **.2788858** | **6.65** | **0.000** | **1.781114** | **2.884951** |
| 25-35 | **2.597321** | **.447875** | **5.54** | **0.000** | **1.852451** | **3.641703** |
| >35 | **6.010687** | **.9841416** | **10.95** | **0.000** | **4.36069** | **8.28501** |
| aishn | **.7458305** | **.1124697** | **-1.94** | **0.052** | **.5549843** | **1.002304** |
| aisfac | **1.881552** | **1.030747** | **1.15** | **0.249** | **.6430033** | **5.505784** |
| aischs | **1.421927** | **.1893792** | **2.64** | **0.008** | **1.095242** | **1.846055** |
| aisabd | **1.60191** | **.2377006** | **3.18** | **0.001** | **1.197655** | **2.142615** |
| aisext | **2.230846** | **.1710816** | **10.46** | **0.000** | **1.919516** | **2.592671** |
| gcsmcat2  1 | **1.922089** | **.370943** | **3.39** | **0.001** | **1.316741** | **2.805733** |
| 2 | **2.981324** | **.5442701** | **5.98** | **0.000** | **2.084558** | **4.263875** |
| 99 | **1.066175** | **.1798363** | **0.38** | **0.704** | **.7660421** | **1.483899** |
| pulse2 |  | | | | | |
| 1 | **1.642387** | **.2811398** | **2.90** | **0.004** | **1.174273** | **2.297112** |
| 2 | **1.422704** | **.4436754** | **1.13** | **0.258** | **.7720835** | **2.62159** |
| 99 | **.7096089** | **.3544094** | **-0.69** | **0.492** | **.2666197** | **1.888626** |
| bp2 1 | **2.169953** | **.4033072** | **4.17** | **0.000** | **1.507461** | **3.123594** |
| 2 | **2.963132** | **1.206449** | **2.67** | **0.008** | **1.334079** | **6.581436** |
| 99 | **1.484405** | **.6369866** | **0.92** | **0.357** | **.6401555** | **3.442067** |
| race | **1.271297** | **.1535213** | **1.99** | **0.047** | **1.003359** | **1.610786** |
| blunt_ind | **.8616476** | **.3536371** | **-0.36** | **0.717** | **.3854604** | **1.926103** |

| transfer | **.8079685** | **.118392** | **-1.46** | **0.146** | **.6062719** | **1.076766** |
| --- | --- | --- | --- | --- | --- | --- |
| congestive_heart_failure | **1.369677** | **.1594888** | **2.70** | **0.007** | **1.090189** | **1.720815** |
| pvd | **1.459232** | **.2968187** | **1.86** | **0.063** | **.979453** | **2.174028** |
| hypertension | **1.068851** | **.1032822** | **0.69** | **0.491** | **.8844358** | **1.291719** |
| dialysis | **1.909102** | **.4793192** | **2.58** | **0.010** | **1.167126** | **3.122775** |
| documented_history_of_cirrhosis | **1.771459** | **.5060809** | **2.00** | **0.045** | **1.01194** | **3.10104** |
| metastasis | **.5272241** | **.288071** | **-1.17** | **0.241** | **.1806781** | **1.538456** |
| active_chemotherapy | **.90693** | **.4285982** | **-0.21** | **0.836** | **.3591797** | **2.290001** |
| obesity | **1.117441** | **.1682085** | **0.74** | **0.461** | **.831942** | **1.500914** |
| ascites | **1** | (omitted) |  |  |  |  |
| drug_use | **.6746482** | **.1961908** | **-1.35** | **0.176** | **.3815434** | **1.192918** |
| smoker | **.8877095** | **.1366427** | **-0.77** | **0.439** | **.6565198** | **1.200312** |
| psych | **.8990549** | **.1455211** | **-0.66** | **0.511** | **.6546523** | **1.234701** |
| ac_coag | **1.263825** | **.1034297** | **2.86** | **0.004** | **1.076529** | **1.483707** |
| routine_steroid_use | **1.688247** | **.3725898** | **2.37** | **0.018** | **1.095416** | **2.601914** |
| year_admit  2013 | **.934796** | **.2180042** | **-0.29** | **0.772** | **.5918447** | **1.476475** |
| 2014 | **.9117454** | **.1460543** | **-0.58** | **0.564** | **.6660671** | **1.248042** |
| 2015 | **.9863352** | **.1374813** | **-0.10** | **0.921** | **.7505489** | **1.296194** |
| 2016 | **.8292192** | **.1414799** | **-1.10** | **0.272** | **.5935248** | **1.15851** |
| 2017 | **.7499315** | **.1434862** | **-1.50** | **0.133** | **.5154175** | **1.091149** |
| 2018 | **.9749763** | **.182092** | **-0.14** | **0.892** | **.6761124** | **1.405948** |
| _cons | **.0055061** | **.0033775** | **-8.48** | **0.000** | **.0016546** | **.0183225** |

Note: **_cons** estimates baseline odds.

(65,021 observations deleted)

Variable

age

# acute_renal_failure

Obs Mean Std. Dev. Min Max

# 41,120 80.18646 8.783708 65 108.55

note: aisfac != 0 predicts failure perfectly aisfac dropped and 80 obs not used

note: ascites != 0 predicts failure perfectly ascites dropped and 16 obs not used

Iteration 0: log pseudolikelihood = **-1406.0813**

Iteration 1: log pseudolikelihood = **-1332.9279**

Iteration 2: log pseudolikelihood = **-1254.0797**

Iteration 3: log pseudolikelihood = **-1253.1331**

Iteration 4: log pseudolikelihood = **-1253.1292**

Iteration 5: log pseudolikelihood = **-1253.1292**

Logistic regression Number of obs = **41,024**

Wald chi2(33) = **.**

Prob > chi2 = **.**

Log pseudolikelihood = **-1253.1292** Pseudo R2 = **0.1088**

(Std. Err. adjusted for **35** clusters in traumactr)

acute_renal_failure

diabetes

Robust

Odds Ratio Std. Err. z P>|z| [95% Conf. Interval]

# 1.698026 .2447172 3.67 0.000 1.280178 2.252259

agecat 66-75

>75

male

# 1.801107 .9047063 1.17 0.241 .6729396 4.82062

**1.600022 .7457733 1.01 0.313 .6417702 3.989079**

**2.002473 .280225 4.96 0.000 1.522124 2.63441**

| isscat  16-24 | **2.241481** | **.5151112** | **3.51** | **0.000** | **1.428636** | **3.516805** |
| --- | --- | --- | --- | --- | --- | --- |
| 25-35 | **3.753721** | **1.076324** | **4.61** | **0.000** | **2.139895** | **6.584632** |
| >35 | **3.167942** | **1.651618** | **2.21** | **0.027** | **1.14025** | **8.801448** |
| aishn | **.5180283** | **.1674234** | **-2.04** | **0.042** | **.274948** | **.9760146** |
| aisfac | **1** | (omitted) |  |  |  |  |
| aischs | **1.951319** | **.3545271** | **3.68** | **0.000** | **1.366711** | **2.78599** |
| aisabd | **3.197085** | **1.03807** | **3.58** | **0.000** | **1.691902** | **6.041337** |
| aisext | **2.455192** | **.4305062** | **5.12** | **0.000** | **1.741131** | **3.4621** |
| gcsmcat2  1 | **1.380436** | **.5036775** | **0.88** | **0.377** | **.6752116** | **2.822233** |
| 2 | **1.291521** | **.3277644** | **1.01** | **0.313** | **.7853843** | **2.123835** |
| 99 | **.8390987** | **.2637144** | **-0.56** | **0.577** | **.4532052** | **1.553571** |
| pulse2 |  |  |  |  |  |  |
| 1 | **.6947214** | **.2787225** | **-0.91** | **0.364** | **.3164523** | **1.525152** |
| 2 | **.9637083** | **.5036111** | **-0.07** | **0.944** | **.3460413** | **2.683881** |
| 99 | **.9931987** | **.7923583** | **-0.01** | **0.993** | **.207951** | **4.743636** |
| bp2 1 | **1.843708** | **.4359444** | **2.59** | **0.010** | **1.159914** | **2.930614** |
| 2 | **2.646244** | **1.426922** | **1.80** | **0.071** | **.9196881** | **7.614114** |
| 99 | **1.080934** | **.7869429** | **0.11** | **0.915** | **.2594813** | **4.502897** |
| race | **1.336941** | **.3931996** | **0.99** | **0.323** | **.7512259** | **2.379326** |
| blunt_ind | **1.270762** | **.7347932** | **0.41** | **0.679** | **.4091419** | **3.946888** |
| transfer | **1.280428** | **.2114918** | **1.50** | **0.135** | **.9263178** | **1.769907** |
| congestive_heart_failure | **1.523052** | **.5039471** | **1.27** | **0.204** | **.7962869** | **2.913132** |
| pvd | **1.304965** | **.5203315** | **0.67** | **0.504** | **.5973069** | **2.85102** |
| hypertension | **1.15014** | **.1810188** | **0.89** | **0.374** | **.8448534** | **1.565741** |
| dialysis | **1.294591** | **.3923483** | **0.85** | **0.394** | **.7147619** | **2.34479** |
| documented_history_of_cirrhosis | **3.380465** | **1.586455** | **2.60** | **0.009** | **1.347432** | **8.480982** |
| metastasis | **.981898** | **.6433399** | **-0.03** | **0.978** | **.2718685** | **3.546288** |
| active_chemotherapy | **1.311349** | **.9967179** | **0.36** | **0.721** | **.2956273** | **5.816901** |
| obesity | **1.441143** | **.311936** | **1.69** | **0.091** | **.9428963** | **2.202673** |
| ascites | **1** | (omitted) |  |  |  |  |
| drug_use | **.6864385** | **.330576** | **-0.78** | **0.435** | **.2671031** | **1.764105** |
| smoker | **1.211578** | **.2839908** | **0.82** | **0.413** | **.7653007** | **1.918099** |
| psych | **.902918** | **.2717032** | **-0.34** | **0.734** | **.5006192** | **1.628505** |
| ac_coag | **1.203682** | **.1663726** | **1.34** | **0.180** | **.9180342** | **1.578209** |
| routine_steroid_use | **1.543805** | **.5917924** | **1.13** | **0.257** | **.7282799** | **3.272553** |
| year_admit  2013 | **.5998427** | **.1524403** | **-2.01** | **0.044** | **.3645174** | **.9870893** |
| 2014 | **.7188209** | **.3239015** | **-0.73** | **0.464** | **.2972134** | **1.738493** |
| 2015 | **.9033395** | **.3608105** | **-0.25** | **0.799** | **.4129195** | **1.976226** |
| 2016 | **.9553806** | **.4814966** | **-0.09** | **0.928** | **.3557824** | **2.565479** |
| 2017 | **.6179138** | **.2624977** | **-1.13** | **0.257** | **.2687365** | **1.420788** |
| 2018 | **.496231** | **.2644033** | **-1.32** | **0.188** | **.1746401** | **1.410015** |
| _cons | **.0007447** | **.0006821** | **-7.86** | **0.000** | **.0001237** | **.0044832** |

Note: **_cons** estimates baseline odds.

(65,021 observations deleted)

Variable

age

Obs Mean Std. Dev. Min Max

# 41,120 80.18646 8.783708 65 108.55

**comp_vte**

note: ascites != 0 predicts failure perfectly ascites dropped and 16 obs not used

Iteration 0: log pseudolikelihood = **-2324.6488**

Iteration 1: log pseudolikelihood = **-2189.9383**

Iteration 2: log pseudolikelihood = **-2160.4633**

Iteration 3: log pseudolikelihood = **-2159.8847**

Iteration 4: log pseudolikelihood = **-2159.8835**

Iteration 5: log pseudolikelihood = **-2159.8835**

| Logistic regression  Log pseudolikelihood | = | Number of obs Wald chi2(33) Prob > chi2  **-2159.8835** Pseudo R2 | | | | =  =  =  = | **41,104**  **.**  **. 0.0709** | | |
| --- | --- | --- | --- | --- | --- | --- | --- | --- | --- |
| (Std. Err. adjusted for **35** clusters in traumactr) | | | | | | | | | |
| comp_vte | | | Odds Ratio | Robust Std. Err. | z | P>\|z\| | | [95% Conf. | Interval] |
| diabetes | | | **1.11726** | **.1482904** | **0.84** | **0.403** | | **.8613442** | **1.44921** |
| agecat 66-75 | | | **.8730329** | **.2803243** | **-0.42** | **0.672** | | **.4652819** | **1.638117** |
| >75 | | | **.6203234** | **.1976162** | **-1.50** | **0.134** | | **.3322395** | **1.158204** |
| male | | | **1.460965** | **.168201** | **3.29** | **0.001** | | **1.165846** | **1.830791** |
| isscat 16-24 | | | **2.36714** | **.4027749** | **5.06** | **0.000** | | **1.69586** | **3.304137** |
| 25-35 | | | **3.112326** | **.6294343** | **5.61** | **0.000** | | **2.093817** | **4.626274** |
| >35 | | | **3.605988** | **1.263995** | **3.66** | **0.000** | | **1.814076** | **7.167919** |
| aishn | | | **1.031426** | **.19578** | **0.16** | **0.871** | | **.7109962** | **1.496266** |
| aisfac | | | **.6375035** | **.6103022** | **-0.47** | **0.638** | | **.0976338** | **4.1626** |
| aischs | | | **1.515249** | **.1808929** | **3.48** | **0.000** | | **1.19913** | **1.914705** |
| aisabd | | | **1.044518** | **.2898816** | **0.16** | **0.875** | | **.6062962** | **1.79948** |
| aisext | | | **2.304609** | **.3165799** | **6.08** | **0.000** | | **1.760635** | **3.016652** |
| gcsmcat2  1 | | | **1.767591** | **.3160869** | **3.19** | **0.001** | | **1.244993** | **2.509555** |
| 2 | | | **.8643672** | **.2954895** | **-0.43** | **0.670** | | **.4422925** | **1.689223** |
| 99 | | | **.74531** | **.1642702** | **-1.33** | **0.182** | | **.4838689** | **1.148011** |
| pulse2 | | |  | | | | | | |
| 1 | | | **1.501119** | **.3235343** | **1.88** | **0.059** | | **.9839128** | **2.2902** |
| 2 | | | **1.202182** | **.4181054** | **0.53** | **0.596** | | **.6080394** | **2.376887** |
| 99 | | | **1.134316** | **.5298546** | **0.27** | **0.787** | | **.4540733** | **2.833623** |
| bp2 1 | | | **1.833213** | **.5019024** | **2.21** | **0.027** | | **1.071935** | **3.135143** |
| 2 | | | **.3367825** | **.3218594** | **-1.14** | **0.255** | | **.0517446** | **2.191965** |
| 99 | | | **.5859763** | **.3395881** | **-0.92** | **0.356** | | **.188186** | **1.824622** |
| race | | | **1.472829** | **.1714089** | **3.33** | **0.001** | | **1.172436** | **1.850188** |
| blunt_ind | | | **.4795831** | **.1305631** | **-2.70** | **0.007** | | **.2812745** | **.8177064** |
| transfer | | | **1.240233** | **.1777541** | **1.50** | **0.133** | | **.9364974** | **1.64248** |
| congestive_heart_failure | | | **.7872796** | **.2011208** | **-0.94** | **0.349** | | **.4771758** | **1.298911** |
| pvd | | | **1.269784** | **.442933** | **0.68** | **0.494** | | **.6409281** | **2.515652** |
| hypertension | | | **.9864892** | **.1198803** | **-0.11** | **0.911** | | **.7774142** | **1.251792** |
| dialysis | | | **.6164144** | **.322293** | **-0.93** | **0.355** | | **.2212183** | **1.71761** |
| documented_history_of_cirrhosis | | | **.8416346** | **.4728809** | **-0.31** | **0.759** | | **.2798127** | **2.531511** |
| metastasis | | | **1.507171** | **.7033064** | **0.88** | **0.379** | | **.6038901** | **3.761554** |
| active_chemotherapy | | | **1.837218** | **1.024449** | **1.09** | **0.275** | | **.6159186** | **5.480222** |
| obesity | | | **1.870027** | **.3013613** | **3.88** | **0.000** | | **1.363559** | **2.564614** |
| ascites | | | **1** | (omitted) |  |  | |  |  |
| drug_use | | | **1.145999** | **.3725839** | **0.42** | **0.675** | | **.6059599** | **2.167327** |

| smoker | **1.039734** | **.1480576** | **0.27** | **0.784** | **.7865237** | **1.374463** |
| --- | --- | --- | --- | --- | --- | --- |
| psych | **.9640717** | **.1359858** | **-0.26** | **0.795** | **.7312136** | **1.271084** |
| ac_coag | **.9147271** | **.1178861** | **-0.69** | **0.489** | **.7105462** | **1.177581** |
| routine_steroid_use | **.5374892** | **.183849** | **-1.82** | **0.070** | **.2749255** | **1.050811** |
| year_admit  2013 | **.9335283** | **.2006773** | **-0.32** | **0.749** | **.6125587** | **1.42268** |
| 2014 | **.9820191** | **.2263803** | **-0.08** | **0.937** | **.6250236** | **1.54292** |
| 2015 | **1.052745** | **.2569753** | **0.21** | **0.833** | **.652446** | **1.698641** |
| 2016 | **.951542** | **.203008** | **-0.23** | **0.816** | **.6263646** | **1.445535** |
| 2017 | **.8444732** | **.1695848** | **-0.84** | **0.400** | **.5697049** | **1.251762** |
| 2018 | **.6008758** | **.1456932** | **-2.10** | **0.036** | **.3735903** | **.9664379** |
| _cons | **.0123102** | **.0052592** | **-10.29** | **0.000** | **.0053286** | **.028439** |

Note: **_cons** estimates baseline odds.

**Variance inflation factors

| 129 | . |
| --- | --- |
| 130 | . |
| 131 | . |
| 132 | . |
| 133 | . |
| 134 | . |

**Will be the same for all outcomes; only need to run for one outcome

regress comp_any diabetes i.agecat male i.isscat aishn aisfac aischs aisabd aisext i.gcsmcat2 i.

| Source | SS | df | MS |
| --- | --- | --- | --- |
| Model | **949.681458** | **46** | **20.6452491** |
| Residual | **6642.17777** | **106,094** | **.062606535** |
| Total | **7591.85922** | **106,140** | **.071526844** |

| Number of obs | | | | = | **106,141** | | |
| --- | --- | --- | --- | --- | --- | --- | --- |
| F(46, 106094) | | | | = | **329.76** | | |
| Prob > F | | | | = | **0.0000** | | |
| R-squared | | | | = | **0.1251** | | |
| Adj R-squared | | | | = | **0.1247** | | |
| Root MSE | | | | = | **.25021** | | |
| comp_any | Coef. | Std. Err. | t | P>\|t\| | | [95% Conf. | Interval] |
| diabetes | **.0155909** | **.0024195** | **6.44** | **0.000** | | **.0108487** | **.0203332** |
| agecat 26-45 | **.0085343** | **.0027538** | **3.10** | **0.002** | | **.0031368** | **.0139318** |
| 46-65 | **.0274432** | **.0027759** | **9.89** | **0.000** | | **.0220024** | **.0328841** |
| 66-75 | **.0384426** | **.0034504** | **11.14** | **0.000** | | **.0316798** | **.0452054** |
| >75 | **.0321242** | **.0032348** | **9.93** | **0.000** | | **.025784** | **.0384644** |
| male | **.0146321** | **.0016871** | **8.67** | **0.000** | | **.0113255** | **.0179387** |
| isscat 16-24 | **.052403** | **.0025448** | **20.59** | **0.000** | | **.0474153** | **.0573907** |
| 25-35 | **.1435079** | **.003684** | **38.95** | **0.000** | | **.1362873** | **.1507285** |
| >35 | **.237514** | **.0068023** | **34.92** | **0.000** | | **.2241817** | **.2508464** |
| aishn | **.012841** | **.0023489** | **5.47** | **0.000** | | **.0082371** | **.0174449** |
| aisfac | **.0350049** | **.0108631** | **3.22** | **0.001** | | **.0137134** | **.0562965** |
| aischs | **.0343515** | **.0021257** | **16.16** | **0.000** | | **.0301852** | **.0385178** |
| aisabd | **.0599141** | **.0035206** | **17.02** | **0.000** | | **.0530138** | **.0668144** |
| aisext | **.0440823** | **.0020864** | **21.13** | **0.000** | | **.0399929** | **.0481717** |
| gcsmcat2  1 | **.1166063** | **.0036418** | **32.02** | **0.000** | | **.1094684** | **.1237442** |
| 2 | **.1276399** | **.004349** | **29.35** | **0.000** | | **.1191159** | **.1361638** |
| 99 | **.014848** | **.0032702** | **4.54** | **0.000** | | **.0084385** | **.0212575** |
| pulse2 |  | | | | | | |
| 1 | **.0568186** | **.0034756** | **16.35** | **0.000** | | **.0500064** | **.0636308** |
| 2 | **.0128154** | **.0078746** | **1.63** | **0.104** | | **-.0026187** | **.0282494** |
| 99 | **-.0473702** | **.0088491** | **-5.35** | **0.000** | | **-.0647144** | **-.0300261** |

| bp2 1 | **.0770551** | **.0049348** | **15.61** | **0.000** | **.0673829** | **.0867272** |
| --- | --- | --- | --- | --- | --- | --- |
| 2 | **.0362377** | **.0110278** | **3.29** | **0.001** | **.0146233** | **.0578521** |
| 99 | **.0291739** | **.0084286** | **3.46** | **0.001** | **.0126539** | **.0456938** |
| race | **.0062818** | **.0019716** | **3.19** | **0.001** | **.0024176** | **.010146** |
| blunt_ind | **-.0201854** | **.003248** | **-6.21** | **0.000** | **-.0265514** | **-.0138194** |
| transfer | **.0094108** | **.0020397** | **4.61** | **0.000** | **.005413** | **.0134086** |
| congestive_heart_failure | **.0299777** | **.004514** | **6.64** | **0.000** | **.0211303** | **.0388251** |
| pvd | **.0141953** | **.0083593** | **1.70** | **0.089** | **-.0021888** | **.0305793** |
| hypertension | **.0027155** | **.0019651** | **1.38** | **0.167** | **-.0011361** | **.0065671** |
| dialysis | **.0299061** | **.0074334** | **4.02** | **0.000** | **.0153368** | **.0444754** |
| documented_history_of_cirrhosis | **.0700938** | **.0093473** | **7.50** | **0.000** | **.0517731** | **.0884144** |
| metastasis | **.0081014** | **.0126047** | **0.64** | **0.520** | **-.0166037** | **.0328065** |
| active_chemotherapy | **-.003967** | **.0132713** | **-0.30** | **0.765** | **-.0299786** | **.0220446** |
| obesity | **.0228506** | **.0030718** | **7.44** | **0.000** | **.0168298** | **.0288713** |
| ascites | **.0394563** | **.040574** | **0.97** | **0.331** | **-.0400681** | **.1189808** |
| drug_use | **.0053578** | **.0024738** | **2.17** | **0.030** | **.0005091** | **.0102065** |
| smoker | **.0033105** | **.0019286** | **1.72** | **0.086** | **-.0004696** | **.0070905** |
| psych | **.0067241** | **.0021418** | **3.14** | **0.002** | **.0025262** | **.010922** |
| ac_coag | **.0126028** | **.0025426** | **4.96** | **0.000** | **.0076193** | **.0175863** |
| routine_steroid_use | **.0191461** | **.0063093** | **3.03** | **0.002** | **.0067799** | **.0315122** |
| year_admit  2013 | **-.0053154** | **.0032439** | **-1.64** | **0.101** | **-.0116733** | **.0010426** |
| 2014 | **-.006978** | **.0032261** | **-2.16** | **0.031** | **-.0133012** | **-.0006548** |
| 2015 | **-.0074243** | **.0031972** | **-2.32** | **0.020** | **-.0136908** | **-.0011579** |
| 2016 | **-.0145074** | **.0030777** | **-4.71** | **0.000** | **-.0205395** | **-.0084752** |
| 2017 | **-.0204533** | **.0030339** | **-6.74** | **0.000** | **-.0263998** | **-.0145068** |
| 2018 | **-.0253189** | **.0030537** | **-8.29** | **0.000** | **-.031304** | **-.0193337** |
| _cons | **-.0004026** | **.0046637** | **-0.09** | **0.931** | **-.0095433** | **.0087381** |

135 . estat vif

| Variable | VIF | 1/VIF |
| --- | --- | --- |
| diabetes | **1.15** | **0.872051** |
| agecat  1 | **2.16** | **0.463735** |
| 2 | **2.63** | **0.380197** |
| 3 | **2.16** | **0.462041** |
| 4 | **3.41** | **0.293269** |
| male | **1.15** | **0.866643** |
| isscat  1 | **1.41** | **0.707959** |
| 2 | **1.56** | **0.642716** |
| 3 | **1.46** | **0.685147** |
| aishn | **1.74** | **0.575797** |
| aisfac | **1.02** | **0.980263** |
| aischs | **1.32** | **0.759940** |
| aisabd | **1.23** | **0.814230** |
| aisext | **1.17** | **0.853602** |
| gcsmcat2  1 | **1.09** | **0.915569** |
| 2 | **1.30** | **0.770605** |
| 99  pulse2 | **1.15** | **0.868919** |
| 1 | **1.07** | **0.934892** |
| 2 | **1.02** | **0.983223** |
| 99 | **3.26** | **0.306495** |
| bp2  1 | **1.05** | **0.953319** |
| 2 | **1.03** | **0.967118** |
| 99 | **3.25** | **0.307376** |
| race | **1.22** | **0.822900** |

| blunt_ind | **1.23** | **0.813330** |
| --- | --- | --- |
| transfer | **1.05** | **0.950485** |
| congestive~e | **1.08** | **0.930155** |
| pvd | **1.02** | **0.976062** |
| hypertension | **1.53** | **0.653581** |
| dialysis | **1.03** | **0.971817** |
| documented~s | **1.03** | **0.969900** |
| metastasis | **1.07** | **0.937498** |
| active_che~y | **1.07** | **0.938782** |
| obesity | **1.05** | **0.953784** |
| ascites | **1.03** | **0.975483** |
| drug_use | **1.23** | **0.815024** |
| smoker | **1.22** | **0.817283** |
| psych | **1.06** | **0.941495** |
| ac_coag | **1.20** | **0.832328** |
| routine_st~e | **1.01** | **0.985873** |
| year_admit |  |  |
| 2013 | **1.90** | **0.525516** |
| 2014 | **1.94** | **0.516131** |
| 2015 | **1.98** | **0.503886** |
| 2016 | **2.18** | **0.459395** |
| 2017 | **2.29** | **0.437593** |
| 2018 | **2.29** | **0.436498** |
| Mean VIF | **1.51** | |

136 .

1. . regress hospdays diabetes i.agecat male i.isscat aishn aisfac aischs aisabd aisext i.gcsmcat2 i.

| Source | SS | df | MS |
| --- | --- | --- | --- |
| Model | **789549.604** | **46** | **17164.1218** |
| Residual | **4364276.34** | **105,771** | **41.2615589** |
| Total | **5153825.94** | **105,817** | **48.7050847** |

| Number of obs | | | | = | **105,818** | | |
| --- | --- | --- | --- | --- | --- | --- | --- |
| F(46, 105771) | | | | = | **415.98** | | |
| Prob > F | | | | = | **0.0000** | | |
| R-squared | | | | = | **0.1532** | | |
| Adj R-squared | | | | = | **0.1528** | | |
| Root MSE | | | | = | **6.4235** | | |
| hospdays | Coef. | Std. Err. | t | P>\|t\| | | [95% Conf. | Interval] |
| diabetes | **.3497445** | **.0621565** | **5.63** | **0.000** | | **.2279187** | **.4715704** |
| agecat 26-45 | **.460897** | **.0708553** | **6.50** | **0.000** | | **.3220215** | **.5997725** |
| 46-65 | **1.128717** | **.0714204** | **15.80** | **0.000** | | **.9887339** | **1.2687** |
| 66-75 | **1.148028** | **.0887332** | **12.94** | **0.000** | | **.9741121** | **1.321944** |
| >75 | **.837949** | **.0832149** | **10.07** | **0.000** | | **.6748488** | **1.001049** |
| male | **.3276083** | **.043362** | **7.56** | **0.000** | | **.2426193** | **.4125972** |
| isscat 16-24 | **2.233613** | **.0654537** | **34.13** | **0.000** | | **2.105325** | **2.361902** |
| 25-35 | **3.884153** | **.0951023** | **40.84** | **0.000** | | **3.697754** | **4.070552** |
| >35 | **4.816078** | **.177251** | **27.17** | **0.000** | | **4.468668** | **5.163487** |
| aishn | **.6579725** | **.0604734** | **10.88** | **0.000** | | **.5394456** | **.7764995** |
| aisfac | **3.049795** | **.279692** | **10.90** | **0.000** | | **2.501602** | **3.597987** |
| aischs | **1.365576** | **.0546897** | **24.97** | **0.000** | | **1.258384** | **1.472767** |
| aisabd | **2.228193** | **.0907981** | **24.54** | **0.000** | | **2.05023** | **2.406156** |
| aisext | **2.625678** | **.0536924** | **48.90** | **0.000** | | **2.520442** | **2.730915** |
| gcsmcat2  1 | **3.347383** | **.0936914** | **35.73** | **0.000** | | **3.16375** | **3.531017** |
| 2 | **1.861777** | **.1138452** | **16.35** | **0.000** | | **1.638642** | **2.084912** |
| 99 | **.4267033** | **.0842522** | **5.06** | **0.000** | | **.2615701** | **.5918365** |
| pulse2  1 | **1.508527 .0894763 16.86 0.000 1.333154 1.683899** | | | | | | |

| 2 | **.0735288** | **.2041466** | **0.36** | **0.719** | **-.3265957** | **.4736533** |
| --- | --- | --- | --- | --- | --- | --- |
| 99 | **-.6491293** | **.230115** | **-2.82** | **0.005** | **-1.100151** | **-.1981071** |
| bp2 1 | **1.70643** | **.1272443** | **13.41** | **0.000** | **1.457033** | **1.955827** |
| 2 | **-1.07101** | **.2874434** | **-3.73** | **0.000** | **-1.634395** | **-.5076248** |
| 99 | **.5777825** | **.2183357** | **2.65** | **0.008** | **.1498475** | **1.005717** |
| race | **.3450812** | **.0507236** | **6.80** | **0.000** | **.2456637** | **.4444986** |
| blunt_ind | **-.7461916** | **.0839998** | **-8.88** | **0.000** | **-.9108301** | **-.5815531** |
| transfer | **.595048** | **.0524155** | **11.35** | **0.000** | **.4923143** | **.6977817** |
| congestive_heart_failure | **.7345677** | **.1159757** | **6.33** | **0.000** | **.5072569** | **.9618785** |
| pvd | **.4110022** | **.2147163** | **1.91** | **0.056** | **-.0098389** | **.8318433** |
| hypertension | **.3041049** | **.0505017** | **6.02** | **0.000** | **.2051223** | **.4030876** |
| dialysis | **.9204058** | **.1908364** | **4.82** | **0.000** | **.5463689** | **1.294443** |
| documented_history_of_cirrhosis | **1.684873** | **.2399718** | **7.02** | **0.000** | **1.214532** | **2.155215** |
| metastasis | **.1876977** | **.3240856** | **0.58** | **0.562** | **-.4475056** | **.822901** |
| active_chemotherapy | **-.0748698** | **.3421564** | **-0.22** | **0.827** | **-.7454917** | **.5957521** |
| obesity | **.5419949** | **.0789316** | **6.87** | **0.000** | **.3872902** | **.6966997** |
| ascites | **-.8135971** | **1.041628** | **-0.78** | **0.435** | **-2.855173** | **1.227979** |
| drug_use | **.3514** | **.0635642** | **5.53** | **0.000** | **.2268151** | **.4759849** |
| smoker | **.0728655** | **.0495496** | **1.47** | **0.141** | **-.024251** | **.169982** |
| psych | **.3725802** | **.0550173** | **6.77** | **0.000** | **.264747** | **.4804135** |
| ac_coag | **.2194395** | **.0653085** | **3.36** | **0.001** | **.0914357** | **.3474432** |
| routine_steroid_use | **.266393** | **.1620206** | **1.64** | **0.100** | **-.0511652** | **.5839513** |
| year_admit  2013 | **-.2721113** | **.083431** | **-3.26** | **0.001** | **-.4356349** | **-.1085877** |
| 2014 | **-.2879923** | **.0829619** | **-3.47** | **0.001** | **-.4505966** | **-.125388** |
| 2015 | **-.4537587** | **.0822152** | **-5.52** | **0.000** | **-.6148994** | **-.292618** |
| 2016 | **-.5079044** | **.0791481** | **-6.42** | **0.000** | **-.6630337** | **-.3527752** |
| 2017 | **-.739432** | **.0780234** | **-9.48** | **0.000** | **-.8923568** | **-.5865073** |
| 2018 | **-.859787** | **.078528** | **-10.95** | **0.000** | **-1.013701** | **-.7058731** |
| _cons | **2.768023** | **.1202459** | **23.02** | **0.000** | **2.532343** | **3.003703** |

1. . estat vif

| Variable | VIF | 1/VIF |
| --- | --- | --- |
| diabetes | **1.15** | **0.872219** |
| agecat  1 | **2.16** | **0.463555** |
| 2 | **2.63** | **0.379703** |
| 3 | **2.17** | **0.461378** |
| 4 | **3.42** | **0.292730** |
| male | **1.15** | **0.866889** |
| isscat  1 | **1.41** | **0.707976** |
| 2 | **1.55** | **0.646096** |
| 3 | **1.45** | **0.691708** |
| aishn | **1.74** | **0.575685** |
| aisfac | **1.02** | **0.979974** |
| aischs | **1.31** | **0.761607** |
| aisabd | **1.23** | **0.813590** |
| aisext | **1.17** | **0.852318** |
| gcsmcat2  1 | **1.09** | **0.915673** |
| 2 | **1.28** | **0.782187** |
| 99  pulse2 | **1.15** | **0.867500** |
| 1 | **1.07** | **0.934656** |
| 2 | **1.02** | **0.984875** |
| 99 | **3.15** | **0.317492** |
| bp2  1 | **1.05** | **0.953106** |

| 2 | **1.03** | **0.968819** |
| --- | --- | --- |
| 99 | **3.14** | **0.318971** |
| race | **1.21** | **0.823268** |
| blunt_ind | **1.23** | **0.815147** |
| transfer | **1.05** | **0.949876** |
| congestive~e | **1.08** | **0.930099** |
| pvd | **1.02** | **0.976083** |
| hypertension | **1.53** | **0.653596** |
| dialysis | **1.03** | **0.971796** |
| documented~s | **1.03** | **0.969877** |
| metastasis | **1.07** | **0.936862** |
| active_che~y | **1.07** | **0.938221** |
| obesity | **1.05** | **0.953697** |
| ascites | **1.03** | **0.975475** |
| drug_use | **1.23** | **0.814659** |
| smoker | **1.22** | **0.817323** |
| psych | **1.06** | **0.941732** |
| ac_coag | **1.20** | **0.832522** |
| routine_st~e | **1.01** | **0.985948** |
| year_admit |  |  |
| 2013 | **1.90** | **0.525453** |
| 2014 | **1.94** | **0.515881** |
| 2015 | **1.99** | **0.503700** |
| 2016 | **2.18** | **0.459231** |
| 2017 | **2.29** | **0.437307** |
| 2018 | **2.29** | **0.436212** |
| Mean VIF | **1.51** | |
